# Supplementary material for: Cross-serotypically conserved epitope recommendations for a universal T cell-based dengue vaccine
Source: PLoS Negl Trop Dis. 2020 Sep 21;14(9):e0008676. doi: 10.1371/journal.pntd.0008676 (PMC7529213; doi:10.1371/journal.pntd.0008676)
Supplement: S1 Fig — The locations of epitopes were determined by mapping them onto all: (A) DENV2, (B) DENV3, and (C) DENV4 sequences, respectively. The color scales in (A)-(C) indicate the HLA class restriction of the epitopes. (PDF) [file pntd.0008676.s001.pdf]

A

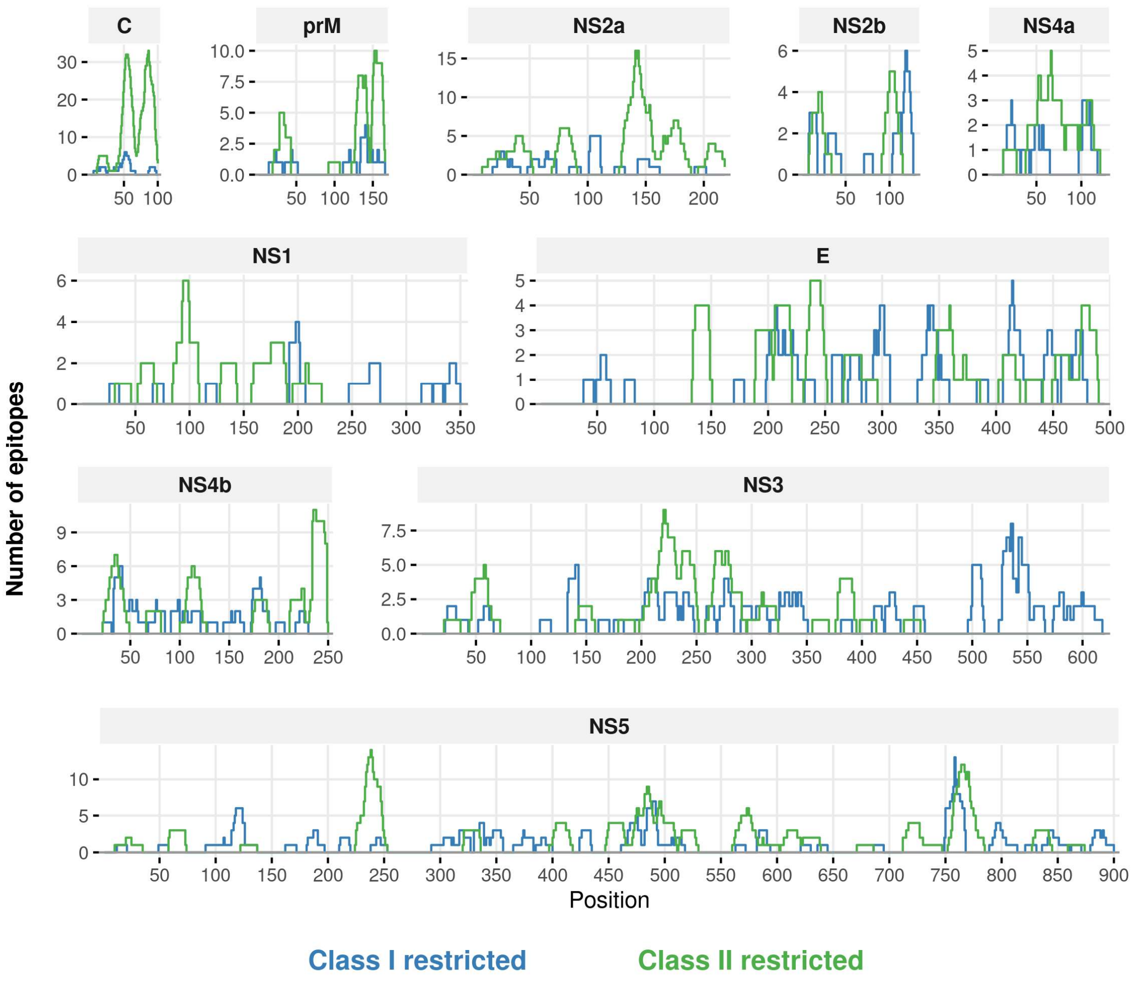
C
prM
NS2a
NS2b
NS4a
NS1
E
NS4b
NS3
NS5
Number of epitopes
Position
Class I restricted
Class II restricted

B

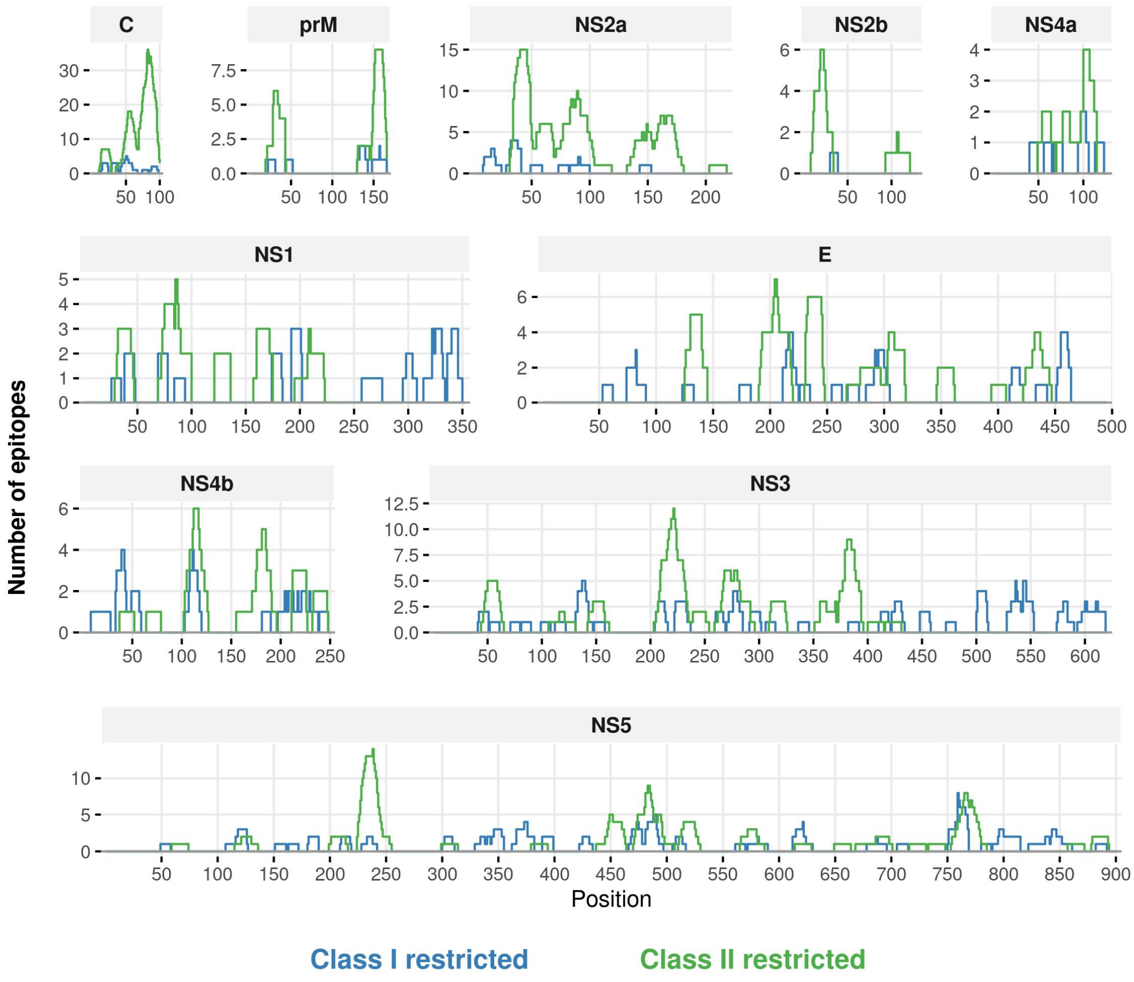

C
prM
NS2a
NS2b
NS4a
NS1
E
NS4b
NS3
NS5
Number of epitopes
Position
Class I restricted
Class II restricted

C

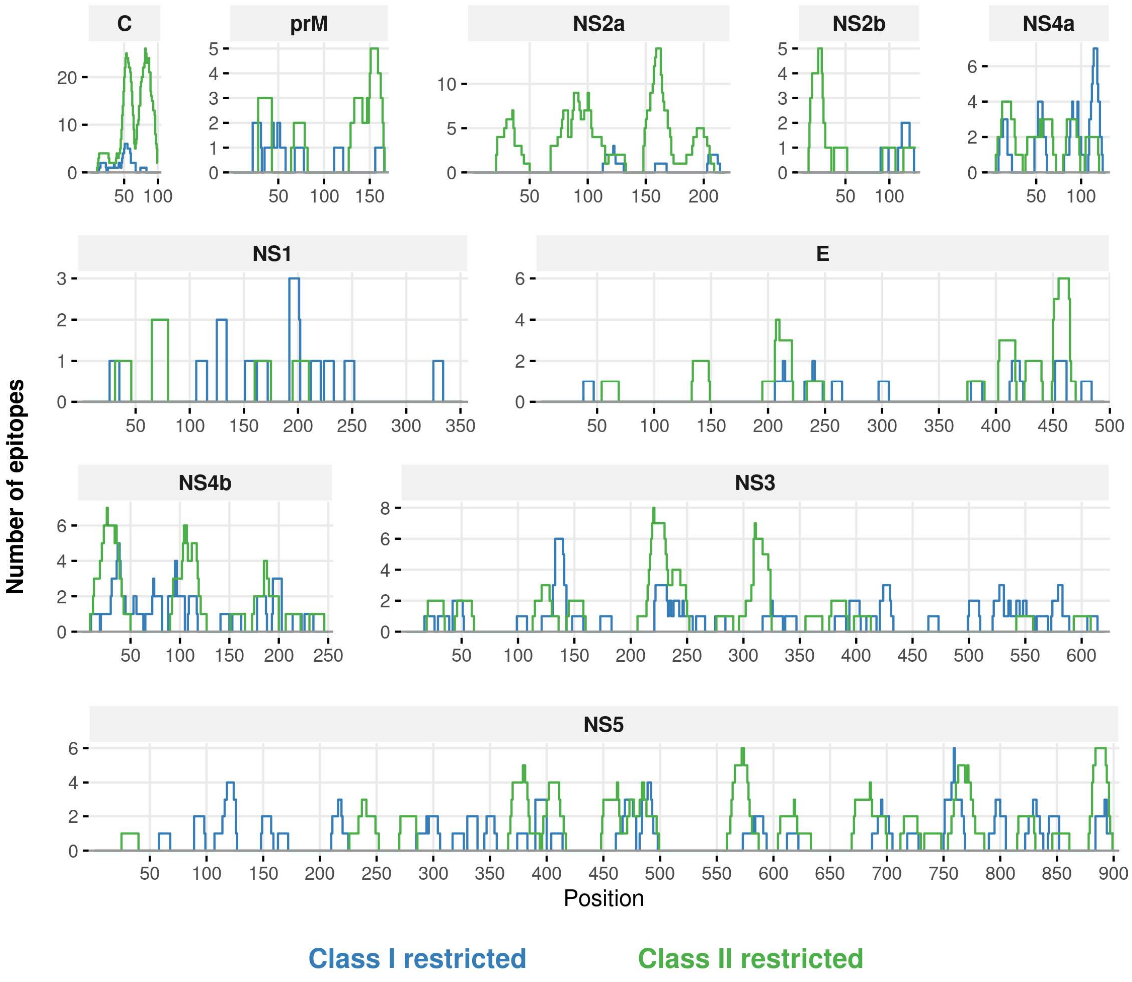

Fig S1. Coverage of DENV T cell epitopes across the primary structure of DENV proteins. The locations of epitopes were determined by mapping them onto all: **(A)** DENV2, **(B)** DENV3, and **(C)** DENV4 sequences, respectively. The color scales in (A)-(C) indicate the HLA class restriction of the epitopes.
