# Supplementary material for: Cross-serotypically conserved epitope recommendations for a universal T cell-based dengue vaccine
Source: PLoS Negl Trop Dis. 2020 Sep 21;14(9):e0008676. doi: 10.1371/journal.pntd.0008676 (PMC7529213; doi:10.1371/journal.pntd.0008676)
Supplement: S2 Fig — Sequence-logos of top epitopes that are not highly conserved in one of the four DENV serotypes for: (A) 15 epitopes of NS5, (B) 16 epitopes of NS3, and (C) 2 epitopes of E (top panel) and 3 epitopes of NS4b (bottom panel). (PDF) [file pntd.0008676.s002.pdf]

Supplementary Figure

A

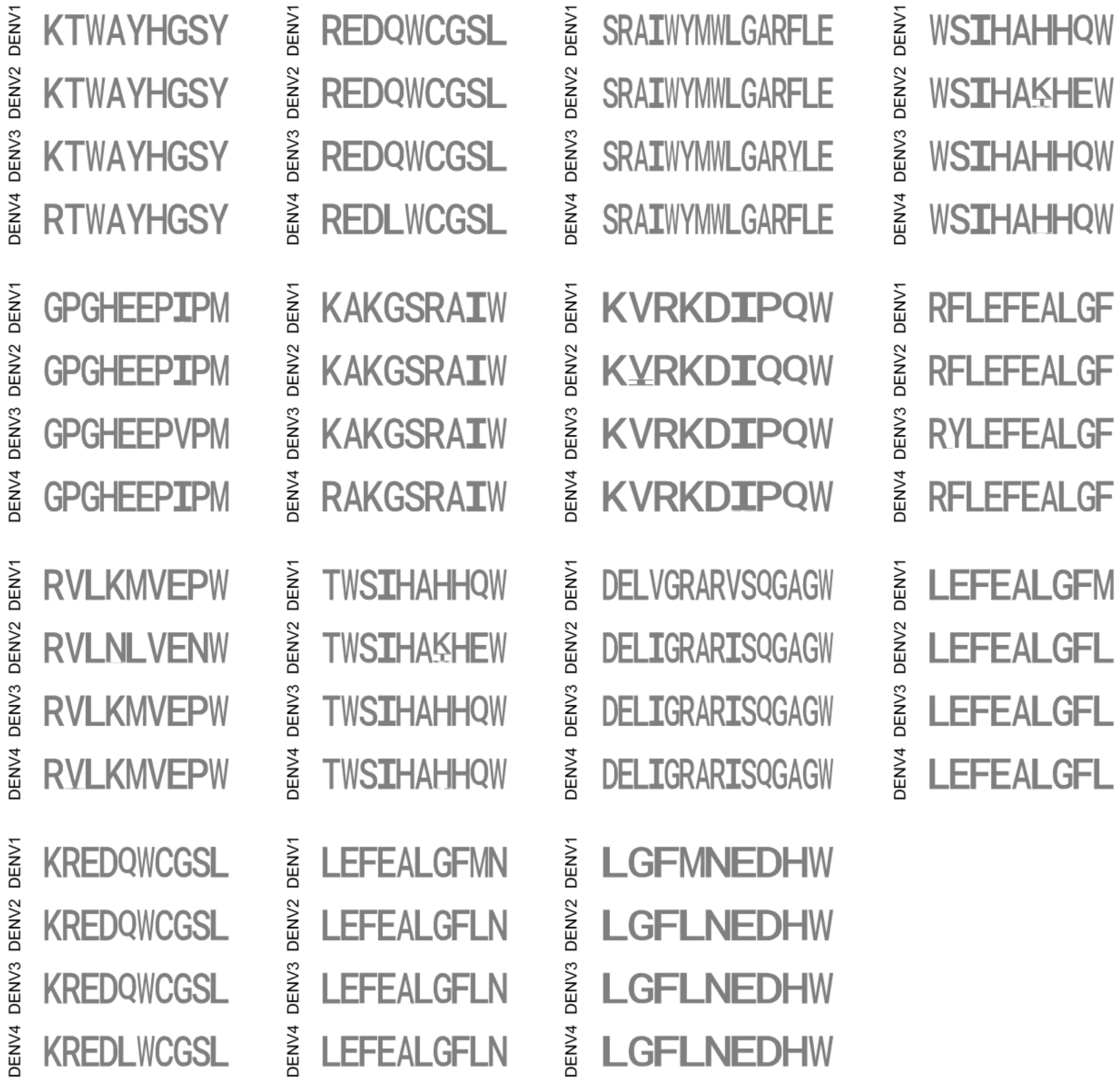

B

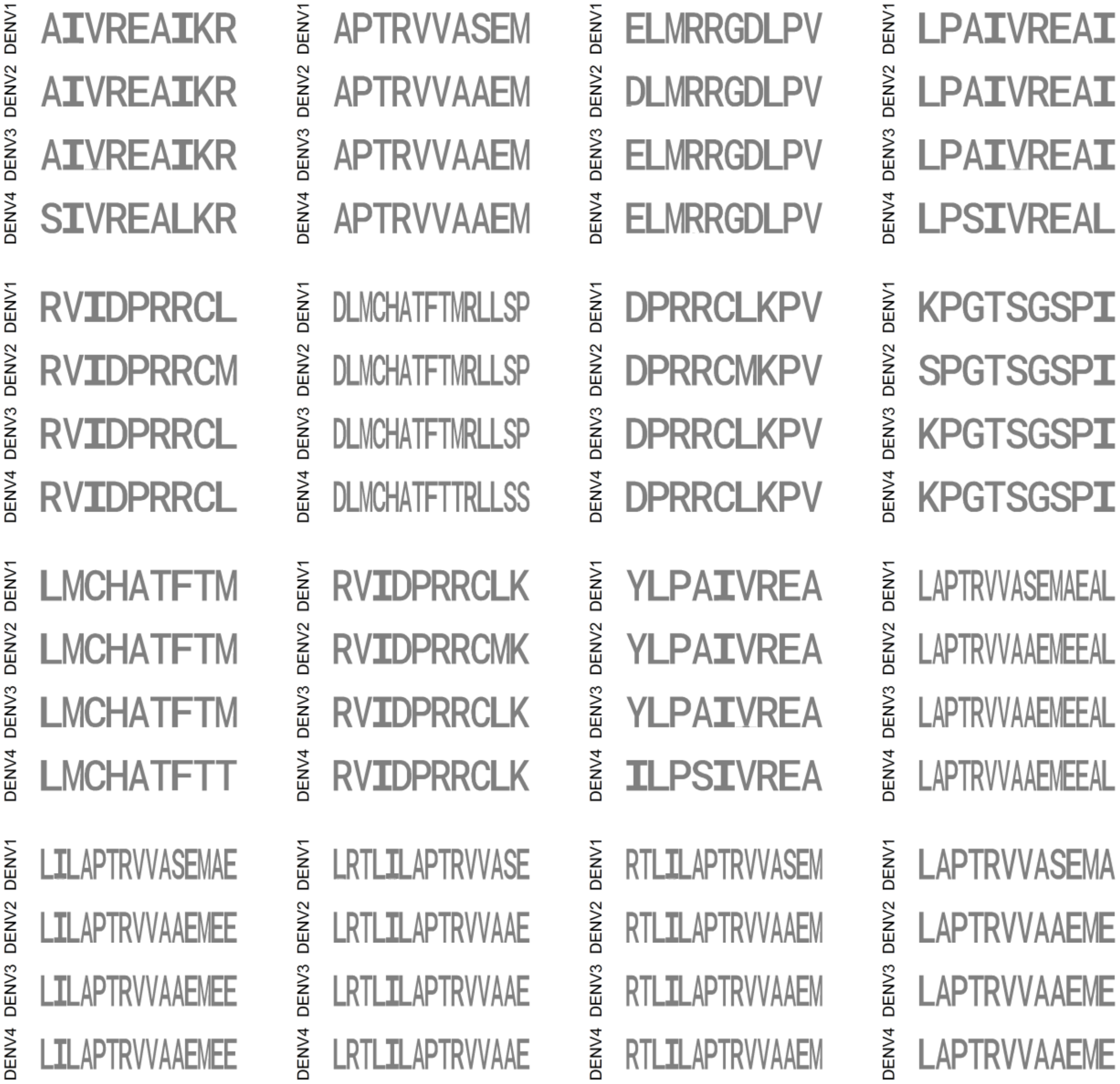
DENV1 AIVREAIKR
DENV2 AIVREAIKR
DENV3 AIVREAIKR
DENV4 SIVREALKR
DENV1 APTRVVASEM
DENV2 APTRVVAAEM
DENV3 APTRVVAAEM
DENV4 APTRVVAAEM
DENV1 ELMRRGDLPV
DENV2 DLMRRGDLPV
DENV3 ELMRRGDLPV
DENV4 ELMRRGDLPV
DENV1 LPAIVREAI
DENV2 LPAIVREAI
DENV3 LPAIVREAI
DENV4 LPSIVREAL
DENV1 RVIDPRRCL
DENV2 RVIDPRRCM
DENV3 RVIDPRRCL
DENV4 RVIDPRRCL
DENV1 DLMCHATFTMRLLSP
DENV2 DLMCHATFTMRLLSP
DENV3 DLMCHATFTMRLLSP
DENV4 DLMCHATFTTRLLSS
DENV1 DPRRCLKPV
DENV2 DPRRCMKPV
DENV3 DPRRCLKPV
DENV4 DPRRCLKPV
DENV1 KPGTSGSPI
DENV2 SPGTSGSPI
DENV3 KPGTSGSPI
DENV4 KPGTSGSPI
DENV1 LMCHATFTM
DENV2 LMCHATFTM
DENV3 LMCHATFTM
DENV4 LMCHATFTT
DENV1 RVIDPRRCLK
DENV2 RVIDPRRCMK
DENV3 RVIDPRRCLK
DENV4 RVIDPRRCLK
DENV1 YLPAIVREA
DENV2 YLPAIVREA
DENV3 YLPAIVREA
DENV4 ILPSIVREA
DENV1 LAPTRVVASEMAEAL
DENV2 LAPTRVVAAEMEEAL
DENV3 LAPTRVVAAEMEEAL
DENV4 LAPTRVVAAEMEEAL
DENV1 LILAPTRVVASEMAE
DENV2 LILAPTRVVAAEMEE
DENV3 LILAPTRVVAAEMEE
DENV4 LILAPTRVVAAEMEE
DENV1 LRTLILAPTRVVASE
DENV2 LRTLILAPTRVVAAE
DENV3 LRTLILAPTRVVAAE
DENV4 LRTLILAPTRVVAAE
DENV1 RTLILAPTRVVASEM
DENV2 RTLILAPTRVVAAEM
DENV3 RTLILAPTRVVAAEM
DENV4 RTLILAPTRVVAAEM
DENV1 LAPTRVVASEMA
DENV2 LAPTRVVAAEME
DENV3 LAPTRVVAAEME
DENV4 LAPTRVVAAEME

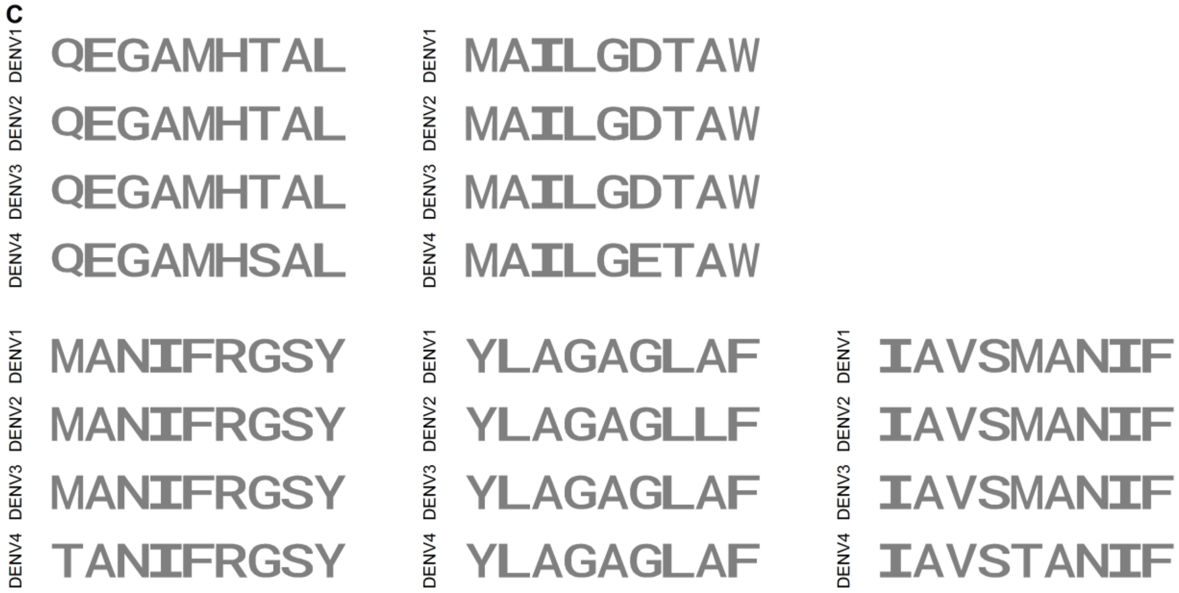

S2 Fig. Top conserved epitopes are different only at DENV serotype-specific residues. Sequence-logos of top epitopes that are not highly conserved in one of the four DENV serotypes for: **(A)** 15 epitopes of NS5, **(B)** 16 epitopes of NS3, and **(C)** 2 epitopes of E (top panel) and 3 epitopes of NS4b (bottom panel).
