# Supplementary material for: Cross-serotypically conserved epitope recommendations for a universal T cell-based dengue vaccine
Source: PLoS Negl Trop Dis. 2020 Sep 21;14(9):e0008676. doi: 10.1371/journal.pntd.0008676 (PMC7529213; doi:10.1371/journal.pntd.0008676)
Supplement: S3 Fig — Cells adjacent to each epitope represents its conservation within each DENV serotype. The conservation level (i.e., fraction of sequences in which a given epitope was exactly mapped) for an epitope within a serotype was determined by mapping it onto all the corresponding protein sequences for that serotype, as shown in Fig 2B. All epitopes are shown here in descending order (top to bottom then left to right) of their mean conservation across the serotypes and colored according to the protein from which they are derived. (PDF) [file pntd.0008676.s003.pdf]

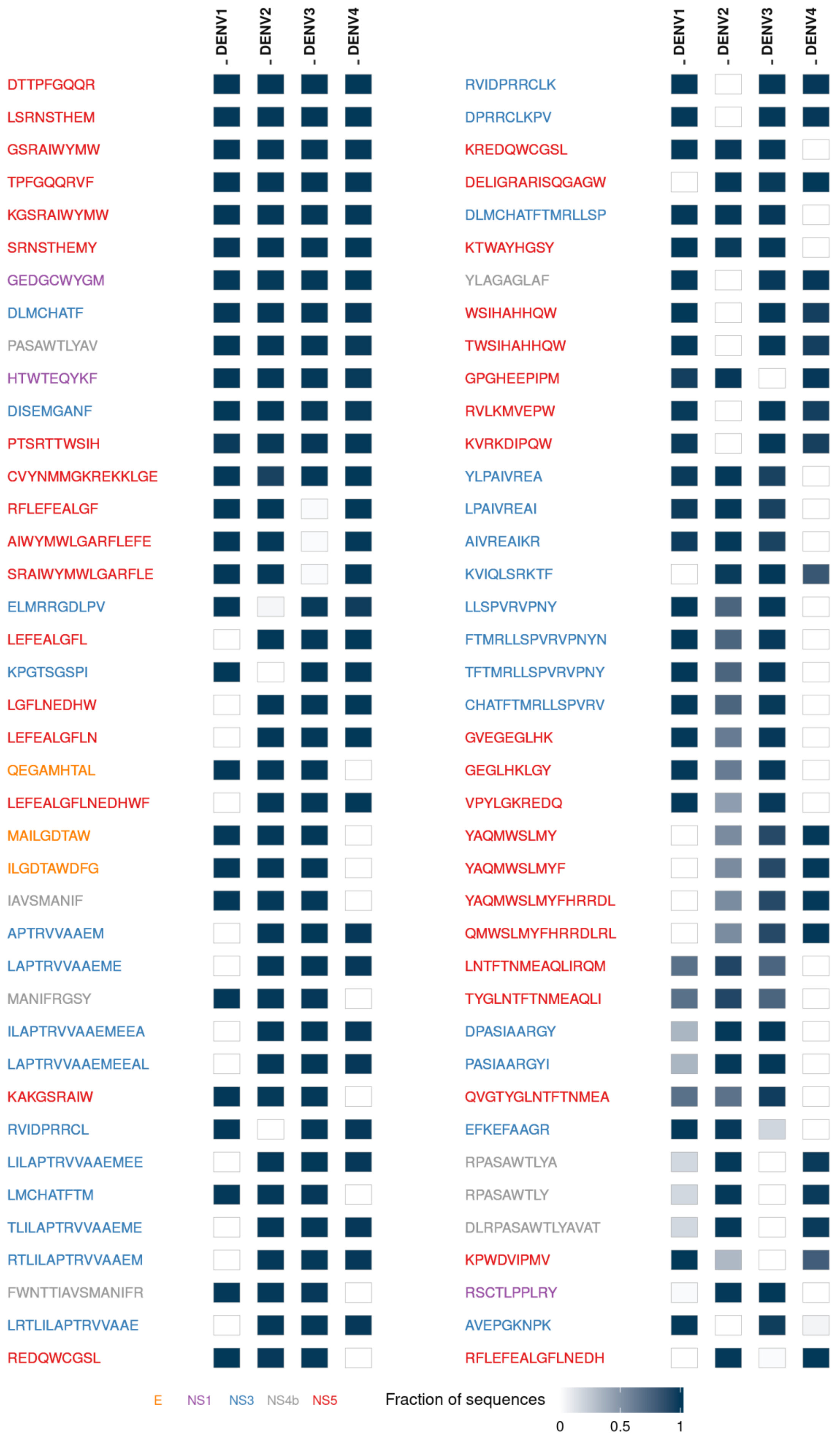
DENV1
DENV2
DENV3
DENV4
DTTPFGQQR
LSRNSTHEM
GSRAIWYMW
TPFGQQRVF
KGSRAIWYMW
SRNSTHEMY
GEDGCWYGM
DLMCHATF
PASAWTLYAV
HTWTEQYKF
DISEMGANF
PTSRTTWSIH
CVYNMMGKREKKLGE
RFLEFEALGF
AIWYMWLGARFLEFE
SRAIWYMWLGARFLE
ELMRRGDLPV
LEFEALGFL
KPGTSGSPI
LGFLNEDHW
LEFEALGFLN
QEGAMHTAL
LEFEALGFLNEDHWF
MAILGDTAW
ILGDTAWDFG
IAVSMANIF
APTRVVAAEM
LAPTRVVAAEME
MANIFRGSY
ILAPTRVVAAEMEEA
LAPTRVVAAEMEEAL
KAKGSRAIW
RVIDPRRCL
LILAPTRVVAAEMEE
LMCHATFTM
TLILAPTRVVAAEME
RTLILAPTRVVAAEM
FWNTTIAVSMANIFR
LRTLILAPTRVVAAE
REDQWCGSL
RVIDPRRCLK
DPRRCLKPV
KREDQWCGSL
DELIGRARISQGAGW
DLMCHATFTMRLLSP
KTWAYHGSY
YLAGAGLAF
WSIHAHHQW
TWSIHAHHQW
GPGHEEPIPM
RVLKMVEPW
KVRKDIPQW
YLPAIVREA
LPAIVREAI
AIVREAIKR
KVIQLSRKTF
LLSPVRVPNY
FTMRLLSPVRVPNYN
TFTMRLLSPVRVPNY
CHATFTMRLLSPVRV
GVEGEGLHK
GEGLHKLGY
VPYLGKREDQ
YAQMWSLMY
YAQMWSLMYF
YAQMWSLMYFHRRDL
QMWSLMYFHRRDLRL
LNTFTNMEAQLIRQM
TYGLNTFTNMEAQLI
DPASIAARGY
PASIAARGYI
QVGTYGLNTFTNMEA
EFKEFAAGR
RPASAWTLYA
RPASAWTLY
DLRPASAWTLYAVAT
KPWDVIPMV
RSCTLPPLRY
AVEPGKNPK
RFLEFEALGFLNEDH
E
NS1
NS3
NS4b
NS5
Fraction of sequences
0
0.5
1

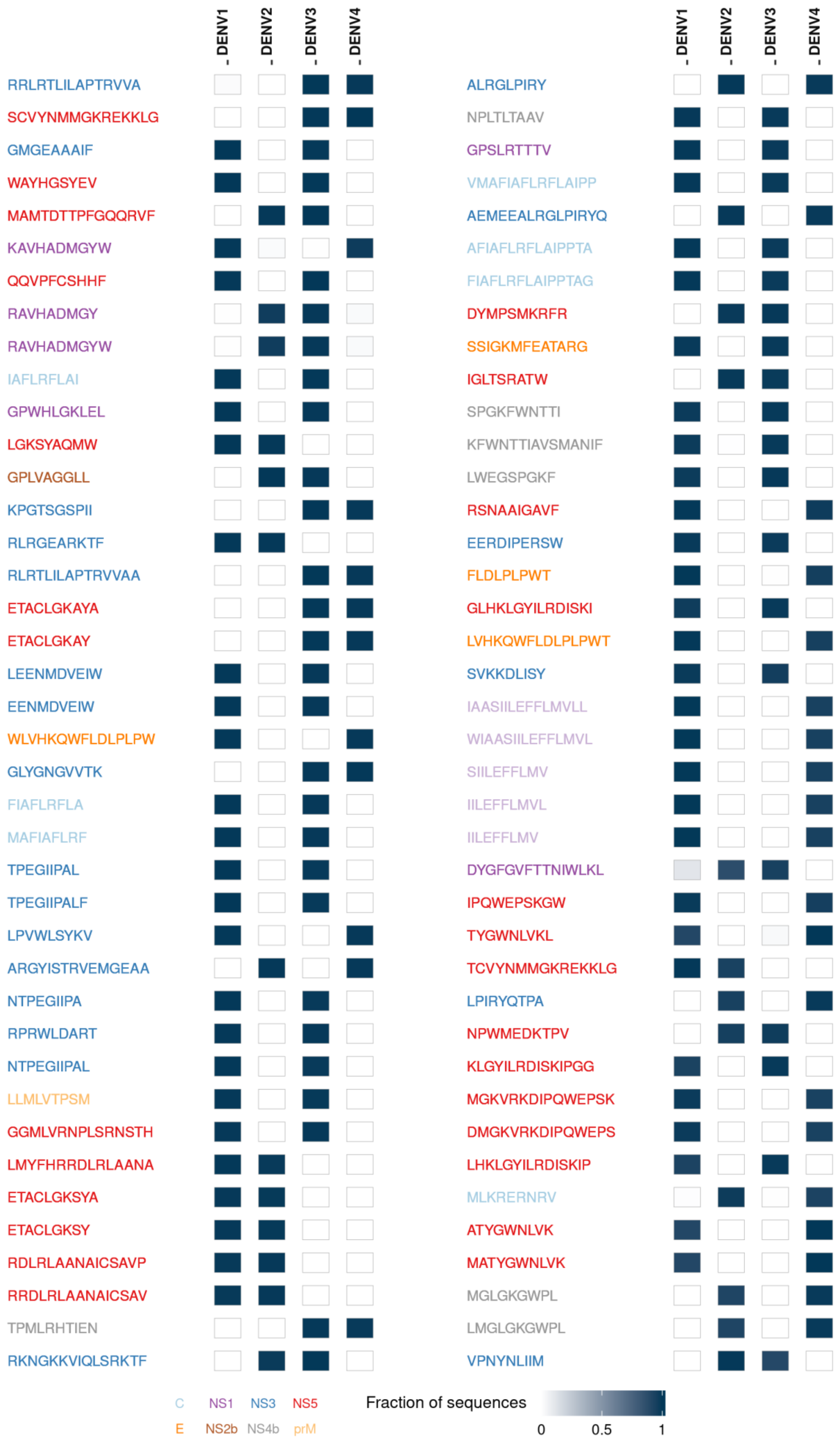

_DENV1
_DENV2
_DENV3
_DENV4
RRLRTLILAPTRVVA
SCVYNMMGKREKKLG
GMGEAAAIF
WAYHGSYEV
MAMTDTTPFGQQRVF
KAVHADMGYW
QQVPFCSHHF
RAVHADMGY
RAVHADMGYW
IAFLRFLAI
GPWHLGKLEL
LGKSYAQMW
GPLVAGGLL
KPGTSGSPII
RLRGEARKTF
RLRTLILAPTRVVAA
ETACLGKAYA
ETACLGKAY
LEENMDVEIW
EENMDVEIW
WLVHKQWFLDLPLPW
GLYGNGVVTK
FIAFLRFLA
MAFIAFLRF
TPEGIIPAL
TPEGIIPALF
LPVWLSYKV
ARGYISTRVEMGEAA
NTPEGIIPA
RPRWLDART
NTPEGIIPAL
LLMLVTPSM
GGMLVRNPLSRNSTH
LMYFHRRDLRLAANA
ETACLGKSYA
ETACLGKSY
RDLRLAANAICSAVP
RRDLRLAANAICSAV
TPMLRHTIEN
RKNGKKVIQLSRKTF
ALRGLPIRY
NPLTLTAAV
GPSLRTTTV
VMAFIAFLRFLAIPP
AEMEEALRGLPIRYQ
AFIAFLRFLAIPPTA
FIAFLRFLAIPPTAG
DYMPSMKRFR
SSIGKMFEATARG
IGLTSRATW
SPGKFWNTTI
KFWNTTIAVSMANIF
LWEGSPGKF
RSNAAIGAVF
EERDIPERSW
FLDLPLPWT
GLHKLGYILRDISKI
LVHKQWFLDLPLPWT
SVKKDLISY
IAASIILEFFLMVLL
WIAASIILEFFLMVL
SIILEFFLMV
IILEFFLMVL
IILEFFLMV
DYGFGVFTTNIWLKL
IPQWEPSKGW
TYGWNLVKL
TCVYNMMGKREKKLG
LPIRYQTPA
NPWMEDKTPV
KLGYILRDISKIPGG
MGKVRKDIPQWEPSK
DMGKVRKDIPQWEPS
LHKLGYILRDISKIP
MLKRERNRV
ATYGWNLVK
MATYGWNLVK
MGLGKGWPL
LMGLGKGWPL
VPNYNLIIM
C
NS1
NS3
NS5
E
NS2b
NS4b
prM
Fraction of sequences
0
0.5
1

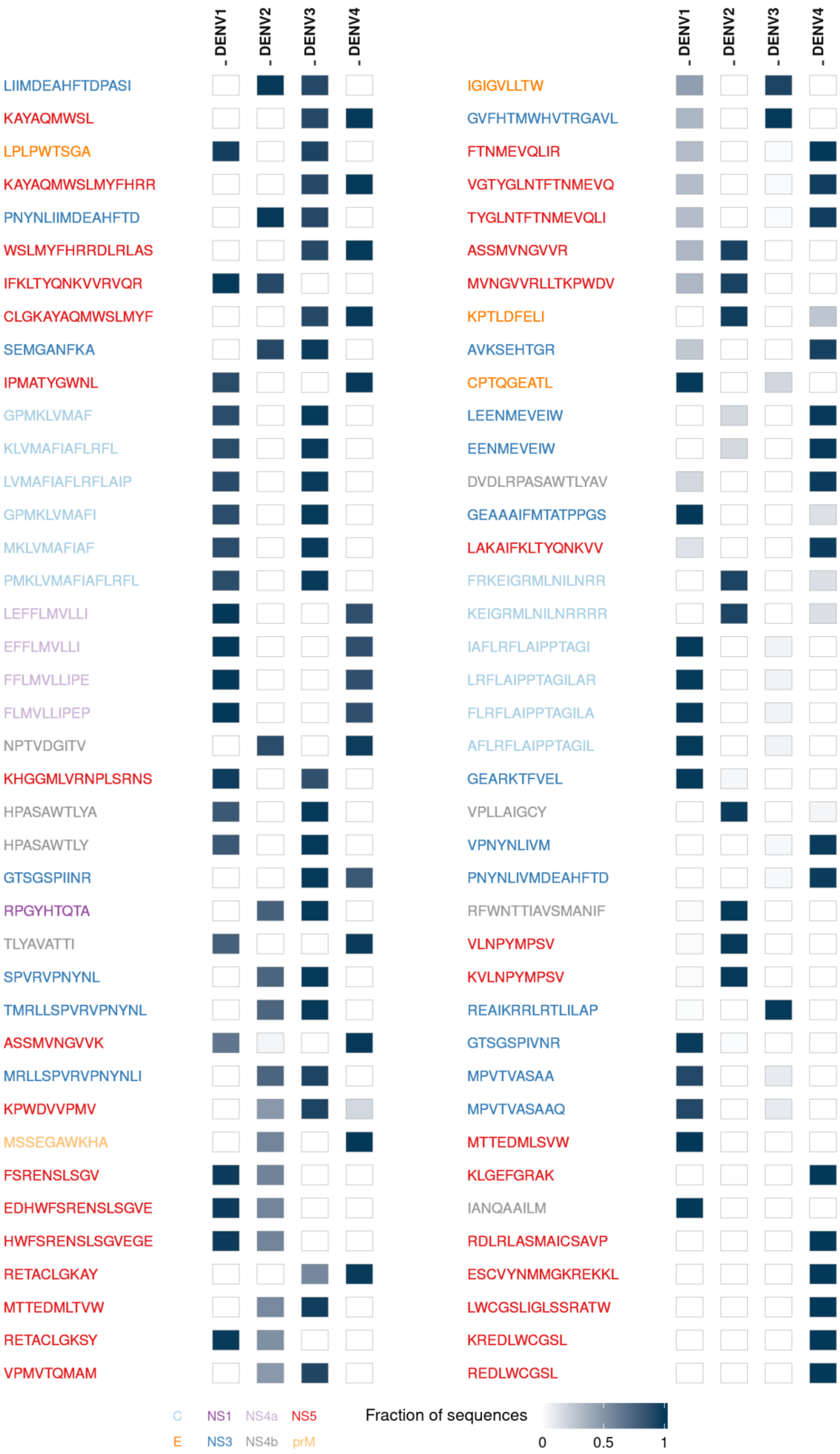
DENV1
DENV2
DENV3
DENV4
LIIMDEAHFTDPASI
KAYAQMWSL
LPLPWTSGA
KAYAQMWSLMYFHRR
PNYNLIIMDEAHFTD
WSLMYFHRRDLRLAS
IFKLTYQNKVVRVQR
CLGKAYAQMWSLMYF
SEMGANFKA
IPMATYGWNL
GPMKLVMAF
KLVMAFIAFLRFL
LVMAFIAFLRFLAIP
GPMKLVMAFI
MKLVMAFIAF
PMKLVMAFIAFLRFL
LEFFLMVLLI
EFFLMVLLI
FFLMVLLIPE
FLMVLLIPEP
NPTVDGITV
KHGGMLVRNPLSRNS
HPASAWTLYA
HPASAWTLY
GTSGSPIINR
RPGYHTQTA
TLYAVATTI
SPVRVPNYNL
TMRLLSPVRVPNYNL
ASSMVNGVVK
MRLLSPVRVPNYNLI
KPWDVVPMV
MSSEGAWKHA
FSRENSLSGV
EDHWFSRENSLSGVE
HWFSRENSLSGVEGE
RETACLGKAY
MTTEDMLTVW
RETACLGKSY
VPMVTQMAM
IGIGVLLTW
GVFHTMWHVTRGAVL
FTNMEVQLIR
VGTYGLNTFTNMEVQ
TYGLNTFTNMEVQLI
ASSMVNGVVR
MVNGVVRLLTKPWDV
KPTLDFELI
AVKSEHTGR
CPTQGEATL
LEENMEVEIW
EENMEVEIW
DVDLRPASAWTLYAV
GEAAAIFMTATPPGS
LAKAIFKLTYQNKVV
FRKEIGRMLNILNRR
KEIGRMLNILNRRRR
IAFLRFLAIPPTAGI
LRFLAIPPTAGILAR
FLRFLAIPPTAGILA
AFLRFLAIPPTAGIL
GEARKTFVEL
VPLLAIGCY
VPNYNLIVM
PNYNLIVMDEAHFTD
RFWNTTIAVSMANIF
VLNPYMPSV
KVLNPYMPSV
REAIKRRLRTLILAP
GTSGSPIVNR
MPVTVASAA
MPVTVASAAQ
MTTEDMLSVW
KLGEFGRAK
IANQAAILM
RDLRLASMAICSAVP
ESCVYNMMGKREKKL
LWCGSLIGLSSRATW
KREDLWCGSL
REDLWCGSL
C
NS1
NS4a
NS5
E
NS3
NS4b
prM
Fraction of sequences
0
0.5
1

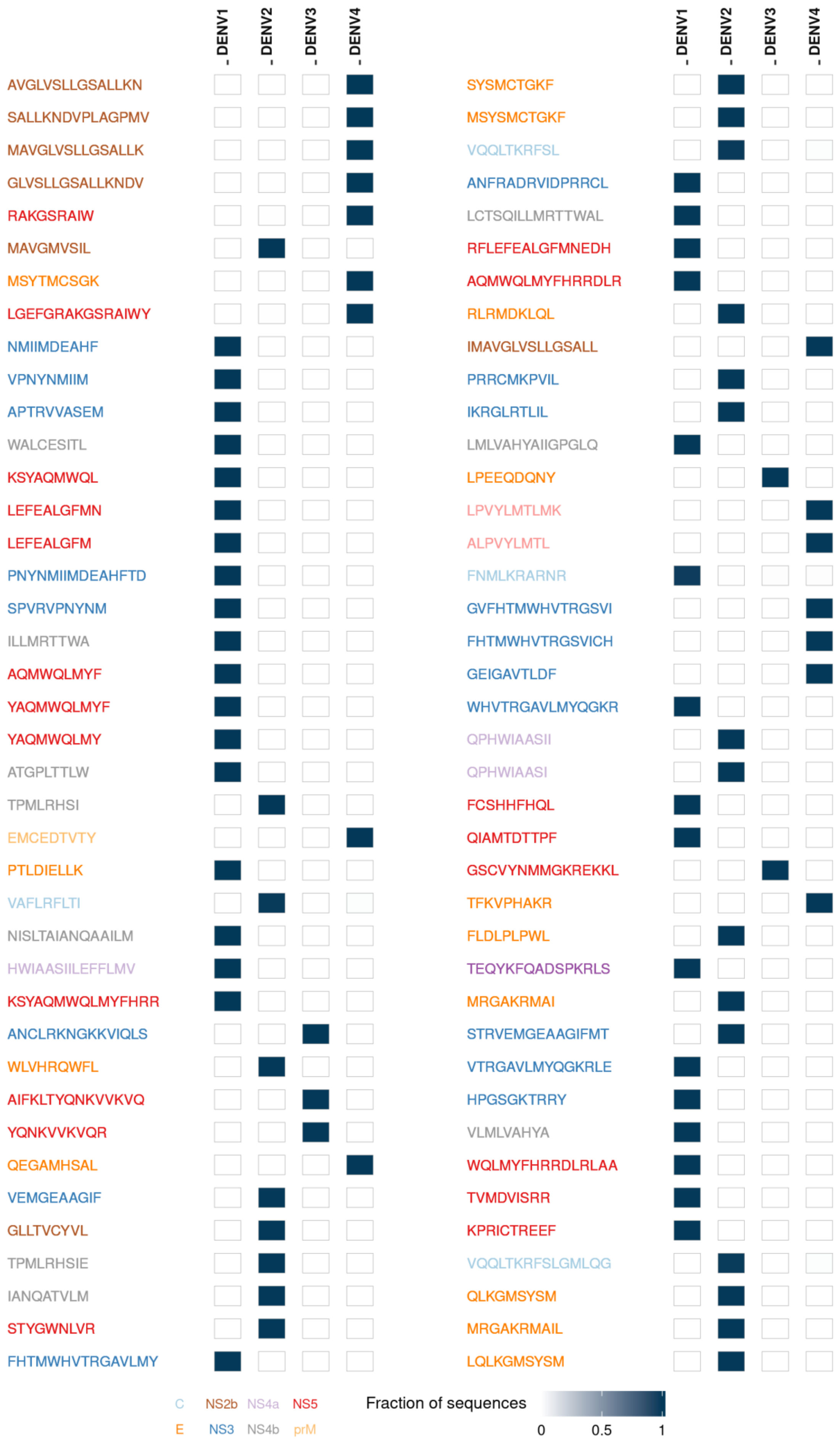
DENV1
DENV2
DENV3
DENV4
AVGLVSLLGSALLKN
SALLKNDVPLAGPMV
MAVGLVSLLGSALLK
GLVSLLGSALLKNDV
RAKGSRAIW
MAVGMVSIL
MSYTMCSGK
LGEFGRAKGSRAIWY
NMIIMDEAHF
VPNYNMIIM
APTRVVASEM
WALCESITL
KSYAQMWQL
LEFEALGFMN
LEFEALGFM
PNYNMIIMDEAHFTD
SPVRVPNYNM
ILLMRTTWA
AQMWQLMYF
YAQMWQLMYF
YAQMWQLMY
ATGPLTTLW
TPMLRHSI
EMCEDTVTY
PTLDIELLK
VAFLRFLTI
NISLTAIANQAAILM
HWIAASIILEFFLMV
KSYAQMWQLMYFHRR
ANCLRKNGKKVIQLS
WLVHRQWFL
AIFKLTYQNKVVKVQ
YQNKVVKVQR
QEGAMHSAL
VEMGEAAGIF
GLLTVCYVL
TPMLRHSIE
IANQATVLM
STYGWNLVR
FHTMWHVTRGAVLMY
SYSMCTGKF
MSYSMCTGKF
VQQLTKRFSL
ANFRADRVIDPRRCL
LCTSQILLMRTTWAL
RFLEFEALGFMNEDH
AQMWQLMYFHRRDLR
RLRMDKLQL
IMAVGLVSLLGSALL
PRRCMKPVIL
IKRGLRTLIL
LMLVAHYAIIGPGLQ
LPEEQDQNY
LPVYLMTLMK
ALPVYLMTL
FNMLKRARNR
GVFHTMWHVTRGSVI
FHTMWHVTRGSVICH
GEIGAVTLDF
WHVTRGAVLMYQGKR
QPHWIAASII
QPHWIAASI
FCSHHFHQL
QIAMTDTTPF
GSCVYNMMGKREKKL
TFKVPHAKR
FLDLPLPWL
TEQYKFQADSPKRLS
MRGAKRMAI
STRVEMGEAAGIFMT
VTRGAVLMYQGKRLE
HPGSGKTRRY
VLMLVAHYA
WQLMYFHRRDLRLAA
TVMDVISRR
KPRICTREEF
VQQLTKRFSLGMLQG
QLKGMSYSM
MRGAKRMAIL
LQLKGMSYSM
C
NS2b
NS4a
NS5
E
NS3
NS4b
prM
Fraction of sequences
0
0.5
1

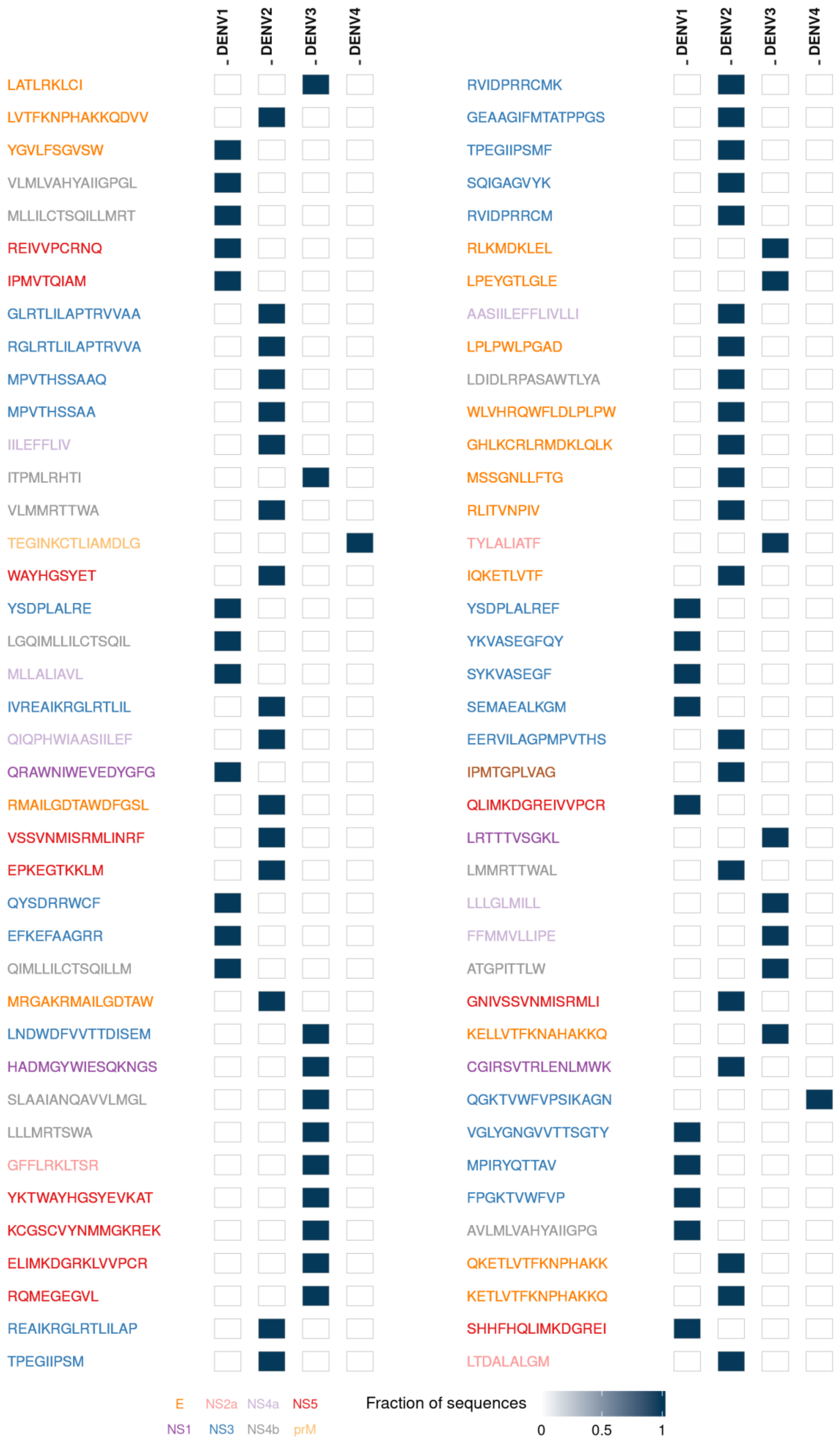

DENV1
DENV2
DENV3
DENV4
LATLRKLCI
LVTFKNPHAKKQDVV
YGVLFSGVSW
VLMLVAHYAIIGPGL
MLLILCTSQILLMRT
REIVVPCRNQ
IPMVTQIAM
GLRTLILAPTRVVAA
RGLRTLILAPTRVVA
MPVTHSSAAQ
MPVTHSSAA
IILEFFLIV
ITPMLRHTI
VLMMRTTWA
TEGINKCTLIAMDLG
WAYHGSYET
YSDPLALRE
LGQIMLLILCTSQIL
MLLALIAVL
IVREAIKRGLRTLIL
QIQPHWIAASIILEF
QRAWNIWEVEDYGFG
RMAILGDTAWDFGSL
VSSVNMISRMLINRF
EPKEGTKKLM
QYSDRRWCF
EFKEFAAGRR
QIMLLILCTSQILLM
MRGAKRMAILGDTAW
LNDWDFVVTTDISEM
HADMGYWIESQKNGS
SLAAIANQAVVLMGL
LLLMRTSWA
GFFLRKLTSR
YKTWAYHGSYEVKAT
KCGSCVYNMMGKREK
ELIMKDGRKLVVPCR
RQMEGEGVL
REAIKRGLRTLILAP
TPEGIIPSM
RVIDPRRCMK
GEAAGIFMTATPPGS
TPEGIIPSMF
SQIGAGVYK
RVIDPRRCM
RLKMDKLEL
LPEYGTLGLE
AASIILEFFLIVLLI
LPLPWLPGAD
LDIDLRPASAWTLYA
WLVHRQWFLDLPLPW
GHLKCRLRMDKLQLK
MSSGNLLFTG
RLITVNPIV
TYLALIATF
IQKETLVTF
YSDPLALREF
YKVASEGFQY
SYKVASEGF
SEMAEALKGM
EERVILAGPMPVTHS
IPMTGPLVAG
QLIMKDGREIVVPCR
LRTTTVSGKL
LMMRTTWAL
LLLGLMILL
FFMMVLLIPE
ATGPITTLW
GNIVSSVNMISRMLI
KELLVTFKNAHAKKQ
CGIRSVTRLENLMWK
QGKTVWFVPSIKAGN
VGLYGNGVVTTSGTY
MPIRYQTTAV
FPGKTVWFVP
AVLMLVAHYAIIGPG
QKETLVTFKNPHAKK
KETLVTFKNPHAKKQ
SHHFHQLIMKDGREI
LTDALALGM
E
NS2a
NS4a
NS5
NS1
NS3
NS4b
prM
Fraction of sequences
0
0.5
1

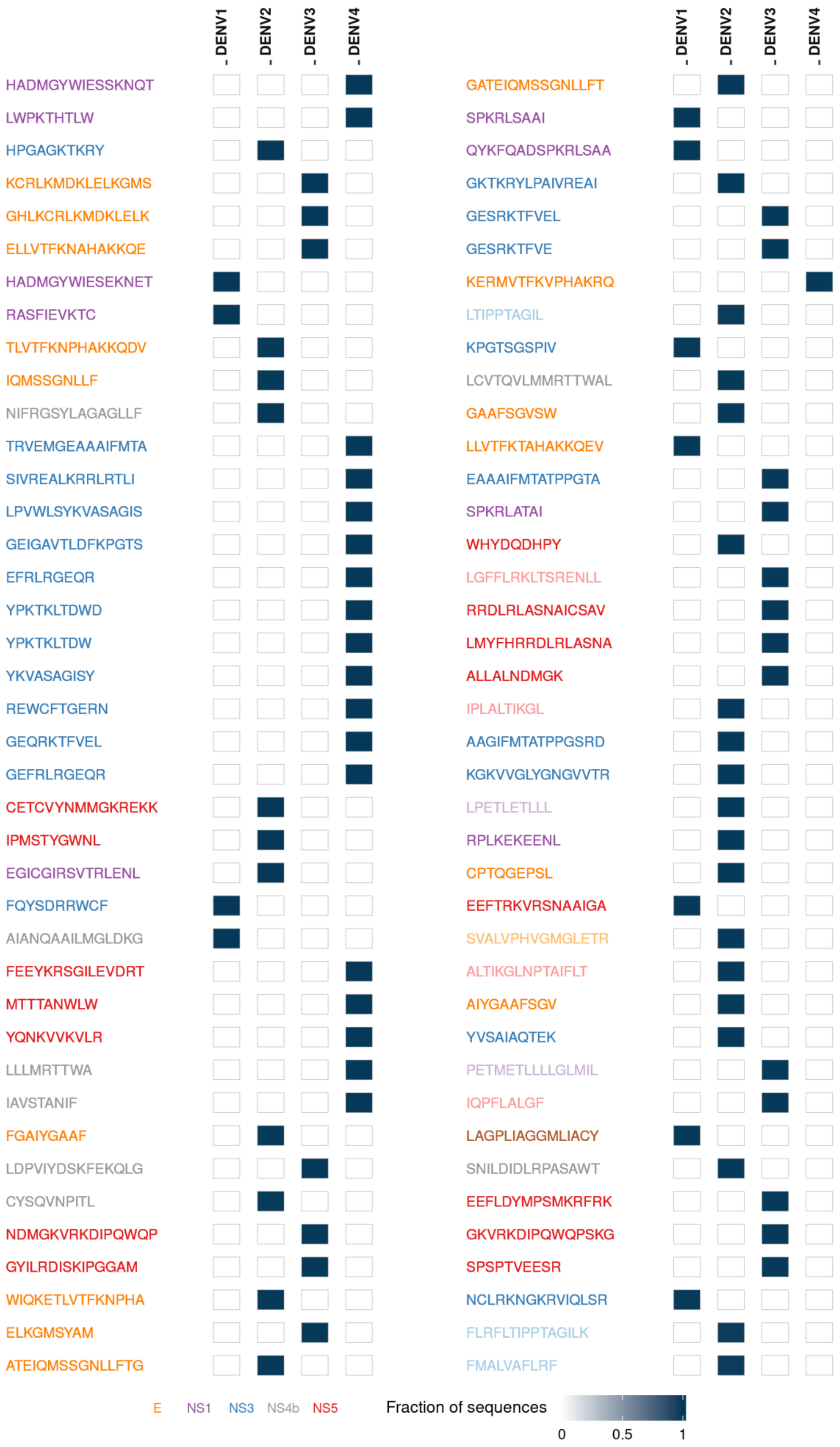
DENV1
DENV2
DENV3
DENV4
HADMGYWIESSKNQT
LWPKTHTLW
HPGAGKTKRY
KCRLKMDKLELKGMS
GHLKCRLKMDKLELK
ELLVTFKNAHAKKQE
HADMGYWIESEKNET
RASFIEVKTC
TLVTFKNPHAKKQDV
IQMSSGNLLF
NIFRGSYLAGAGLLF
TRVEMGEAAAIFMTA
SIVREALKRRLRTLI
LPVWLSYKVASAGIS
GEIGAVTLDFKPGTS
EFRLRGEQR
YPKTKLTDWD
YPKTKLTDW
YKVASAGISY
REWCFTGERN
GEQRKTFVEL
GEFRLRGEQR
CETCVYNMMGKREKK
IPMSTYGWNL
EGICGIRSVTRLENL
FQYSDRRWCF
AIANQAAILMGLDKG
FEEYKRSGILEVDRT
MTTTANWLW
YQNKVVKVLR
LLLMRTTWA
IAVSTANIF
FGAIYGAAF
LDPVIYDSKFEKQLG
CYSQVNPITL
NDMGKVRKDIPQWQP
GYILRDISKIPGGAM
WIQKETLVTFKNPHA
ELKGMSYAM
ATEIQMSSGNLLFTG
GATEIQMSSGNLLFT
SPKRLSAAI
QYKFQADSPKRLSAA
GKTKRYLPAIVREAI
GESRKTFVEL
GESRKTFVE
KERMVTFKVPHAKRQ
LTIPPTAGIL
KPGTSGSPIV
LCVTQVLMMRTTWAL
GAAFSGVSW
LLVTFKTAHAKKQEV
EAAAIFMTATPPGTA
SPKRLATAI
WHYDQDHPY
LGFFLRKLTSRENLL
RRDLRLASNAICSAV
LMYFHRRDLRLASNA
ALLALNDMGK
IPLALTIKGL
AAGIFMTATPPGSRD
KGKVVGLYGNGVVTR
LPETLETLLL
RPLKEKEENL
CPTQGEPSL
EEFTRKVRSNAAIGA
SVALVPHVGMGLETR
ALTIKGLNPTAIFLT
AIYGAAFSGV
YVSAIAQTEK
PETMETLLLLGLMIL
IQPFLALGF
LAGPLIAGGMLIACY
SNILDIDLRPASAWT
EEFLDYMPSMKRFRK
GKVRKDIPQWQPSKG
SPSPTVEESR
NCLRKNGKRVIQLSR
FLRFLTIPPTAGILK
FMALVAFLRF
E
NS1
NS3
NS4b
NS5
Fraction of sequences
0
0.5
1

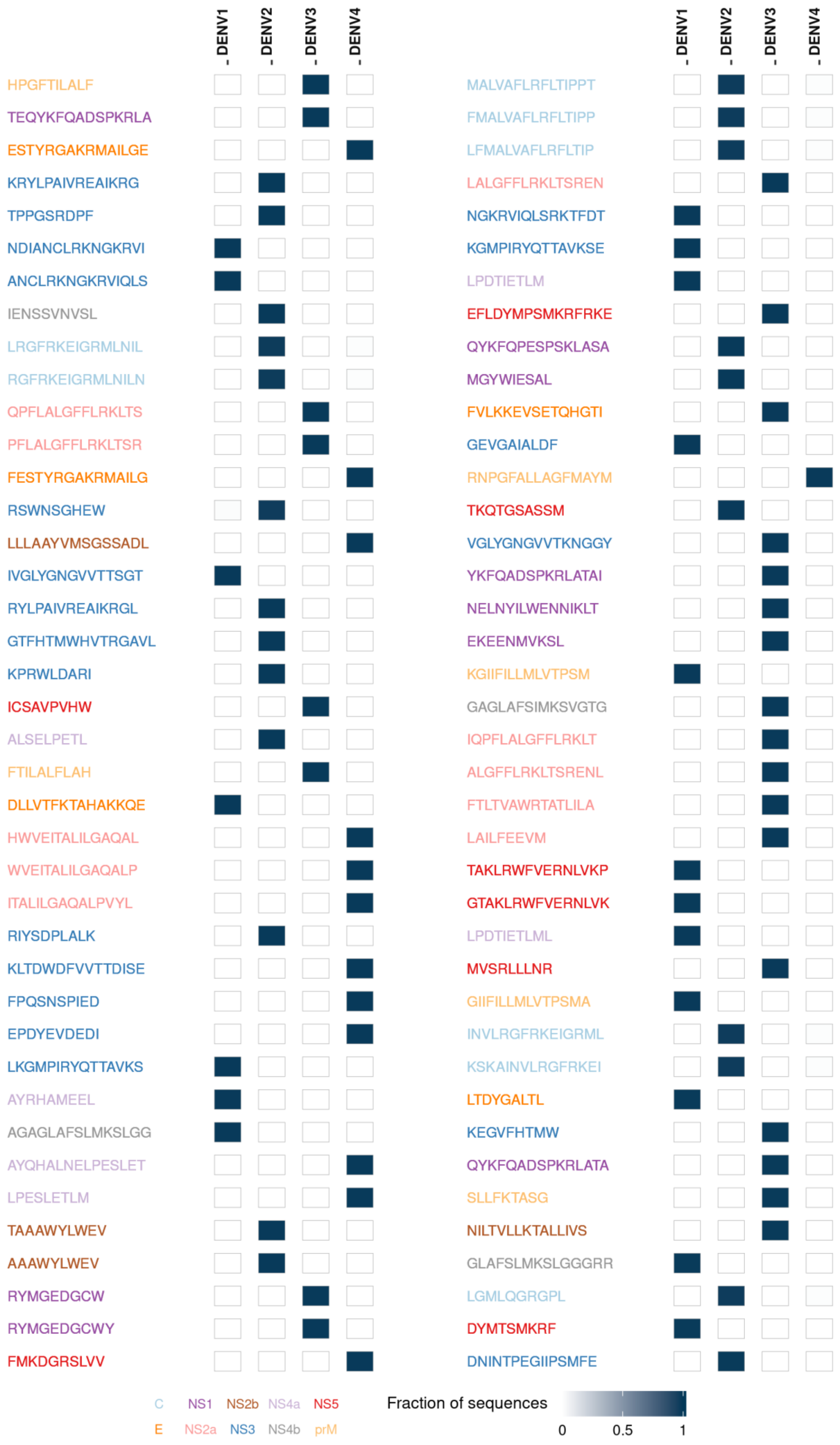

DENV1
DENV2
DENV3
DENV4
HPGFTILALF
TEQYKFQADSPKRLA
ESTYRGAKRMAILGE
KRYLPAIVREAIKRG
TPPGSRDPF
NDIANCLRKNGKRVI
ANCLRKNGKRVIQLS
IENSSVNVSL
LRGFRKEIGRMLNIL
RGFRKEIGRMLNILN
QPFLALGFFLRKLTS
PFLALGFFLRKLTSR
FESTYRGAKRMAILG
RSWNSGHEW
LLLAAYVMSGSSADL
IVGLYGNGVVTTSGT
RYLPAIVREAIKRGL
GTFHTMWHVTRGAVL
KPRWLDARI
ICSAVPVHW
ALSELPETL
FTILALFLAH
DLLVTFKTAHAKKQE
HWVEITALILGAQAL
WVEITALILGAQALP
ITALILGAQALPVYL
RIYSDPLALK
KLTDWDFVVTTDISE
FPQSNSPIED
EPDYEVDEDI
LKGMPIRYQTTAVKS
AYRHAMEEL
AGAGLAFSLMKSLGG
AYQHALNELPESLET
LPESLETLM
TAAAWYLWEV
AAAWYLWEV
RYMGEDGCW
RYMGEDGCWY
FMKDGRSLVV
MALVAFLRFLTIPPT
FMALVAFLRFLTIPP
LFMALVAFLRFLTIP
LALGFFLRKLTSREN
NGKRVIQLSRKTFDT
KGMPIRYQTTAVKSE
LPDTIETLM
EFLDYMPSMKRFRKE
QYKFQPESPSKLASA
MGYWIESAL
FVLKKEVSETQHGTI
GEVGAIALDF
RNPGFALLAGFMAYM
TKQTGSASSM
VGLYGNGVVTKNGGY
YKFQADSPKRLATAI
NELNYILWENNIKLT
EKEENMVKSL
KGIIFILLMLVTPSM
GAGLAFSIMKSVGTG
IQPFLALGFFLRKLT
ALGFFLRKLTSRENL
FTLTVAWRTATLILA
LAILFEEVM
TAKLRWFVERNLVKP
GTAKLRWFVERNLVK
LPDTIETLML
MVSRLLLNR
GIIFILLMLVTPSMA
INVLRGFRKEIGRML
KSKAINVLRGFRKEI
LTDYGALTL
KEGVFHTMW
QYKFQADSPKRLATA
SLLFKTASG
NILTVLLKTALLIVS
GLAFSLMKSLGGGRR
LGMLQGRGPL
DYMTSMKRF
DNINTPEGIIPSMFE
C
E
NS1
NS2a
NS2b
NS3
NS4a
NS4b
NS5
prM
Fraction of sequences
0
0.5
1

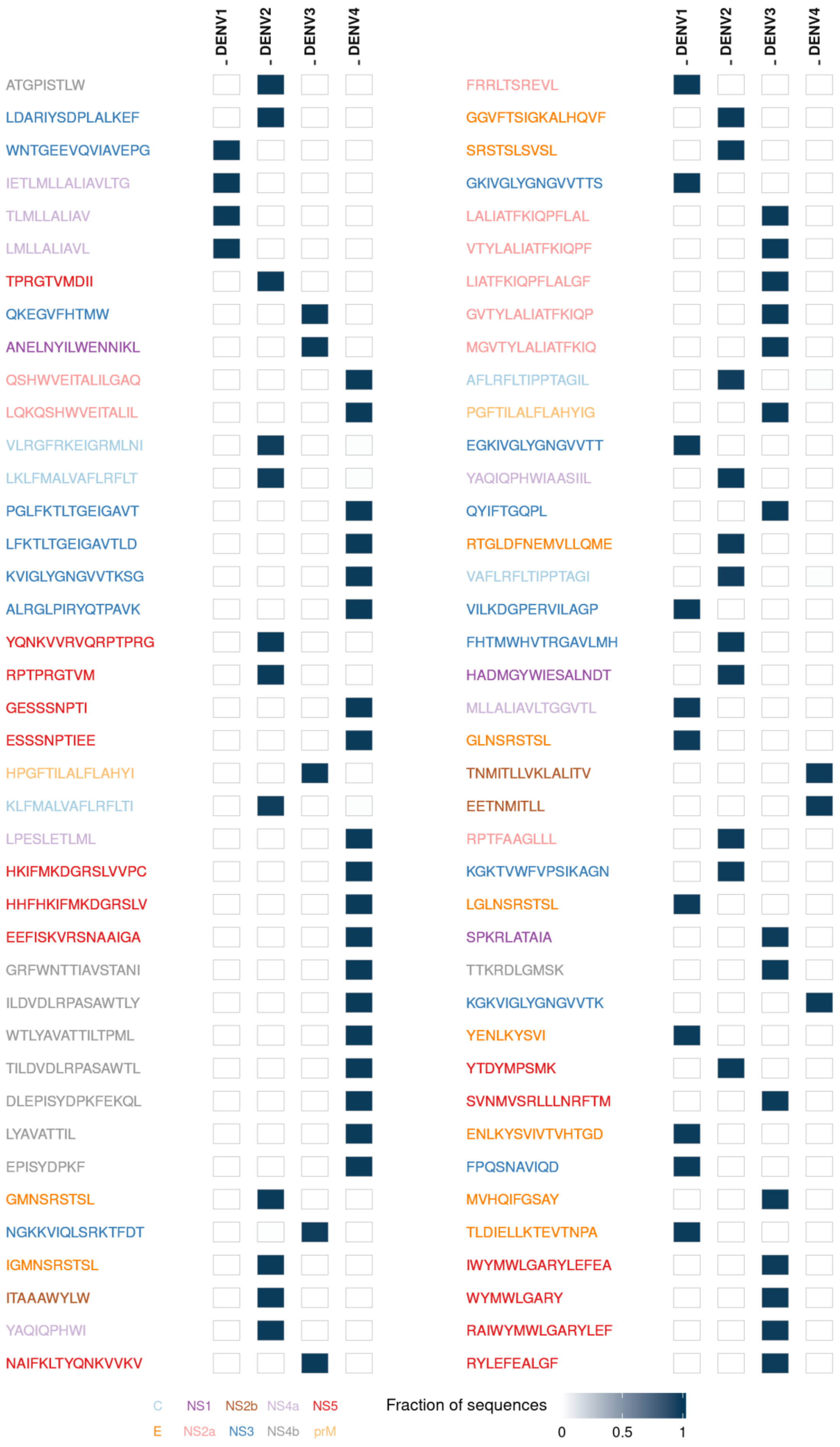
DENV1
DENV2
DENV3
DENV4
ATGPISTLW
LDARIYSDPLALKEF
WNTGEEVQVIAVEPG
IETLMLLALIAVLTG
TLMLLALIAV
LMLLALIAVL
TPRGTVMDII
QKEGVFHTMW
ANELNYILWENNIKL
QSHWVEITALILGAQ
LQKQSHWVEITALIL
VLRGFRKEIGRMLNI
LKLFMALVAFLRFLT
PGLFKTLTGEIGAVT
LFKTLTGEIGAVTLD
KVIGLYGNGVVTKSG
ALRGLPIRYQTPAVK
YQNKVVRVQRPTPRG
RPTPRGTVM
GESSSNPTI
ESSSNPTIEE
HPGFTILALFLAHYI
KLFMALVAFLRFLTI
LPESLETLML
HKIFMKDGRSLVVPC
HHFHKIFMKDGRSLV
EEFISKVRSNAAIGA
GRFWNTTIAVSTANI
ILDVDLRPASAWTLY
WTLYAVATTILTPML
TILDVDLRPASAWTL
DLEPISYDPKFEKQL
LYAVATTIL
EPISYDPKF
GMNSRSTSL
NGKKVIQLSRKTFDT
IGMNSRSTSL
ITAAAWYLW
YAQIQPHWI
NAIFKLTYQNKVVKV
FRRLTSREVL
GGVFTSIGKALHQVF
SRSTSLSVSL
GKIVGLYGNGVVTTS
LALIATFKIQPFLAL
VTYLALIATFKIQPF
LIATFKIQPFLALGF
GVTYLALIATFKIQP
MGVTYLALIATFKIQ
AFLRFLTIPPTAGIL
PGFTILALFLAHYIG
EGKIVGLYGNGVVTT
YAQIQPHWIAASIIL
QYIFTGQPL
RTGLDFNEMVLLQME
VAFLRFLTIPPTAGI
VILKDGPERVILAGP
FHTMWHVTRGAVLMH
HADMGYWIESALNDT
MLLALIAVLTGGVTL
GLNSRSTSL
TNMITLLVKLALITV
EETNMITLL
RPTFAAGLLL
KGKTVWFVPSIKAGN
LGLNSRSTSL
SPKRLATAIA
TTKRDLGMSK
KGKVIGLYGNGVVTK
YENLKYSVI
YTDYMPSMK
SVNMVSRLLLNRFTM
ENLKYSVIVTVHTGD
FPQSNAVIQD
MVHQIFGSAY
TLDIELLKTEVTNPA
IWYMWLGARYLEFEA
WYMWLGARY
RAIWYMWLGARYLEF
RYLEFEALGF
C
NS1
NS2b
NS4a
NS5
E
NS2a
NS3
NS4b
prM
Fraction of sequences
0
0.5
1

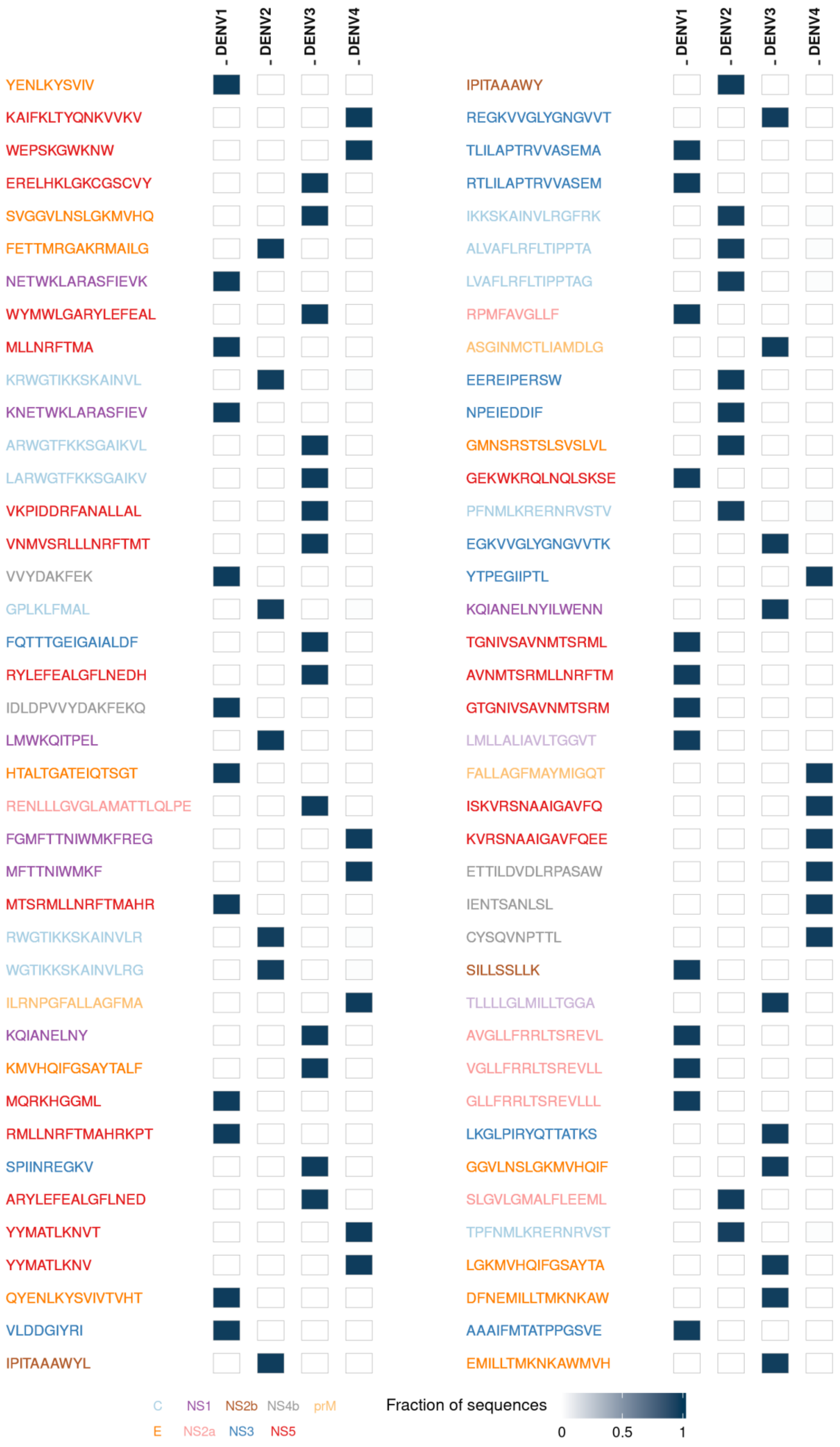

DENV1
DENV2
DENV3
DENV4
YENLKYSVIV
KAIFKLTYQNKVVKV
WEPSKGWKNW
ERELHKLGKCGSCVY
SVGGVLNSLGKMVHQ
FETTMRGAKRMAILG
NETWKLARASFIEVK
WYMWLGARYLEFEAL
MLLNRFTMA
KRWGTIKKSKAINVL
KNETWKLARASFIEV
ARWGTFKKSGAIKVL
LARWGTFKKSGAIKV
VKPIDDRFANALLAL
VNMVSRLLLNRFTMT
VVYDAKFEK
GPLKLFMAL
FQTTTGEIGAIALDF
RYLEFEALGFLNEDH
IDLDPVVYDAKFEKQ
LMWKQITPEL
HTALTGATEIQTSGT
RENLLLGVGLAMATTLQLPE
FGMFTTNIWMKFREG
MFTTNIWMKF
MTSRMLLNRFTMAHR
RWGTIKKSKAINVLR
WGTIKKSKAINVLRG
ILRNPGFALLAGFMA
KQIANELNY
KMVHQIFGSAYTALF
MQRKHGGML
RMLLNRFTMAHRKPT
SPIINREGKV
ARYLEFEALGFLNED
YYMATLKNVT
YYMATLKNV
QYENLKYSVIVTVHT
VLDDGIYRI
IPITAAAWYL
IPITAAAWY
REGKVVGLYGNGVVT
TLILAPTRVVASEMA
RTLILAPTRVVASEM
IKKSKAINVLRGFRK
ALVAFLRFLTIPPTA
LVAFLRFLTIPPTAG
RPMFAVGLLF
ASGINMCTLIAMDLG
EEREIPERSW
NPEIEDDIF
GMNSRSTSLSVSLVL
GEKWKRQLNQLSKSE
PFNMLKRERNRVSTV
EGKVVGLYGNGVVTK
YTPEGIIPTL
KQIANELNYILWENN
TGNIVSAVNMTSRML
AVNMTSRMLLNRFTM
GTGNIVSAVNMTSRM
LMLLALIAVLTGGVT
FALLAGFMAYMIGQT
ISKVRSNAAIGAVFQ
KVRSNAAIGAVFQEE
ETTILDVDLRPASAW
IENTSANLSL
CYSQVNPTTL
SILLSSLLK
TLLLLGLMILLTGGA
AVGLLFRRLTSREVL
VGLLFRRLTSREVLL
GLLFRRLTSREVLLL
LKGLPIRYQTTATKS
GGVLNSLGKMVHQIF
SLGVLGMALFLEEML
TPFNMLKRERNRVST
LGKMVHQIFGSAYTA
DFNEMILLTMKNKAW
AAAIFMTATPPGSVE
EMILLTMKNKAWMVH
C
NS1
NS2b
NS4b
prM
E
NS2a
NS3
NS5
Fraction of sequences
0
0.5
1

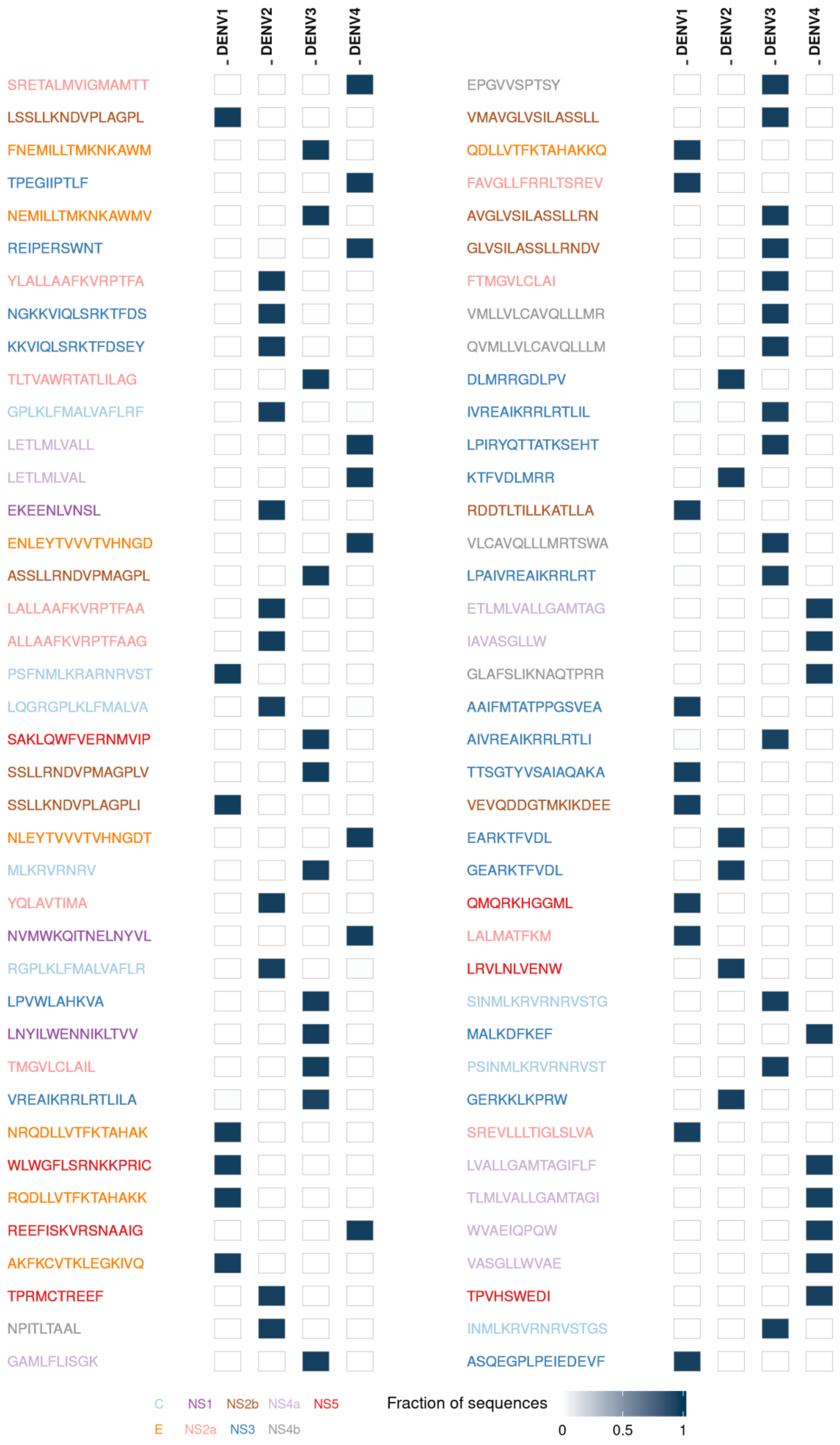
_DENV1
_DENV2
_DENV3
_DENV4
SRETALMVIGMAMTT
LSSLLKNDVPLAGPL
FNEMILLTMKNKAWM
TPEGIIPTLF
NEMILLTMKNKAWMV
REIPERSWNT
YLALLAAFKVRPTFA
NGKKVIQLSRKTFDS
KKVIQLSRKTFDSEY
TLTVAWRTATLILAG
GPLKLFMALVAFLRF
LETLMLVALL
LETLMLVAL
EKEENLVNSL
ENLEYTVVVTVHNGD
ASSLLRNDVPMAGPL
LALLAAFKVRPTFAA
ALLAAFKVRPTFAAG
PSFNMLKRARNRVST
LQGRGPLKLFMALVA
SAKLQWFVERNMVIP
SSLLRNDVPMAGPLV
SSLLKNDVPLAGPLI
NLEYTVVVTVHNGDT
MLKRVRNRV
YQLAVTIMA
NVMWKQITNELNYVL
RGPLKLFMALVAFLR
LPVWLAHKVA
LNYILWENNIKLTVV
TMGVLCLAIL
VREAIKRRLRTLILA
NRQDLLVTFKTAHAK
WLWGFLSRNKKPRIC
RQDLLVTFKTAHAKK
REEFISKVRSNAAIG
AKFKCVTKLEGKIVQ
TPRMCTREEF
NPITLTAAL
GAMLFLISGK
_DENV1
_DENV2
_DENV3
_DENV4
EPGVVSPTSY
VMAVGLVSILASSLL
QDLLVTFKTAHAKKQ
FAVGLLFRRLTSREV
AVGLVSILASSLLRN
GLVSILASSLLRNDV
FTMGVLCLAI
VMLLVLCAVQLLLMR
QVMLLVLCAVQLLLM
DLMRRGDLPV
IVREAIKRRLRTLIL
LPIRYQTTATKSEHT
KTFVDLMRR
RDDTLTILLKATLLA
VLCAVQLLLMRTSWA
LPAIVREAIKRRLRT
ETLMLVALLGAMTAG
IAVASGLLW
GLAFSLIKNAQTPRR
AAIFMTATPPGSVEA
AIVREAIKRRLRTLI
TTSGTYVSAIAQAKA
VEVQDDGTMKIKDEE
EARKTFVDL
GEARKTFVDL
QMQRKHGGML
LALMATFKM
LRVLNLVENW
SINMLKRVRNRVSTG
MALKDFKEF
PSINMLKRVRNRVST
GERKKLKPRW
SREVLLLTIGLSLVA
LVALLGAMTAGIFLF
TLMLVALLGAMTAGI
WVAEIQPQW
VASGLLWVAE
TPVHSWEDI
INMLKRVRNRVSTGS
ASQEGPLPEIEDEVF
C
NS1
NS2b
NS4a
NS5
E
NS2a
NS3
NS4b
Fraction of sequences
0
0.5
1

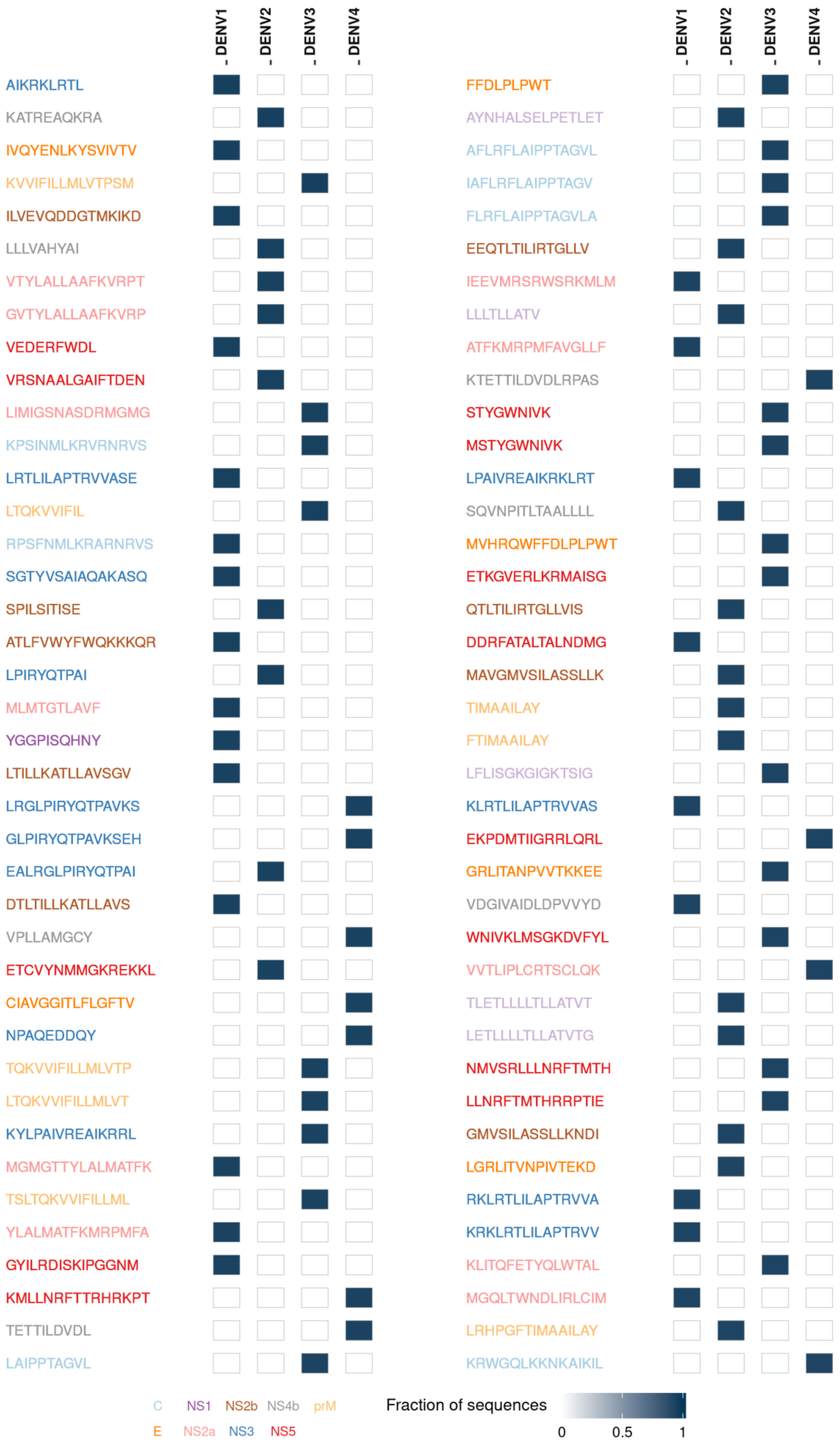
DENV1
DENV2
DENV3
DENV4
AIKRKLRTL
KATREAQKRA
IVQYENLKYSVIVTV
KVVIFILLMLVTPSM
ILVEVQDDGTMKIKD
LLLVAHYAI
VTYLALLAAFKVRPT
GVTYLALLAAFKVRP
VEDERFWDL
VRSNAALGAIFTDEN
LIMIGSNASDRMGMG
KPSINMLKRVRNRVS
LRTLILAPTRVVASE
LTQKVVIFIL
RPSFNMLKRARNRVS
SGTYVSAIAQAKASQ
SPILSITISE
ATLFVWYFWQKKKQR
LPIRYQTPAI
MLMTGTLAVF
YGGPISQHNY
LTILLKATLLAVSGV
LRGLPIRYQTPAVKS
GLPIRYQTPAVKSEH
EALRGLPIRYQTPAI
DTLTILLKATLLAVS
VPLLAMGCY
ETCVYNMMGKREKKL
CIAVGGITLFLGFTV
NPAQEDDQY
TQKVVIFILLMLVTP
LTQKVVIFILLMLVT
KYLPAIVREAIKRRL
MGMGTTYLALMATFK
TSLTQKVVIFILLML
YLALMATFKMRPMFA
GYILRDISKIPGGNM
KMLLNRFTTRHRKPT
TETTILDVDL
LAIPPTAGVL
FFDLPLPWT
AYNHALSELPETLET
AFLRFLAIPPTAGVL
IAFLRFLAIPPTAGV
FLRFLAIPPTAGVLA
EEQTLTILIRTGLLV
IEEVMRSRWSRKMLM
LLLTLLATV
ATFKMRPMFAVGLLF
KTETTILDVDLRPAS
STYGWNIVK
MSTYGWNIVK
LPAIVREAIKRKLRT
SQVNPITLTAALLLL
MVHRQWFFDLPLPWT
ETKGVERLKRMAISG
QTLTILIRTGLLVIS
DDRFATALTALNDMG
MAVGMVSILASSLLK
TIMAAILAY
FTIMAAILAY
LFLISGKGIGKTSIG
KLRTLILAPTRVVAS
EKPDMTIIGRRLQRL
GRLITANPVVTKKEE
VDGIVAIDLDPVVYD
WNIVKLMSGKDVFYL
VVTLIPLCRTSCLQK
TLETLLLLTLLATVT
LETLLLLTLLATVTG
NMVSRLLLNRFTMTH
LLNRFTMTHRRPTIE
GMVSILASSLLKNDI
LGRLITVNPIVTEKD
RKLRTLILAPTRVVA
KRKLRTLILAPTRVV
KLITQFETYQLWTAL
MGQLTWNDLIRLCIM
LRHPGFTIMAAILAY
KRWGQLKKNKAIKIL
C
NS1
NS2b
NS4b
prM
E
NS2a
NS3
NS5
Fraction of sequences
0
0.5
1

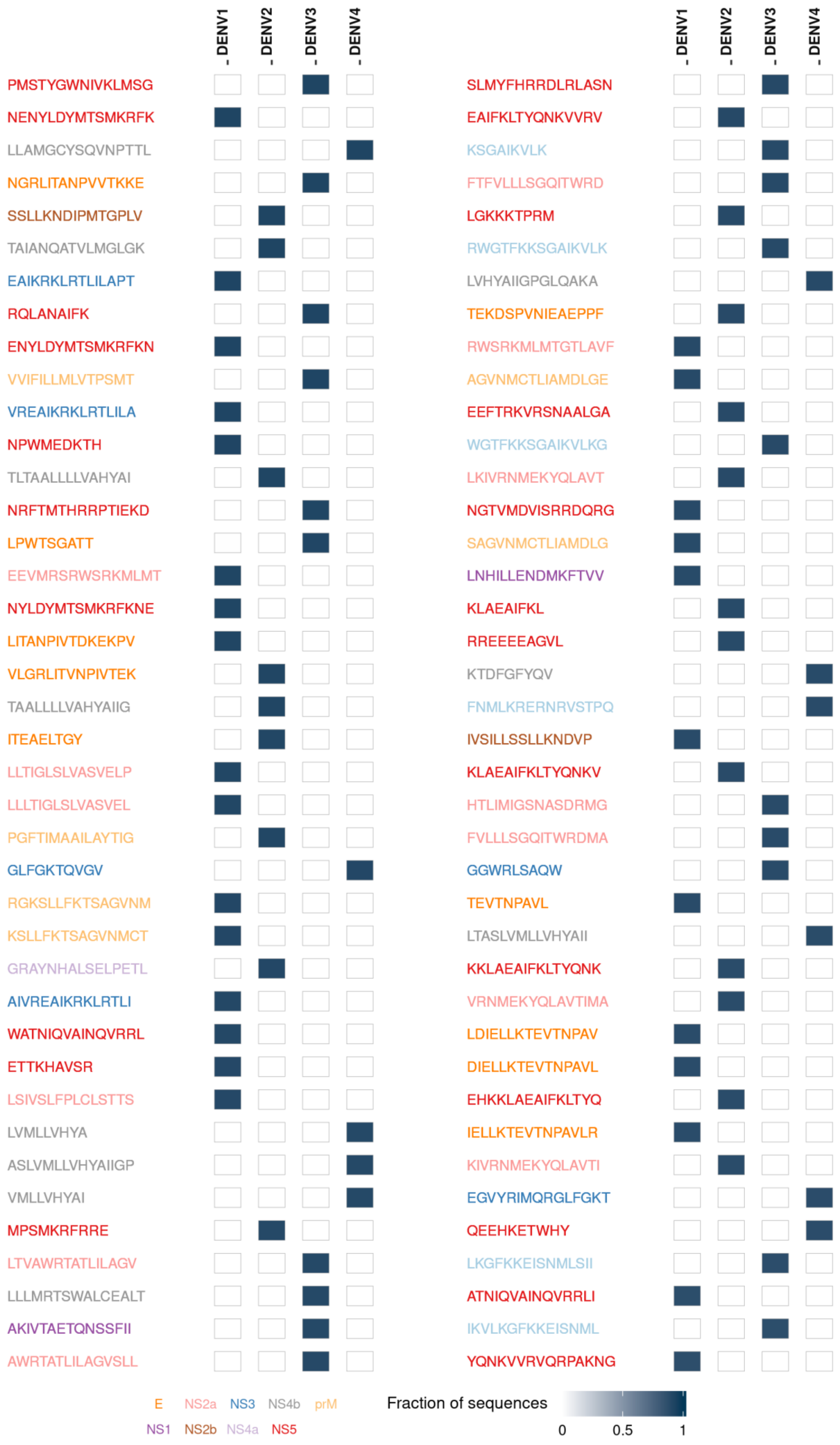

DENV1
DENV2
DENV3
DENV4
PMSTYGWNIVKLMSG
NENYLDYMTSMKRFK
LLAMGCYSQVNPTTL
NGRLITANPVVTKKE
SSLLKNDIPMTGPLV
TAIANQATVLMGLGK
EAIKRKLRTLILAPT
RQLANAIFK
ENYLDYMTSMKRFKN
VVIFILLMLVTPSMT
VREAIKRKLRTLILA
NPWMEDKTH
TLTAALLLLVAHYAI
NRFTMTHRRPTIEKD
LPWTSGATT
EEVMRSRWSRKMLMT
NYLDYMTSMKRFKNE
LITANPIVTDKEKPV
VLGRLITVNPIVTEK
TAALLLLVAHYAIIG
ITEAELTGY
LLTIGLSLVASVELP
LLLTIGLSLVASVEL
PGFTIMAAILAYTIG
GLFGKTQVGV
RGKSLLFKTSAGVNM
KSLLFKTSAGVNMCT
GRAYNHALSELPETL
AIVREAIKRKLRTLI
WATNIQVAINQVRRL
ETTKHAVSR
LSIVSLFPLCLSTTS
LVMLLVHYA
ASLVMLLVHYAIIGP
VMLLVHYAI
MPSMKRFRRE
LTVAWRTATLILAGV
LLLMRTSWALCEALT
AKIVTAETQNSSFII
AWRTATLILAGVSLL
SLMYFHRRDLRLASN
EAIFKLTYQNKVVRV
KSGAIKVLK
FTFVLLLSGQITWRD
LGKKKTPRM
RWGTFKKSGAIKVLK
LVHYAIIGPGLQAKA
TEKDSPVNIEAEPPF
RWSRKMLMTGTLAVF
AGVNMCTLIAMDLGE
EEFTRKVRSNAALGA
WGTFKKSGAIKVLKG
LKIVRNMEKYQLAVT
NGTVMDVISRRDQRG
SAGVNMCTLIAMDLG
LNHILLENDMKFTVV
KLAEAIFKL
RREEEEAGVL
KTDFGFYQV
FNMLKRERNRVSTPQ
IVSILLSSLLKNDVP
KLAEAIFKLTYQNKV
HTLIMIGSNASDRMG
FVLLLSGQITWRDMA
GGWRLSAQW
TEVTNPAVL
LTASLVMLLVHYAII
KKLAEAIFKLTYQNK
VRNMEKYQLAVTIMA
LDIELLKTEVTNPAV
DIELLKTEVTNPAVL
EHKKLAEAIFKLTYQ
IELLKTEVTNPAVLR
KIVRNMEKYQLAVTI
EGVYRIMQRGLFGKT
QEEHKETWHY
LKGFKKEISNMLSII
ATNIQVAINQVRRLI
IKVLKGFKKEISNML
YQNKVVRVQRPAKNG
E
NS2a
NS3
NS4b
prM
NS1
NS2b
NS4a
NS5
Fraction of sequences
0
0.5
1

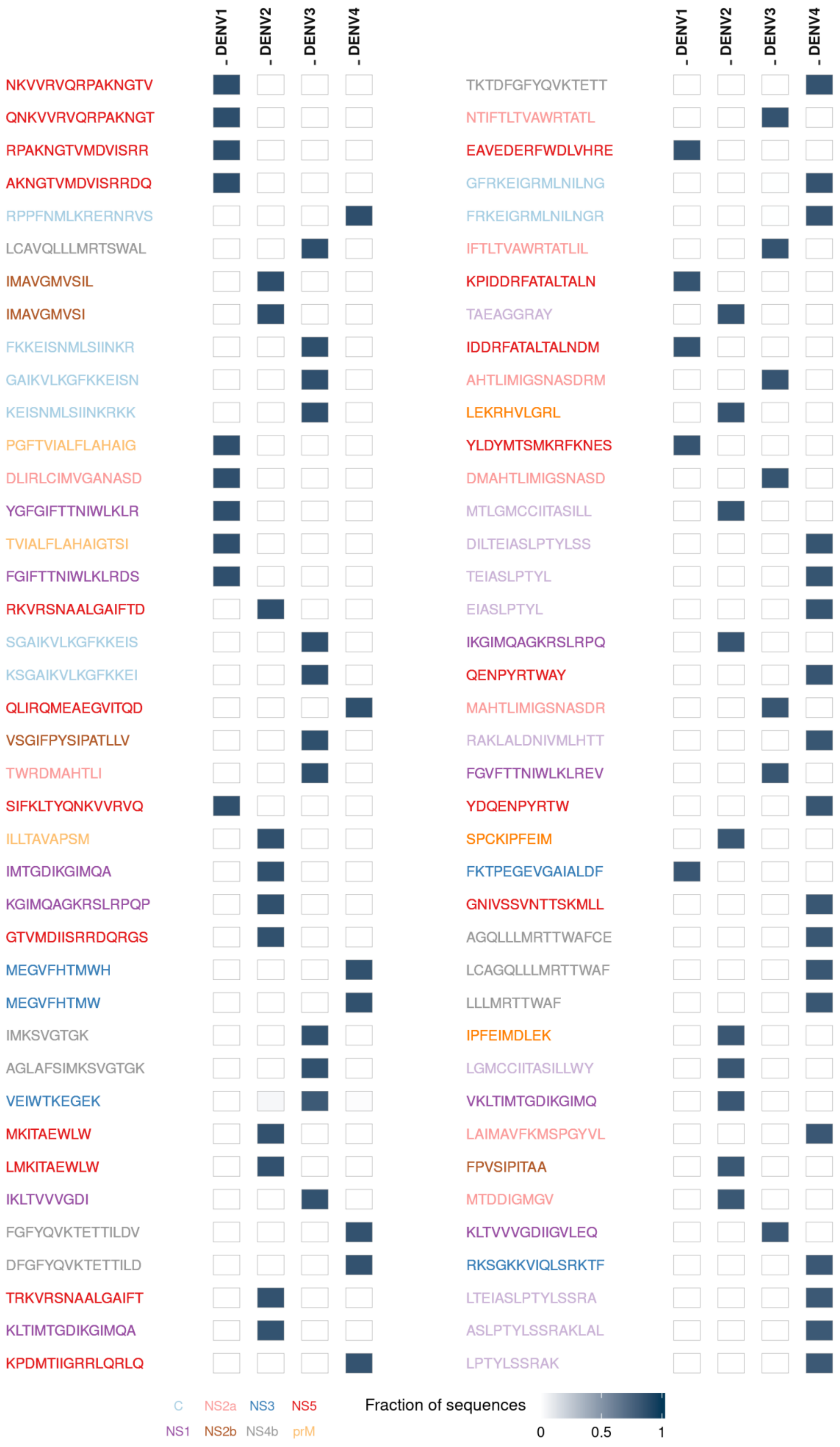

DENV1
DENV2
DENV3
DENV4
NKVVRVQRPAKNGTV
QNKVVRVQRPAKNGT
RPAKNGTVMDVISRR
AKNGTVMDVISRRDQ
RPPFNMLKRERNRVS
LCAVQLLLMRTSWAL
IMAVGMVSIL
IMAVGMVSI
FKKEISNMLSIINKR
GAIKVLKGFKKEISN
KEISNMLSIINKRKK
PGFTVIALFLAHAIG
DLIRLCIMVGANASD
YGFGIFTTNIWLKLR
TVIALFLAHAIGTSI
FGIFTTNIWLKLRDS
RKVRSNAALGAIFTD
SGAIKVLKGFKKEIS
KSGAIKVLKGFKKEI
QLIRQMEAEGVITQD
VSGIFPYSIPATLLV
TWRDMAHTLI
SIFKLTYQNKVVRVQ
ILLTAVAPSM
IMTGDIKGIMQA
KGIMQAGKRSLRPQP
GTVMDIISRRDQRGS
MEGVFHTMWH
MEGVFHTMW
IMKSVGTGK
AGLAFSIMKSVGTGK
VEIWTKEGEK
MKITAEWLW
LMKITAEWLW
IKLTVVVGDI
FGFYQVKTETTILDV
DFGFYQVKTETTILD
TRKVRSNAALGAIFT
KLTIMTGDIKGIMQA
KPDMTIIGRRLQRLQ
TKTDFGFYQVKTETT
NTIFTLTVAWRTATL
EAVEDERFWDLVHRE
GFRKEIGRMLNILNG
FRKEIGRMLNILNGR
IFTLTVAWRTATLIL
KPIDDRFATALTALN
TAEAGGRAY
IDDRFATALTALNDM
AHTLIMIGSNASDRM
LEKRHVLGRL
YLDYMTSMKRFKNES
DMAHTLIMIGSNASD
MTLGMCCIITASILL
DILTEIASLPTYLSS
TEIASLPTYL
EIASLPTYL
IKGIMQAGKRSLRPQ
QENPYRTWAY
MAHTLIMIGSNASDR
RAKLALDNIVMLHTT
FGVFTTNIWLKLREV
YDQENPYRTW
SPCKIPFEIM
FKTPEGEVGAIALDF
GNIVSSVNTTSKMLL
AGQLLLMRTTWAFCE
LCAGQLLLMRTTWAF
LLLMRTTWAF
IPFEIMDLEK
LGMCCIITASILLWY
VKLTIMTGDIKGIMQ
LAIMAVFKMSPGYVL
FPVSIPITAA
MTDDIGMGV
KLTVVVGDIIGVLEQ
RKSGKKVIQLSRKTF
LTEIASLPTYLSSRA
ASLPTYLSSRAKLAL
LPTYLSSRAK
C
NS2a
NS3
NS5
NS1
NS2b
NS4b
prM
Fraction of sequences
0
0.5
1

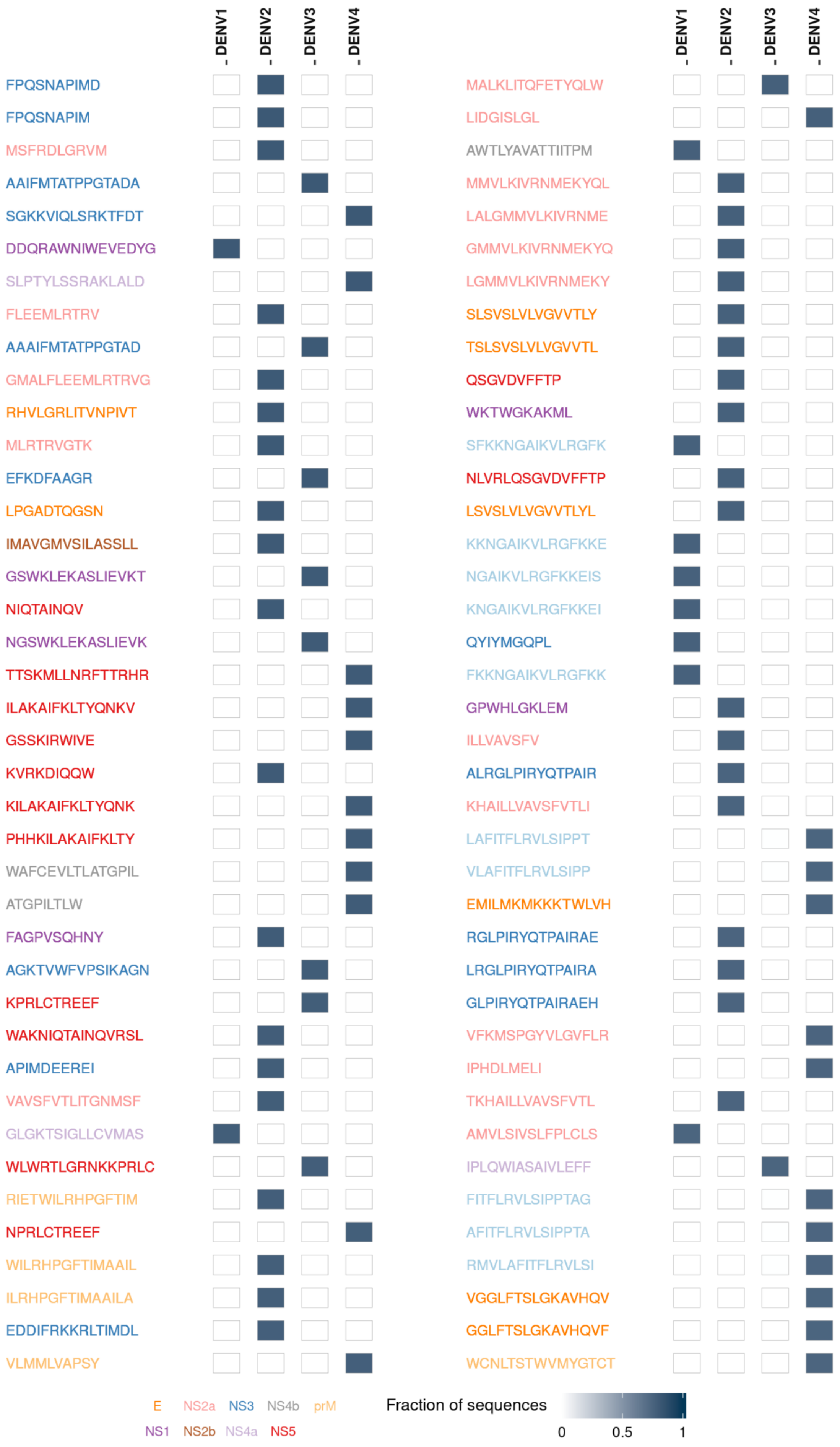
DENV1
DENV2
DENV3
DENV4
FPQSNAPIMD
FPQSNAPIM
MSFRDLGRVM
AAIFMTATPPGTADA
SGKKVIQLSRKTFDT
DDQRAWNIWEVEDYG
SLPTYLSSRAKLALD
FLEEMLRTRV
AAAIFMTATPPGTAD
GMALFLEEMLRTRVG
RHVLGRLITVNPIVT
MLRTRVGTK
EFKDFAAGR
LPGADTQGSN
IMAVGMVSILASSLL
GSWKLEKASLIEVKT
NIQTAINQV
NGSWKLEKASLIEVK
TTSKMLLNRFTTRHR
ILAKAIFKLTYQNKV
GSSKIRWIVE
KVRKDIQQW
KILAKAIFKLTYQNK
PHHKILAKAIFKLTY
WAFCEVLTLATGPIL
ATGPILTLW
FAGPVSQHNY
AGKTVWFVPSIKAGN
KPRLCTREEF
WAKNIQTAINQVRSL
APIMDEEREI
VAVSFVTLITGNMSF
GLGKTSIGLLCVMAS
WLWRTLGRNKKPRLC
RIETWILRHPGFTIM
NPRLCTREEF
WILRHPGFTIMAAIL
ILRHPGFTIMAAILA
EDDIFRKKRLTIMDL
VLMMLVAPSY
MALKLITQFETYQLW
LIDGISLGL
AWTLYAVATTIITPM
MMVLKIVRNMEKYQL
LALGMMVLKIVRNME
GMMVLKIVRNMEKYQ
LGMMVLKIVRNMEKY
SLSVSLVLVGVVTLY
TSLSVSLVLVGVVTL
QSGVDVFFTP
WKTWGKAKML
SFKKNGAIKVLRGFK
NLVRLQSGVDVFFTP
LSVSLVLVGVVTLYL
KKNGAIKVLRGFKKE
NGAIKVLRGFKKEIS
KNGAIKVLRGFKKEI
QYIYMGQPL
FKKNGAIKVLRGFKK
GPWHLGKLEM
ILLVAVSFV
ALRGLPIRYQTPAIR
KHAILLVAVSFVTLI
LAFITFLRVLSIPPT
VLAFITFLRVLSIPP
EMILMKMKKKTWLVH
RGLPIRYQTPAIRAE
LRGLPIRYQTPAIRA
GLPIRYQTPAIRAEH
VFKMSPGYVLGVFLR
IPHDLMELI
TKHAILLVAVSFVTL
AMVLSIVSLFPLCLS
IPLQWIASAIVLEFF
FITFLRVLSIPPTAG
AFITFLRVLSIPPTA
RMVLAFITFLRVLSI
VGGLFTSLGKAVHQV
GGLFTSLGKAVHQVF
WCNLTSTWVMYGTCT
E
NS2a
NS3
NS4b
prM
NS1
NS2b
NS4a
NS5
Fraction of sequences
0
0.5
1

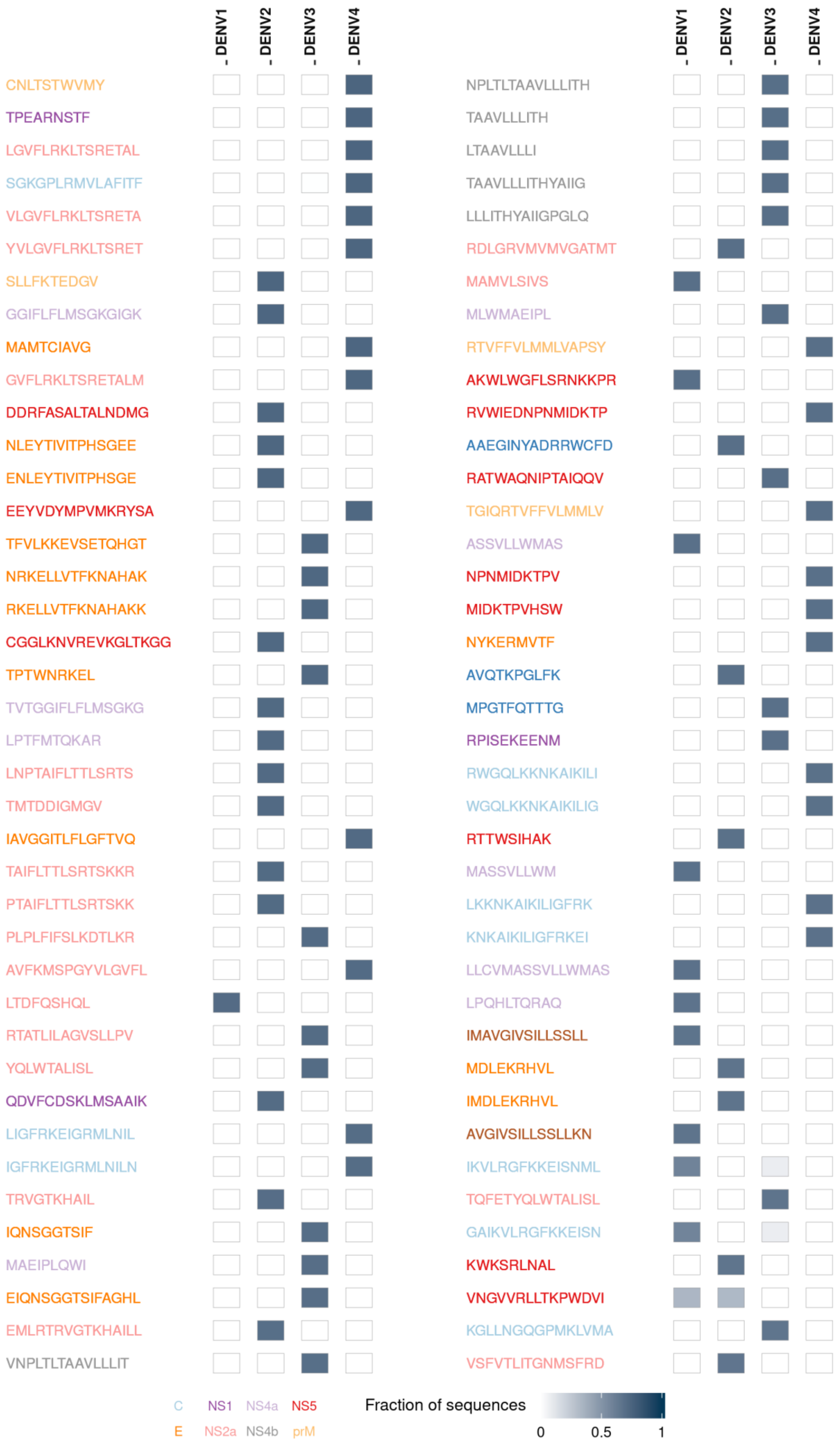

_DENV1
_DENV2
_DENV3
_DENV4
CNLTSTWVMY
TPEARNSTF
LGVFLRKLTSRETAL
SGKGPLRMVLAFITF
VLGVFLRKLTSRETA
YVLGVFLRKLTSRET
SLLFKTEDGV
GGIFLFLMSGKGIGK
MAMTCIAVG
GVFLRKLTSRETALM
DDRFASALTALNDMG
NLEYTIVITPHSGEE
ENLEYTIVITPHSGE
EEYVDYMPVMKRYSA
TFVLKKEVSETQHGT
NRKELLVTFKNAHAK
RKELLVTFKNAHAKK
CGGLKNVREVKGLTKGG
TPTWNRKEL
TVTGGIFLFLMSGKG
LPTFMTQKAR
LNPTAIFLTTLSRTS
TMTDDIGMGV
IAVGGITLFLGFTVQ
TAIFLTTLSRTSKKR
PTAIFLTTLSRTSKK
PLPLFIFSLKDTLKR
AVFKMSPGYVLGVFL
LTDFQSHQL
RTATLILAGVSLLPV
YQLWTALISL
QDVFCDSKLMSAAIK
LIGFRKEIGRMLNIL
IGFRKEIGRMLNILN
TRVGTKHAIL
IQNSGGTSIF
MAEIPLQWI
EIQNSGGTSIFAGHL
EMLRTRVGTKHAILL
VNPLTLTAAVLLLIT
NPLTLTAAVLLLITH
TAAVLLLITH
LTAAVLLLI
TAAVLLLITHYAIIG
LLLITHYAIIGPGLQ
RDLGRVMVMVGATMT
MAMVLSIVS
MLWMAEIPL
RTVFFVLMMLVAPSY
AKWLWGFLSRNKKPR
RVWIEDNPNMIDKTP
AAEGINYADRRWCFD
RATWAQNIPTAIQQV
TGIQRTVFFVLMMLV
ASSVLLWMAS
NPNMIDKTPV
MIDKTPVHSW
NYKERMVTF
AVQTKPGLFK
MPGTFQTTTG
RPISEKEENM
RWGQLKKNKAIKILI
WGQLKKNKAIKILIG
RTTWSIHAK
MASSVLLWM
LKKNKAIKILIGFRK
KNKAIKILIGFRKEI
LLCVMASSVLLWMAS
LPQHLTQRAQ
IMAVGIVSILLSSLL
MDLEKRHVL
IMDLEKRHVL
AVGIVSILLSSLLKN
IKVLRGFKKEISNML
TQFETYQLWTALISL
GAIKVLRGFKKEISN
KWKSRLNAL
VNGVVRLLTKPWDVI
KGLLNGQGPMKLVMA
VSFVTLITGNMSFRD
C
NS1
NS4a
NS5
E
NS2a
NS4b
prM
Fraction of sequences
0
0.5
1

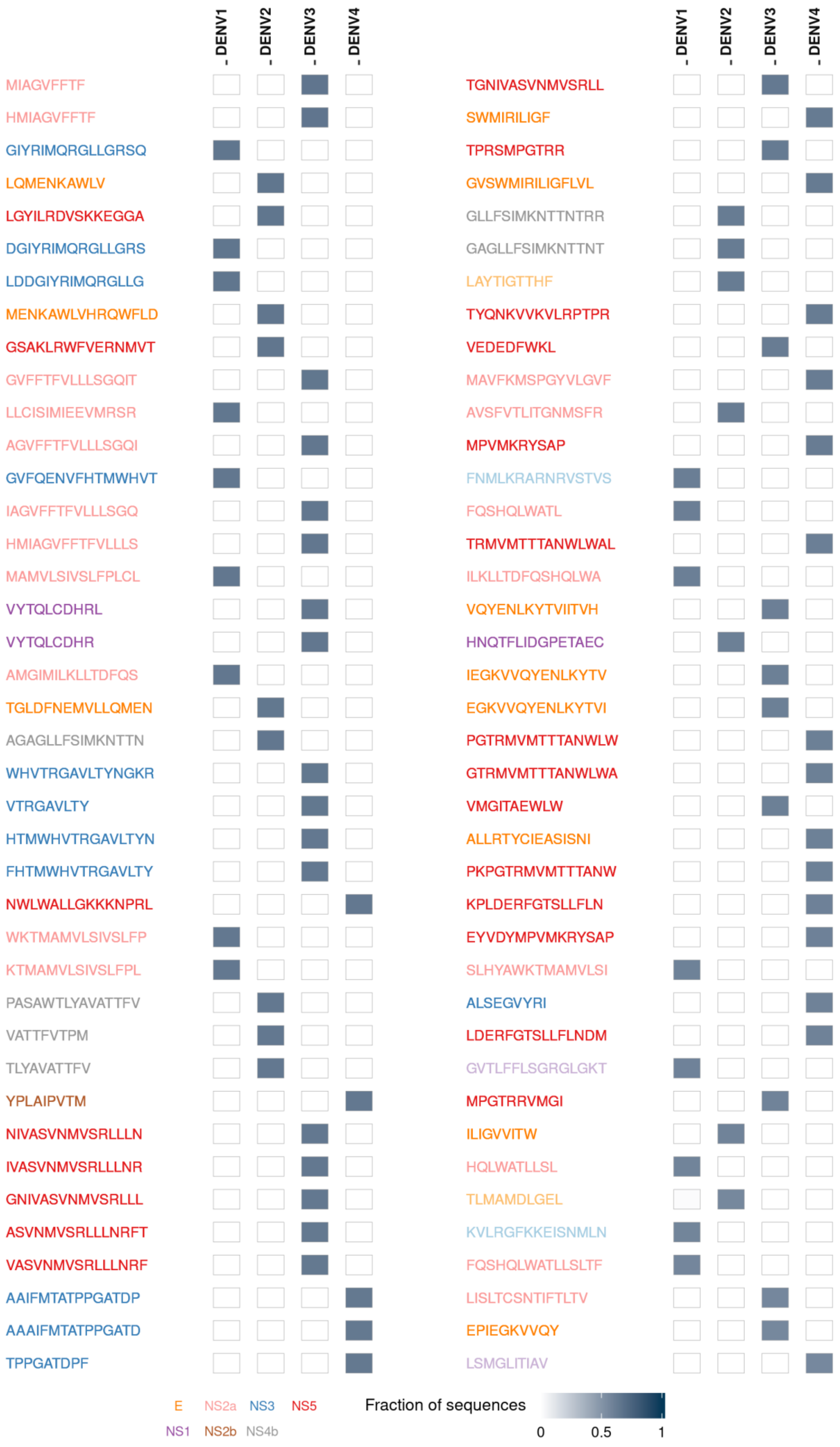
DENV1
DENV2
DENV3
DENV4
MIAGVFFTF
HMIAGVFFTF
GIYRIMQRGLLGRSQ
LQMENKAWLV
LGYILRDVSKKEGGA
DGIYRIMQRGLLGRS
LDDGIYRIMQRGLLG
MENKAWLVHRQWFLD
GSAKLRWFVERNMVT
GVFFTFVLLLSGQIT
LLCISIMIEEVMRSR
AGVFFTFVLLLSGQI
GVFQENVFHTMWHVT
IAGVFFTFVLLLSGQ
HMIAGVFFTFVLLLS
MAMVLSIVSLFPLCL
VYTQLCDHRL
VYTQLCDHR
AMGIMILKLLTDFQS
TGLDFNEMVLLQMEN
AGAGLLFSIMKNTTN
WHVTRGAVLTYNGKR
VTRGAVLTY
HTMWHVTRGAVLTYN
FHTMWHVTRGAVLTY
NWLWALLGKKKNPRL
WKTMAMVLSIVSLFP
KTMAMVLSIVSLFPL
PASAWTLYAVATTFV
VATTFVTPM
TLYAVATTFV
YPLAIPVTM
NIVASVNMVSRLLLN
IVASVNMVSRLLLNR
GNIVASVNMVSRLLL
ASVNMVSRLLLNRFT
VASVNMVSRLLLNRF
AAIFMTATPPGATDP
AAAIFMTATPPGATD
TPPGATDPF
TGNIVASVNMVSRLL
SWMIRILIGF
TPRSMPGTRR
GVSWMIRILIGFLVL
GLLFSIMKNTTNTRR
GAGLLFSIMKNTTNT
LAYTIGTTHF
TYQNKVVKVLRPTPR
VEDEDFWKL
MAVFKMSPGYVLGVF
AVSFVTLITGNMSFR
MPVMKRYSAP
FNMLKRARNRVSTVS
FQSHQLWATL
TRMVMTTTANWLWAL
ILKLLTDFQSHQLWA
VQYENLKYTVIITVH
HNQTFLIDGPETAEC
IEGKVVQYENLKYTV
EGKVVQYENLKYTVI
PGTRMVMTTTANWLW
GTRMVMTTTANWLWA
VMGITAEWLW
ALLRTYCIEASISNI
PKPGTRMVMTTTANW
KPLDERFGTSLLFLN
EYVDYMPVMKRYSAP
SLHYAWKTMAMVLSI
ALSEGVYRI
LDERFGTSLLFLNDM
GVTLFFLSGRGLGKT
MPGTRRVMGI
ILIGVVITW
HQLWATLLSL
TLMAMDLGEL
KVLRGFKKEISNMLN
FQSHQLWATLLSLTF
LISLTCSNTIFTLTV
EPIEGKVVQY
LSMGLITIAV
E
NS2a
NS3
NS5
NS1
NS2b
NS4b
Fraction of sequences
0
0.5
1

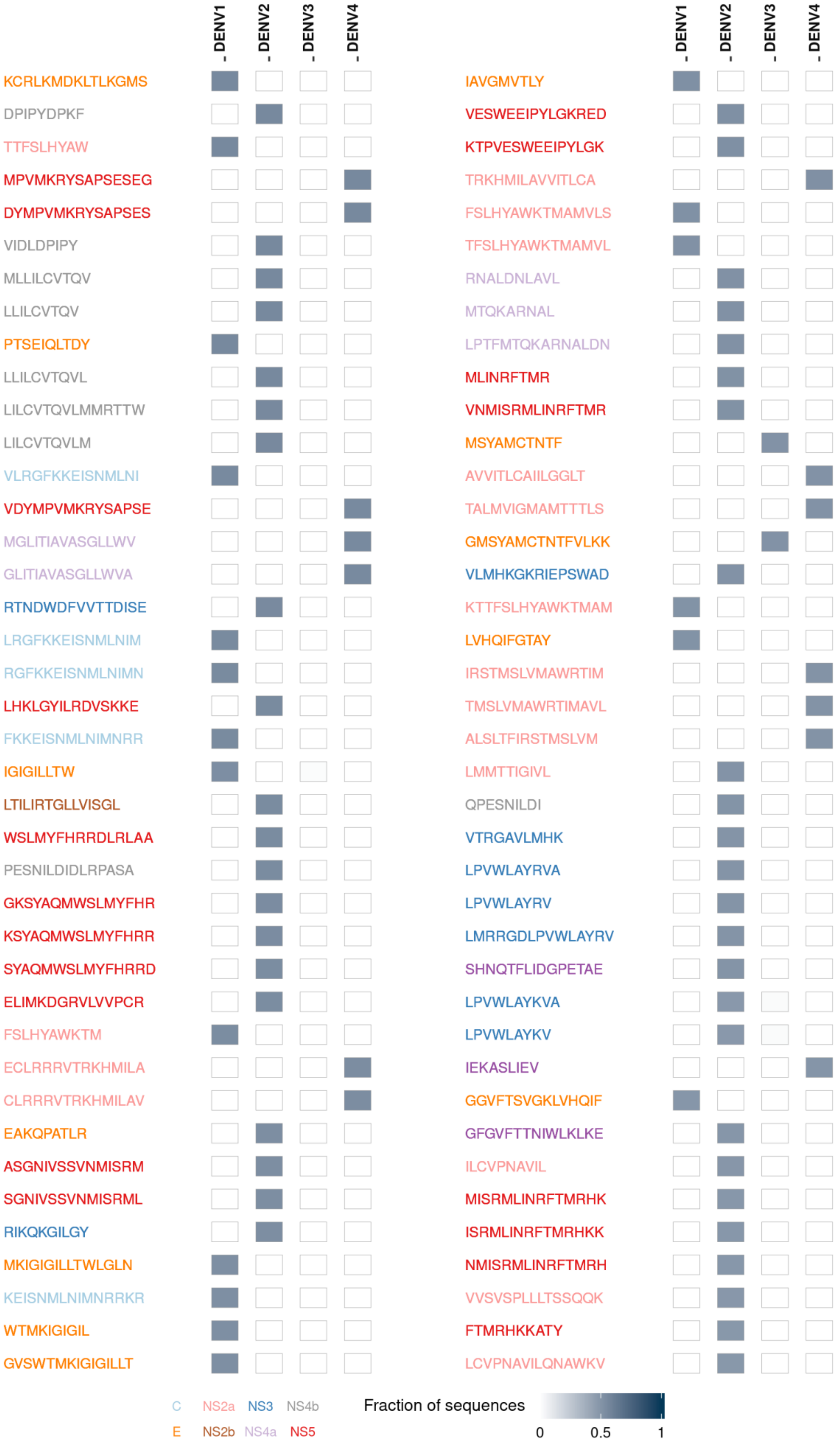
DENV1
DENV2
DENV3
DENV4
KCRLKMDKLTLKGMS
DPIPYDPKF
TTFSLHYAW
MPVMKRYSAPSESEG
DYMPVMKRYSAPSES
VIDLDPIPY
MLLILCVTQV
LLILCVTQV
PTSEIQLTDY
LLILCVTQVL
LILCVTQVLMMRTTW
LILCVTQVLM
VLRGFKKEISNMLNI
VDYMPVMKRYSAPSE
MGLITIAVASGLLWV
GLITIAVASGLLWVA
RTNDWDFVVTTDISE
LRGFKKEISNMLNIM
RGFKKEISNMLNIMN
LHKLGYILRDVSKKE
FKKEISNMLNIMNRR
IGIGILLTW
LTILIRTGLLVISGL
WSLMYFHRRDLRLAA
PESNILDIDLRPASA
GKSYAQMWSLMYFHR
KSYAQMWSLMYFHRR
SYAQMWSLMYFHRRD
ELIMKDGRVLVVPCR
FSLHYAWKTM
ECLRRRVTRKHMILA
CLRRRVTRKHMILAV
EAKQPATLR
ASGNIVSSVNMISRM
SGNIVSSVNMISRML
RIKQKGILGY
MKIGIGILLTWLGLN
KEISNMLNIMNRRKR
WTMKIGIGIL
GVSWTMKIGIGILLT
IAVGMVTLY
VESWEEIPYLGKRED
KTPVESWEEIPYLGK
TRKHMILAVVITLCA
FSLHYAWKTMAMVLS
TFSLHYAWKTMAMVL
RNALDNLAVL
MTQKARNAL
LPTFMTQKARNALDN
MLINRFTMR
VNMISRMLINRFTMR
MSYAMCTNTF
AVVITLCAIILGGLT
TALMVIGMAMTTTLS
GMSYAMCTNTFVLKK
VLMHKGKRIEPSWAD
KTTFSLHYAWKTMAM
LVHQIFGTAY
IRSTMSLVMAWRTIM
TMSLVMAWRTIMAVL
ALSLTFIRSTMSLVM
LMMTTIGIVL
QPESNILDI
VTRGAVLMHK
LPVWLAYRVA
LPVWLAYRV
LMRRGDLPVWLAYRV
SHNQTFLIDGPETAE
LPVWLAYKVA
LPVWLAYKV
IEKASLIEV
GGVFTSVGKLVHQIF
GFGVFTTNIWLKLKE
ILCVPNAVIL
MISRMLINRFTMRHK
ISRMLINRFTMRHKK
NMISRMLINRFTMRH
VVSVSPLLLTSSQQK
FTMRHKKATY
LCVPNAVILQNAWKV
C
NS2a
NS3
NS4b
E
NS2b
NS4a
NS5
Fraction of sequences
0
0.5
1

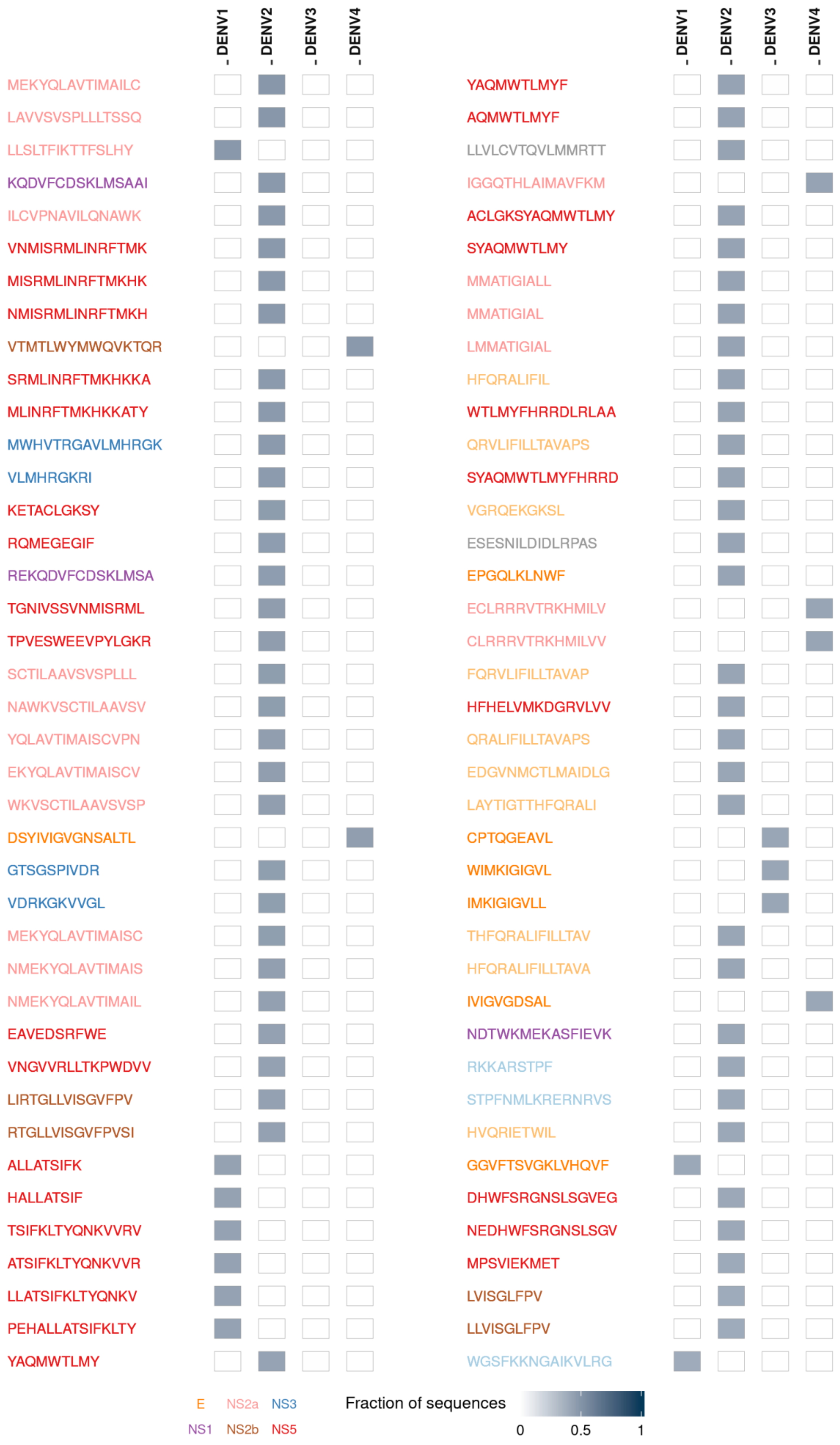
DENV1
DENV2
DENV3
DENV4
MEKYQLAVTIMAILC
LAVVSVSPLLLTSSQ
LLSLTFIKTTFSLHY
KQDVFCDSKLMSAAI
ILCVPNAVILQNAWK
VNMISRMLINRFTMK
MISRMLINRFTMKHK
NMISRMLINRFTMKH
VTMTLWYMWQVKTQR
SRMLINRFTMKHKKA
MLINRFTMKHKKATY
MWHVTRGAVLMHRGK
VLMHRGKRI
KETACLGKSY
RQMEGEGIF
REKQDVFCDSKLMSA
TGNIVSSVNMISRML
TPVESWEEVPYLGKR
SCTILAAVSVSPLLL
NAWKVSCTILAAVSV
YQLAVTIMAISCVPN
EKYQLAVTIMAISCV
WKVSCTILAAVSVSP
DSYIVIGVGNSALTL
GTSGSPIVDR
VDRKGKVVGL
MEKYQLAVTIMAISC
NMEKYQLAVTIMAIS
NMEKYQLAVTIMAIL
EAVEDSRFWE
VNGVVRLLTKPWDVV
LIRTGLLVISGVFPV
RTGLLVISGVFPVSI
ALLATSIFK
HALLATSIF
TSIFKLTYQNKVVRV
ATSIFKLTYQNKVVR
LLATSIFKLTYQNKV
PEHALLATSIFKLTY
YAQMWTLMY
YAQMWTLMYF
AQMWTLMYF
LLVLCVTQVLMMRTT
IGGQTHLAIMAVFKM
ACLGKSYAQMWTLMY
SYAQMWTLMY
MMATIGIALL
MMATIGIAL
LMMATIGIAL
HFQRALIFIL
WTLMYFHRRDLRLAA
QRVLIFILLTAVAPS
SYAQMWTLMYFHRRD
VGRQEKGKSL
ESESNILDIDLRPAS
EPGQLKLNWF
ECLRRRVTRKHMILV
CLRRRVTRKHMILVV
FQRVLIFILLTAVAP
HFHELVMKDGRVLVV
QRALIFILLTAVAPS
EDGVNMCTLMAIDLG
LAYTIGTTHFQRALI
CPTQGEAVL
WIMKIGIGVL
IMKIGIGVLL
THFQRALIFILLTAV
HFQRALIFILLTAVA
IVIGVGDSAL
NDTWKMEKASFIEVK
RKKARSTPF
STPFNMLKRERNRVS
HVQRIETWIL
GGVFTSVGKLVHQVF
DHWFSRGNSLSGVEG
NEDHWFSRGNSLSGV
MPSVIEKMET
LVISGLFPV
LLVISGLFPV
WGSFKKNGAIKVLRG
E
NS2a
NS3
NS1
NS2b
NS5
Fraction of sequences
0
0.5
1

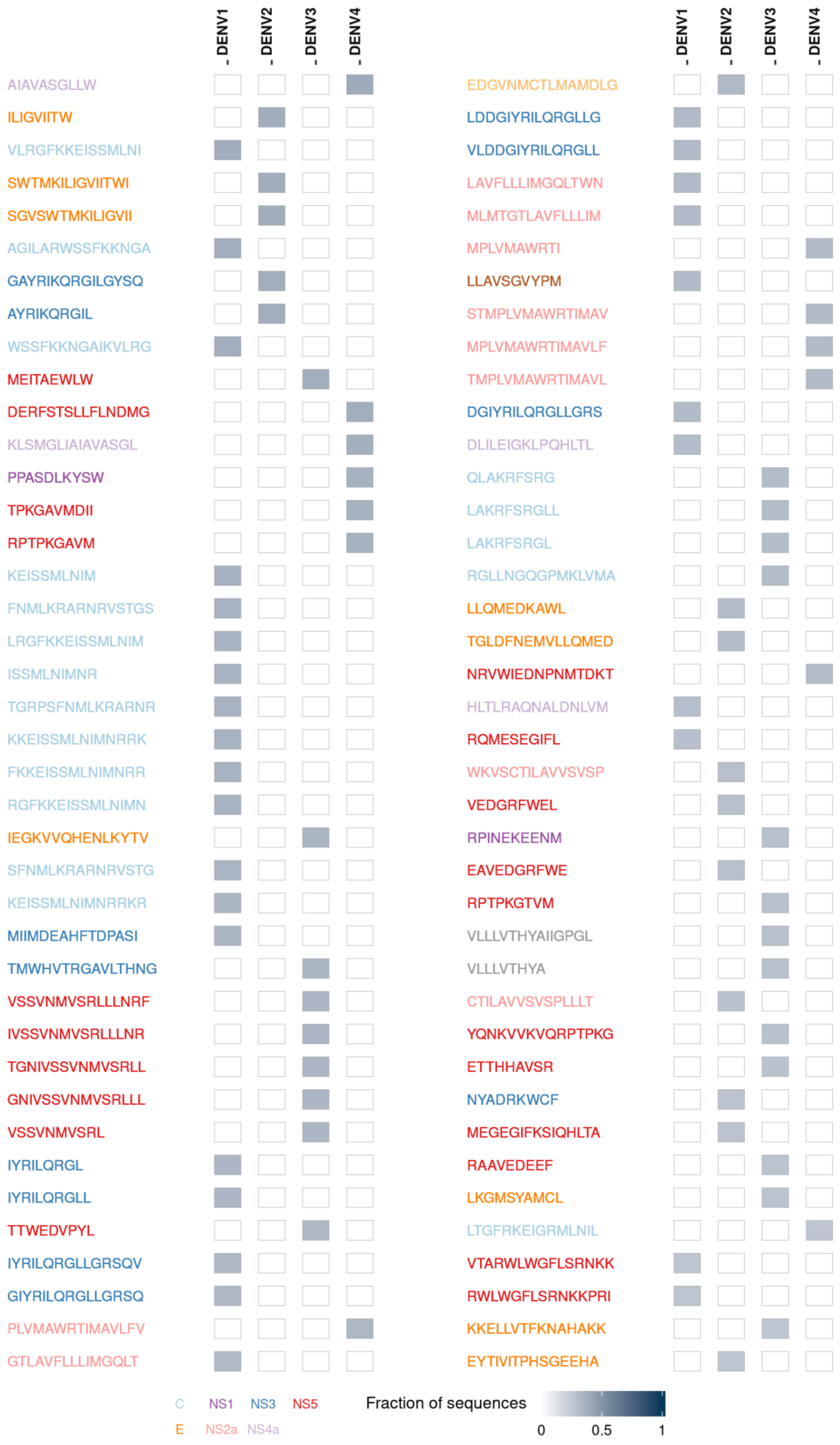
DENV1
DENV2
DENV3
DENV4
AIAVASGLLW
ILIGVIITW
VLRGFKKEISSMLNI
SWTMKILIGVIITWI
SGVSWTMKILIGVII
AGILARWSSFKKNGA
GAYRIKQRGILGYSQ
AYRIKQRGIL
WSSFKKNGAIKVLRG
MEITAEWLW
DERFSTSLLFLNDMG
KLSMGLIAIAVASGL
PPASDLKYSW
TPKGAVMDII
RPTPKGAVM
KEISSMLNIM
FNMLKRARNRVSTGS
LRGFKKEISSMLNIM
ISSMLNIMNR
TGRPSFNMLKRARNR
KKEISSMLNIMNRRK
FKKEISSMLNIMNRR
RGFKKEISSMLNIMN
IEGKVVQHENLKYTV
SFNMLKRARNRVSTG
KEISSMLNIMNRRKR
MIIMDEAHFTDPASI
TMWHVTRGAVLTHNG
VSSVNMVSRLLLNRF
IVSSVNMVSRLLLNR
TGNIVSSVNMVSRLL
GNIVSSVNMVSRLLL
VSSVNMVSRL
IYRILQRGL
IYRILQRGLL
TTWEDVPYL
IYRILQRGLLGRSQV
GIYRILQRGLLGRSQ
PLVMAWRTIMAVLFV
GTLAVFLLLIMGQLT
EDGVNMCTLMAMDLG
LDDGIYRILQRGLLG
VLDDGIYRILQRGLL
LAVFLLLIMGQLTWN
MLMTGTLAVFLLLIM
MPLVMAWRTI
LLAVSGVYPM
STMPLVMAWRTIMAV
MPLVMAWRTIMAVLF
TMPLVMAWRTIMAVL
DGIYRILQRGLLGRS
DLILEIGKLPQHLTL
QLAKRFSRG
LAKRFSRGLL
LAKRFSRGL
RGLLNGQGPMKLVMA
LLQMEDKAWL
TGLDFNEMVLLQMED
NRVWIEDNPNMTDKT
HLTLRAQNALDNLVM
RQMESEGIFL
WKVSCTILAVVSVSP
VEDGRFWEL
RPINEKEENM
EAVEDGRFWE
RPTPKGTVM
VLLLVTHYAIIGPGL
VLLLVTHYA
CTILAVVSVSPLLLT
YQNKVVKVQRPTPKG
ETTHHAVSR
NYADRKWCF
MEGEGIFKSIQHLTA
RAAVEDEEF
LKGMSYAMCL
LTGFRKEIGRMLNIL
VTARWLWGFLSRNKK
RWLWGFLSRNKKPRI
KKELLVTFKNAHAKK
EYTIVITPHSGEEHA
C
NS1
NS3
NS5
E
NS2a
NS4a
Fraction of sequences
0
0.5
1

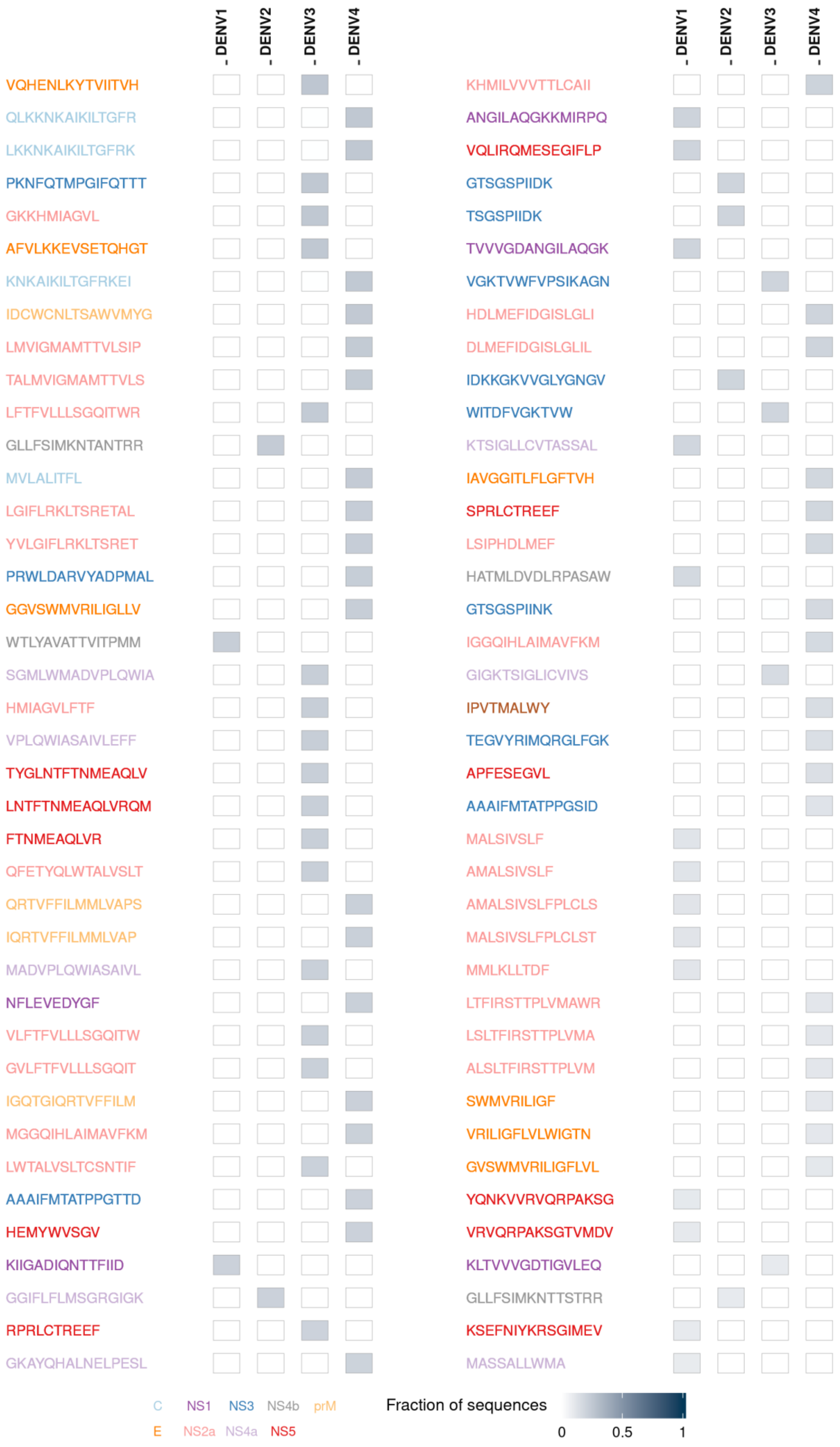
DENV1
DENV2
DENV3
DENV4
VQHENLKYTVIITVH
QLKKNKAIKILTGFR
LKKNKAIKILTGFRK
PKNFQTMPGIFQTTT
GKKHMIAGVL
AFVLKKEVSETQHGT
KNKAIKILTGFRKEI
IDCWCNLTSAWVMYG
LMVIGMAMTTVLSIP
TALMVIGMAMTTVLS
LFTFVLLLSGQITWR
GLLFSIMKNTANTRR
MVLALITFL
LGIFLRKLTSRETAL
YVLGIFLRKLTSRET
PRWLDARVYADPMAL
GGVSWMVRILIGLLV
WTLYAVATTVITPMM
SGMLWMADVPLQWIA
HMIAGVLFTF
VPLQWIASAIVLEFF
TYGLNTFTNMEAQLV
LNTFTNMEAQLVRQM
FTNMEAQLVR
QFETYQLWTALVSLT
QRTVFFILMMLVAPS
IQRTVFFILMMLVAP
MADVPLQWIASAIVL
NFLEVEDYGF
VLFTFVLLLSGQITW
GVLFTFVLLLSGQIT
IGQTGIQRTVFFILM
MGGQIHLAIMAVFKM
LWTALVSLTCSNTIF
AAAIFMTATPPGTTD
HEMYWVSGV
KIIGADIQNTTFIID
GGIFLFLMSGRGIGK
RPRLCTREEF
GKAYQHALNELPESL
KHMILVVVTTLCAII
ANGILAQGKKMIRPQ
VQLIRQMESEGIFLP
GTSGSPIIDK
TSGSPIIDK
TVVVGDANGILAQGK
VGKTVWFVPSIKAGN
HDLMEFIDGISLGLI
DLMEFIDGISLGLIL
IDKKGKVVGLYGNGV
WITDFVGKTVW
KTSIGLLCVTASSAL
IAVGGITLFLGFTVH
SPRLCTREEF
LSIPHDLMEF
HATMLDVDLRPASAW
GTSGSPIINK
IGGQIHLAIMAVFKM
GIGKTSIGLICVIVS
IPVTMALWY
TEGVYRIMQRGLFGK
APFESEGVL
AAAIFMTATPPGSID
MALSIVSLF
AMALSIVSLF
AMALSIVSLFPLCLS
MALSIVSLFPLCLST
MMLKLLTDF
LTFIRSTTPLVMAWR
LSLTFIRSTTPLVMA
ALSLTFIRSTTPLVM
SWMVRILIGF
VRILIGFLVLWIGTN
GVSWMVRILIGFLVL
YQNKVVRVQRPAKSG
VRVQRPAKSGTVMDV
KLTVVVGDTIGVLEQ
GLLFSIMKNTTSTRR
KSEFNIYKRSGIMEV
MASSALLWMA
C
NS1
NS3
NS4b
prM
E
NS2a
NS4a
NS5
Fraction of sequences
0
0.5
1

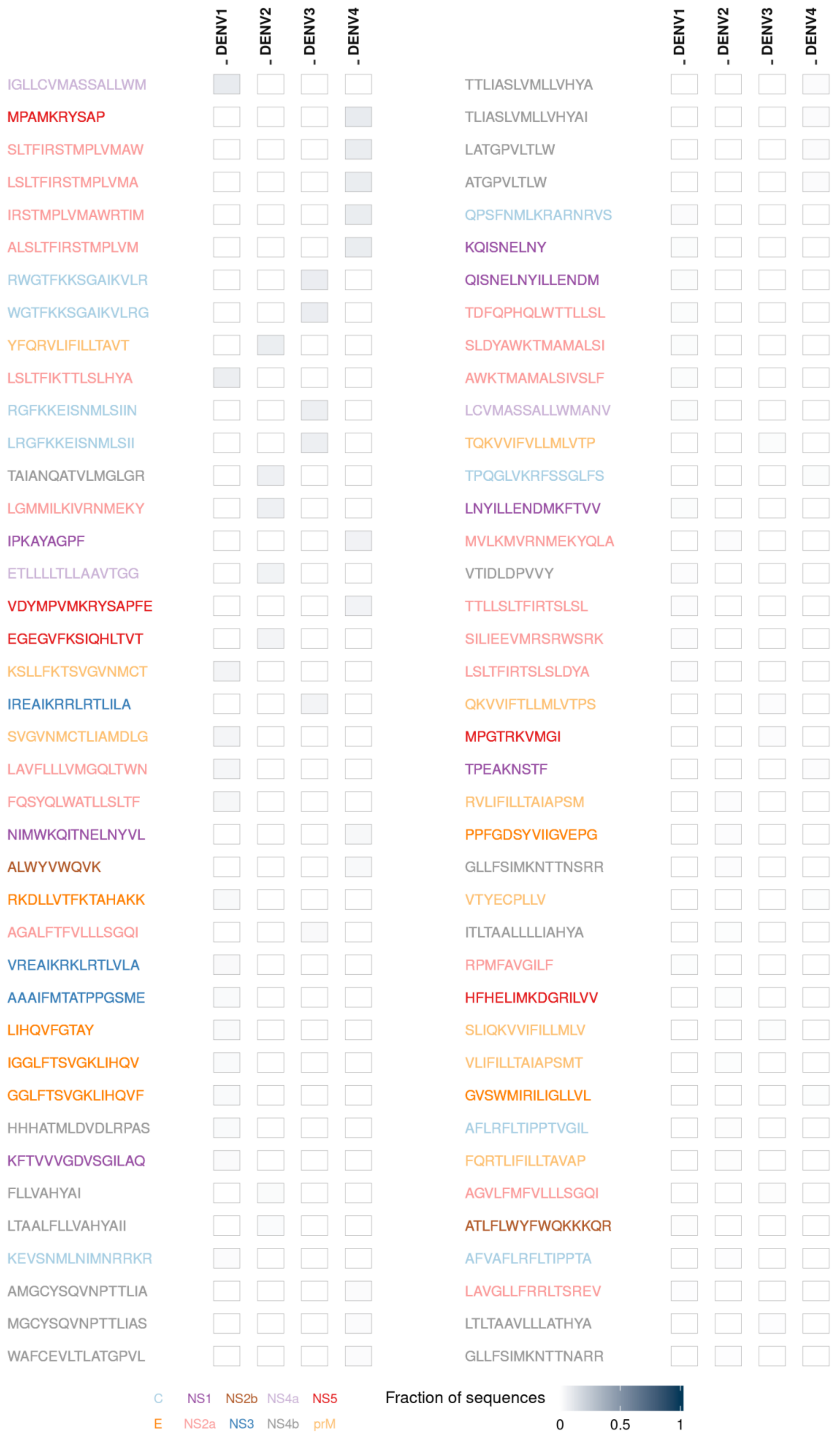
DENV1
DENV2
DENV3
DENV4
IGLLCVMASSALLWM
MPAMKRYSAP
SLTFIRSTMPLVMAW
LSLTFIRSTMPLVMA
IRSTMPLVMAWRTIM
ALSLTFIRSTMPLVM
RWGTFKKSGAIKVLR
WGTFKKSGAIKVLRG
YFQRVLIFILLTAVT
LSLTFIKTTLSLHYA
RGFKKEISNMLSIIN
LRGFKKEISNMLSII
TAIANQATVLMGLGR
LGMMILKIVRNMEKY
IPKAYAGPF
ETLLLLTLLAAVTGG
VDYMPVMKRYSAPFE
EGEGVFKSIQHLTVT
KSLLFKTSVGVNMCT
IREAIKRRLRTLILA
SVGVNMCTLIAMDLG
LAVFLLLVMGQLTWN
FQSYQLWATLLSLTF
NIMWKQITNELNYVL
ALWYVWQVK
RKDLLVTFKTAHAKK
AGALFTFVLLLSGQI
VREAIKRKLRTLVLA
AAAIFMTATPPGSME
LIHQVFGTAY
IGGLFTSVGKLIHQV
GGLFTSVGKLIHQVF
HHHATMLDVDLRPAS
KFTVVVGDVSGILAQ
FLLVAHYAI
LTAALFLLVAHYAII
KEVSNMLNIMNRRKR
AMGCYSQVNPTTLIA
MGCYSQVNPTTLIAS
WAFCEVLTLATGPVL
TTLIASLVMLLVHYA
TLIASLVMLLVHYAI
LATGPVLTLW
ATGPVLTLW
QPSFNMLKRARNRVS
KQISNELNY
QISNELNYILLENDM
TDFQPHQLWTTLLSL
SLDYAWKTMAMALSI
AWKTMAMALSIVSLF
LCVMASSALLWMANV
TQKVVIFVLLMLVTP
TPQGLVKRFSSGLFS
LNYILLENDMKFTVV
MVLKMVRNMEKYQLA
VTIDLDPVVY
TTLLSLTFIRTSLSL
SILIEEVMRSRWSRK
LSLTFIRTSLSLDYA
QKVVIFTLLMLVTPS
MPGTRKVMGI
TPEAKNSTF
RVLIFILLTAIAPSM
PPFGDSYVIIGVEPG
GLLFSIMKNTTNSRR
VTYECPLLV
ITLTAALLLLIAHYA
RPMFAVGILF
HFHELIMKDGRILVV
SLIQKVVIFILLMLV
VLIFILLTAIAPSMT
GVSWMIRILIGLLVL
AFLRFLTIPPTVGIL
FQRTLIFILLTAVAP
AGVLFMFVLLLSGQI
ATLFLWYFWQKKKQR
AFVAFLRFLTIPPTA
LAVGLLFRRLTSREV
LTLTAAVLLLATHYA
GLLFSIMKNTTNARR
C
NS1
NS2b
NS4a
NS5
E
NS2a
NS3
NS4b
prM
Fraction of sequences
0
0.5
1

S3 Fig. Conservation of the 1,768 T cell epitope sequences across the DENV serotypes. Cells adjacent to each epitope represents its conservation within each DENV serotype. The conservation level (i.e., fraction of sequences in which a given epitope was exactly mapped) for an epitope within a serotype was determined by mapping it onto all the corresponding protein sequences for that serotype, as shown in Fig 2B. All epitopes are shown here in descending order (top to bottom then left to right) of their mean conservation across the serotypes and colored according to the protein from which they are derived.
