# Supplementary material for: Cross-serotypically conserved epitope recommendations for a universal T cell-based dengue vaccine
Source: PLoS Negl Trop Dis. 2020 Sep 21;14(9):e0008676. doi: 10.1371/journal.pntd.0008676 (PMC7529213; doi:10.1371/journal.pntd.0008676)
Supplement: S4 Fig — Minimum conservation of epitopes across sets of 3 out of 4 DENV serotypes. The conservation for each epitope within each serotype of DENV was determined after mapping the epitopes onto the corresponding protein sequences, as shown in Fig 2B. A threshold of 0.9 was used that resulted in the set of 55 top epitopes (i.e., epitopes that mapped exactly onto at least 90% of sequences in at least 3 of the 4 DENV serotypes were selected), shown in Fig 3. The histograms show that the selection of epitopes is robust to the choice of threshold. (PDF) [file pntd.0008676.s004.pdf]

Supplementary Figure

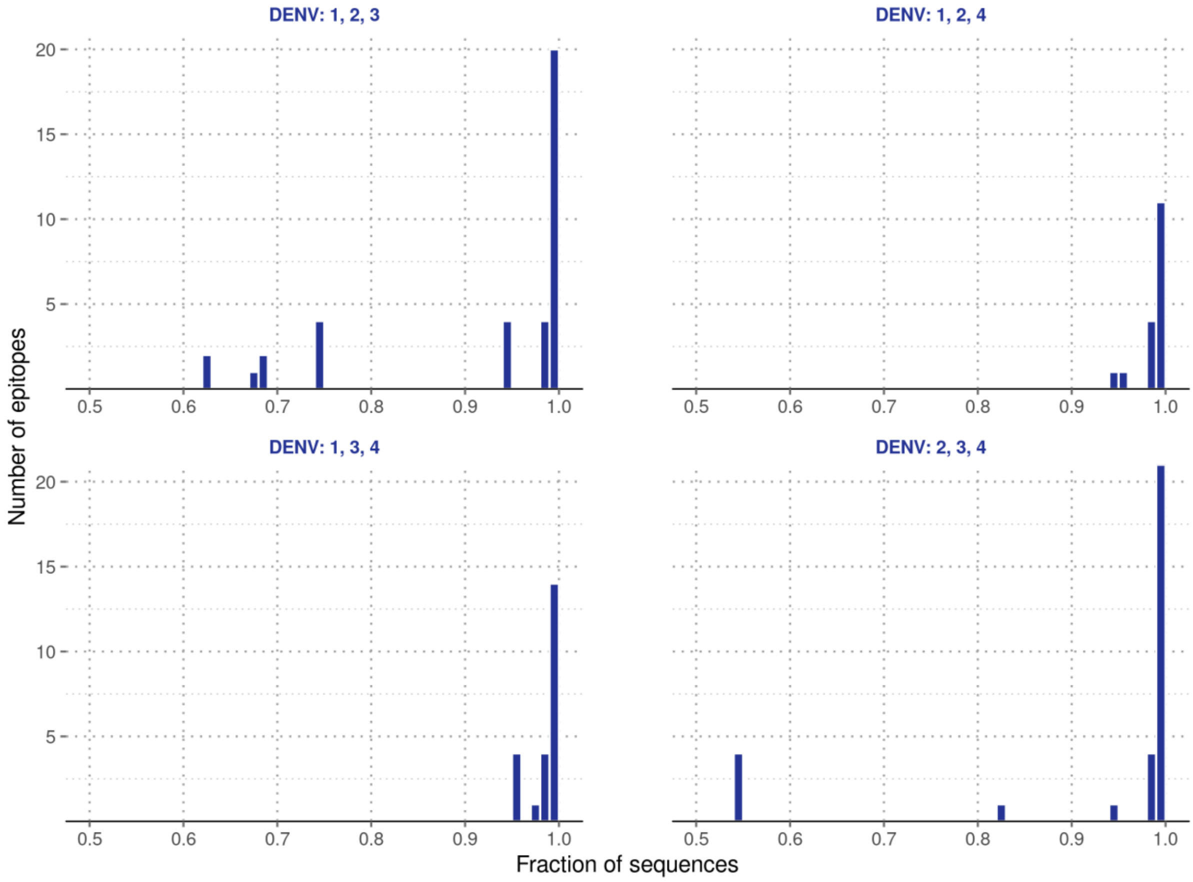

S4 Fig. Histogram of minimum conservation of epitopes among 3 DENV serotypes. Minimum conservation of epitopes across sets of 3 out of 4 DENV serotypes. The conservation for each epitope within each serotype of DENV was determined after mapping the epitopes onto the corresponding protein sequences, as shown in Fig 2B. A threshold of 0.9 was used that resulted in the set of 55 top epitopes (i.e., epitopes that mapped exactly onto at least 90% of sequences in at least 3 of the 4 DENV serotypes were selected), shown in Fig 3. The histograms show that the selection of epitopes is robust to the choice of threshold.
