# Supplementary material for: Cross-serotypically conserved epitope recommendations for a universal T cell-based dengue vaccine
Source: PLoS Negl Trop Dis. 2020 Sep 21;14(9):e0008676. doi: 10.1371/journal.pntd.0008676 (PMC7529213; doi:10.1371/journal.pntd.0008676)
Supplement: S5 Fig — The NS5 dimer structure (PDB: 5CCV) proposed in [53] is shown where one monomer is colored blue and the other green. The residues of two top NS5 epitopes (GPGHEEPIPM and KVRKDIPQW) that are involved in the inter-dimer interface are shown as spheres. (PDF) [file pntd.0008676.s005.pdf]

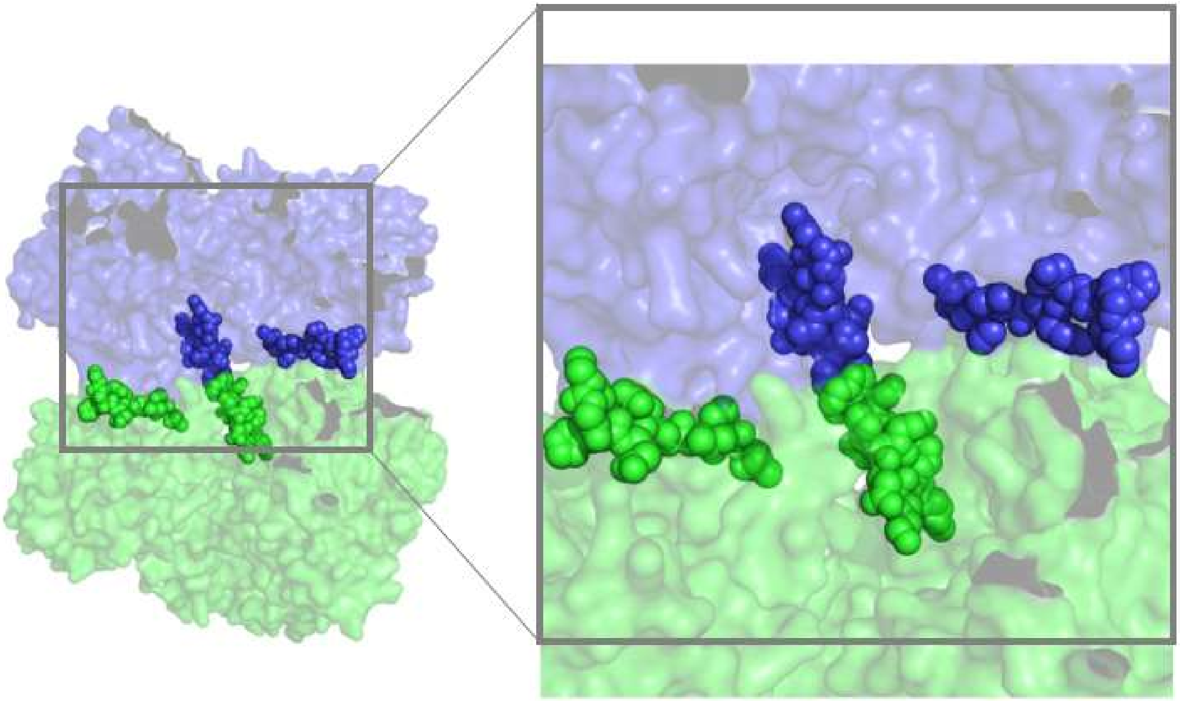

S5 Fig. Some top epitopes are putatively involved in the inter-dimer interface in the quaternary structure proposed for DENV NS5. The NS5 dimer structure (PDB: 5CCV) proposed in [53] is shown where one monomer is colored blue and the other green. The residues of two top NS5 epitopes (GPGHEEPIPM and KVRKDIPQW) that are involved in the inter-dimer interface are shown as spheres.
