# Supplementary material for: Cross-serotypically conserved epitope recommendations for a universal T cell-based dengue vaccine
Source: PLoS Negl Trop Dis. 2020 Sep 21;14(9):e0008676. doi: 10.1371/journal.pntd.0008676 (PMC7529213; doi:10.1371/journal.pntd.0008676)
Supplement: S6 Fig — (Left panel) Top 51 epitopes, selected from the set of top 55 DENV epitopes (Fig 3) that had at least one HLA allele associated with 4-digit resolution. The cells adjacent to each epitope represent its conservation within each DENV serotype. The individual population coverage of each epitope (Middle panel) and the accumulated population coverage of the combination of epitopes (Right panel) was calculated based on the associated HLA alleles. The epitopes are ranked in increasing order of the accumulated coverage which reached the maximum of 99.23% with top 17 epitopes. The remaining epitopes are ordered in decreasing order of their mean conservation (Fig 3). Epitopes are colored according to the protein from which they are derived while the HLA alleles are colored according to their class restriction. Number of epitopes derived from each protein is shown within parentheses at the bottom of left panel. (PDF) [file pntd.0008676.s006.pdf]

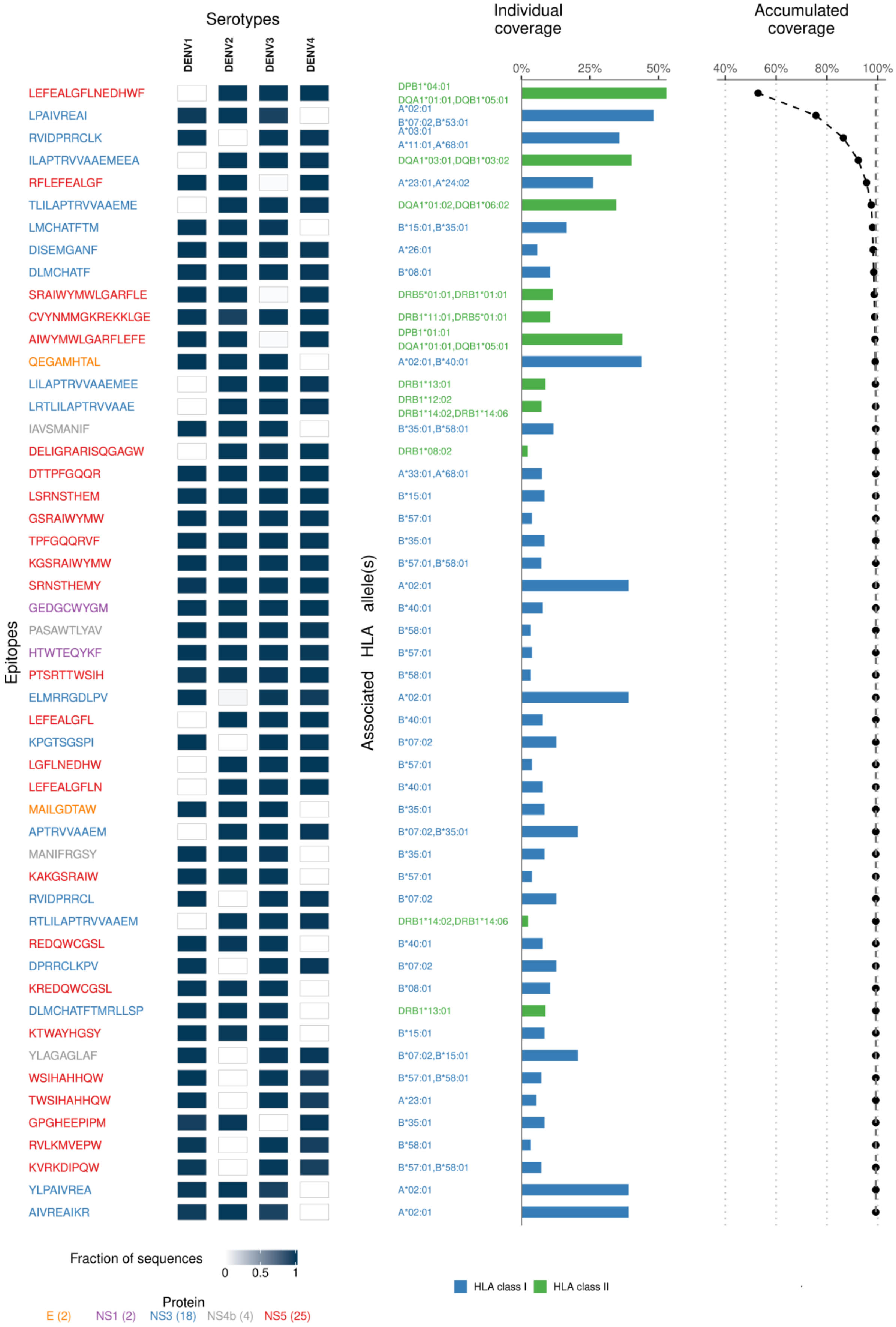

Serotypes
DENV1
DENV2
DENV3
DENV4
Individual coverage
Accumulated coverage
0%
25%
50%
40%
60%
80%
100%
Epitopes
Associated HLA allele(s)
LEFEALGFLNEDHWF
LPAIVREAI
RVIDPRRCLK
ILAPTRVVAAEMEEA
RFLEFEALGF
TLILAPTRVVAAEME
LMCHATFTM
DISEMGANF
DLMCHATF
SRAIWYMWLGARFLE
CVYNMMGKREKKLGE
AIWYMWLGARFLEFE
QEGAMHTAL
LILAPTRVVAAEMEE
LRTLILAPTRVVAAE
IAVSMANIF
DELIGRARISQGAGW
DTTPFGQQR
LSRNSTHEM
GSRAIWYMW
TPFGQQRVF
KGSRAIWYMW
SRNSTHEMY
GEDGCWYGM
PASAWTLYAV
HTWTEQYKF
PTSRTTWSIH
ELMRRGDLPV
LEFEALGFL
KPGTSGSPI
LGFLNEDHW
LEFEALGFLN
MAILGDTAW
APTRVVAAEM
MANIFRGSY
KAKGSRAIW
RVIDPRRCL
RTLILAPTRVVAAEM
REDQWCGSL
DPRRCLKPV
KREDQWCGSL
DLMCHATFTMRLLSP
KTWAYHGSY
YLAGAGLAF
WSIHAHHQW
TWSIHAHHQW
GPGHEEPIPM
RVLKMVEPW
KVRKDIPQW
YLPAIVREA
AIVREAIKR
DPB1*04:01
DQA1*01:01,DQB1*05:01
A*02:01
B*07:02,B*53:01
A*03:01
A*11:01,A*68:01
DQA1*03:01,DQB1*03:02
A*23:01,A*24:02
DQA1*01:02,DQB1*06:02
B*15:01,B*35:01
A*26:01
B*08:01
DRB5*01:01,DRB1*01:01
DRB1*11:01,DRB5*01:01
DPB1*01:01
DQA1*01:01,DQB1*05:01
A*02:01,B*40:01
DRB1*13:01
DRB1*12:02
DRB1*14:02,DRB1*14:06
B*35:01,B*58:01
DRB1*08:02
A*33:01,A*68:01
B*15:01
B*57:01
B*35:01
B*57:01,B*58:01
A*02:01
B*40:01
B*58:01
B*57:01
B*58:01
A*02:01
B*40:01
B*07:02
B*57:01
B*40:01
B*35:01
B*07:02,B*35:01
B*35:01
B*57:01
B*07:02
DRB1*14:02,DRB1*14:06
B*40:01
B*07:02
B*08:01
DRB1*13:01
B*15:01
B*07:02,B*15:01
B*57:01,B*58:01
A*23:01
B*35:01
B*58:01
B*57:01,B*58:01
A*02:01
A*02:01
Fraction of sequences
0
0.5
1
Protein
E (2)
NS1 (2)
NS3 (18)
NS4b (4)
NS5 (25)
HLA class I
HLA class II

S6 Fig. Global population coverage for the identified top DENV T cell epitopes. (A) Top 51 epitopes, selected from the set of top 55 DENV epitopes (Fig. 3) that had at least one HLA allele associated with 4-digit resolution (Left panel). The cells adjacent to each epitope represent its conservation within each DENV serotype. The individual population coverage (Middle panel) of each epitope and the accumulated population coverage (Right panel) of the combination of epitopes was calculated based on the associated HLA alleles. The epitopes are ranked in increasing order of the accumulated coverage which reached the maximum of 99.23% with top 17 epitopes. The remaining epitopes are ordered in decreasing order of their mean conservation (Fig. 3). Epitopes are colored according to the protein from which they are derived while the HLA alleles are colored according to their class restriction. Number of epitopes derived from each protein is shown within parentheses at the bottom of left panel.
