# Supplementary material for: Cross-serotypically conserved epitope recommendations for a universal T cell-based dengue vaccine
Source: PLoS Negl Trop Dis. 2020 Sep 21;14(9):e0008676. doi: 10.1371/journal.pntd.0008676 (PMC7529213; doi:10.1371/journal.pntd.0008676)
Supplement: S7 Fig — (A) Thailand, (B) Philippines and (C) Brazil. For all (A-C): (Left panel) Proposed immunogens comprising epitopes selected from the set of top 55 DENV epitopes (Fig 3), that maximized the country-specific population coverages. The cells adjacent to each epitope represent its conservation within each DENV serotype. (Middle panel) The individual population coverage of each epitope and (right panel) the accumulated population coverage calculated based on the associated HLA alleles. The epitopes are ranked in increasing order of the accumulated coverage which reached the maximum of: (A) 98.25%, (B) 96.73%, and (C) 98.8%. Incorporating more top epitopes within these immunogens did not result in further increase of coverage. Epitopes are colored according to the protein from which they are derived while the HLA alleles are colored according to their class restriction. Number of epitopes within the immunogens derived from each protein is shown within parentheses at the bottom of left panel in (A-C) respectively. (PDF) [file pntd.0008676.s007.pdf]

Supplementary Figure

Thailand

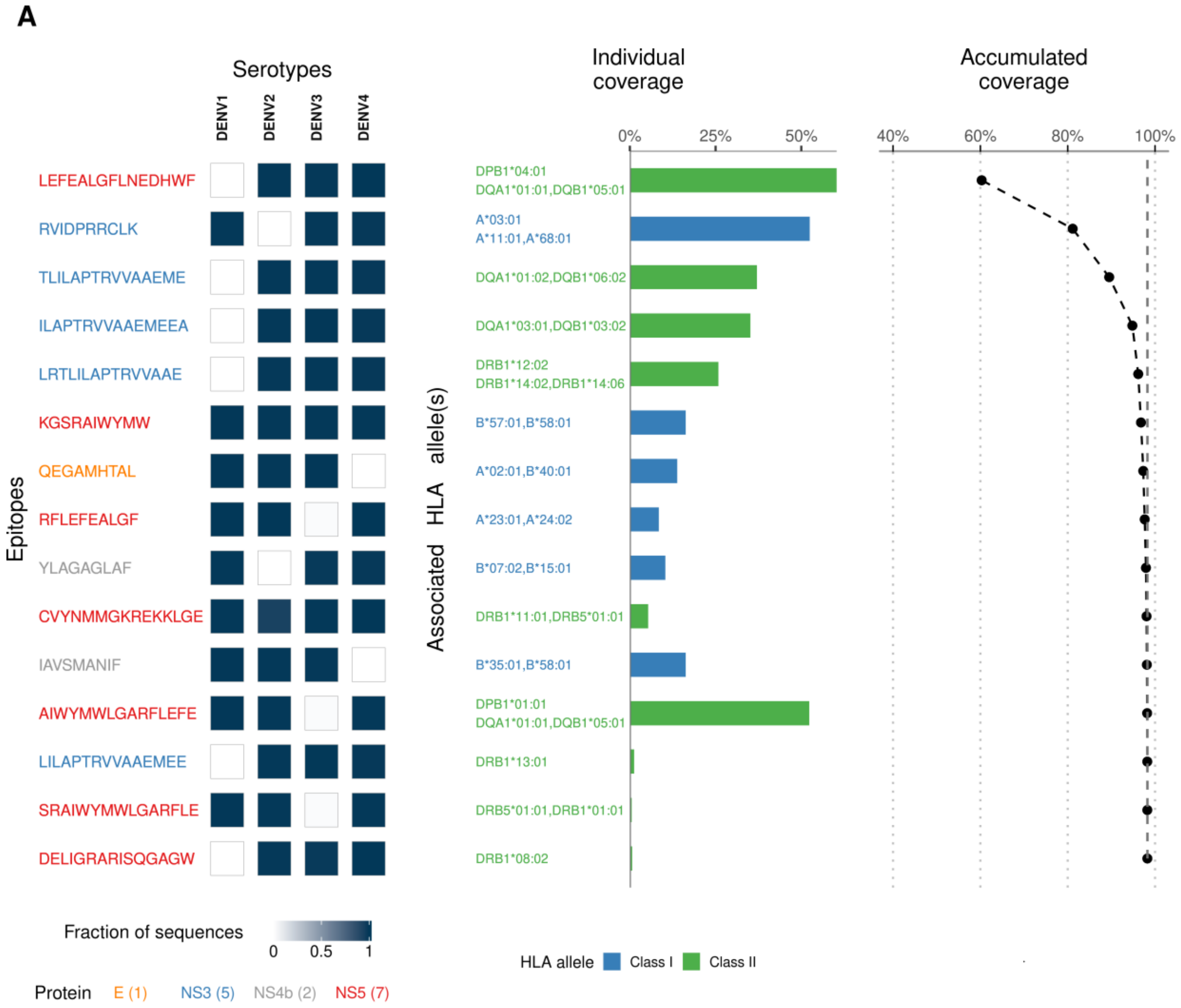

Philippines

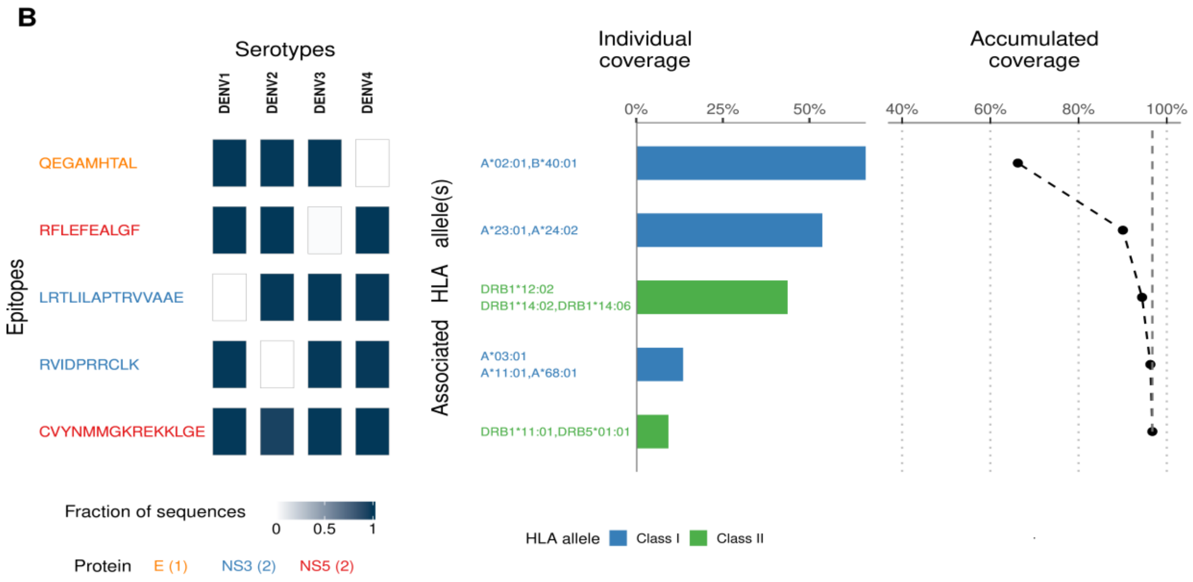

Brazil

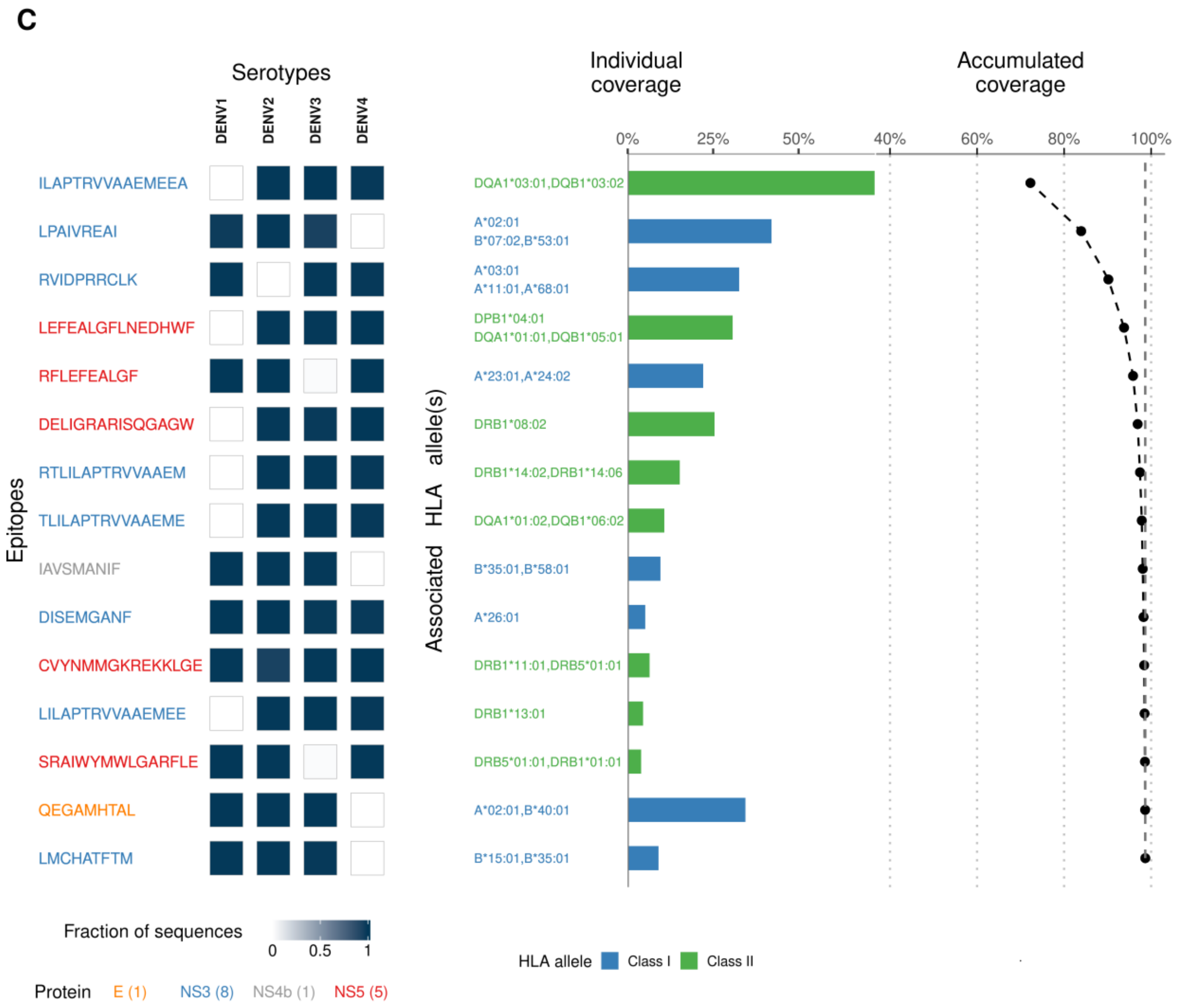

S7 Fig. Proposed immunogens for country-specific populations: (A) Thailand, **(B)** Philippines and **(C)** Brazil. For all (A-C): (Left panel) Proposed immunogens comprising epitopes selected from the set of top 55 DENV epitopes (Fig. 3), that maximized the country-specific population coverages. The cells adjacent to each epitope represent its conservation within each DENV serotype. (Middle panel) The individual population coverage of each epitope and (right panel) the accumulated population coverage calculated based on the associated HLA alleles. The epitopes are ranked in increasing order of the accumulated coverage which reached the maximum of: (A) 98.25%, (B) 96.73%, and (C) 98.8%. Incorporating more top epitopes within these immunogens did not result in further increase of coverage. Epitopes are colored according to the protein from which they are derived while the HLA alleles are colored according to their class restriction. Number of epitopes within the immunogens derived from each protein is shown within parentheses at the bottom of left panel in (A-C) respectively.
