# Supplementary material for: Cross-serotypically conserved epitope recommendations for a universal T cell-based dengue vaccine
Source: PLoS Negl Trop Dis. 2020 Sep 21;14(9):e0008676. doi: 10.1371/journal.pntd.0008676 (PMC7529213; doi:10.1371/journal.pntd.0008676)
Supplement: S9 Fig — Of the 213 experimentally-determined HLA-transgenic mice epitopes, 9 mapped exactly onto at least 90% of sequences in at least 3 of the 4 DENV serotypes. Five of these epitopes–APTRVVAAEM, ELMRRGDLPV, RVIDPRRCL, SRAIWYMWLGARFLE, and LPAIVREAI–were also reported from human hosts (Fig 3). Cells adjacent to each epitope represents its conservation within each DENV serotype. The conservation of an epitope within a serotype was determined using the exact mapping procedure (Fig 2B). All epitopes are shown here in descending order (top to bottom) of their mean conservation across the serotypes and colored according to the protein from which they are derived. (PDF) [file pntd.0008676.s009.pdf]

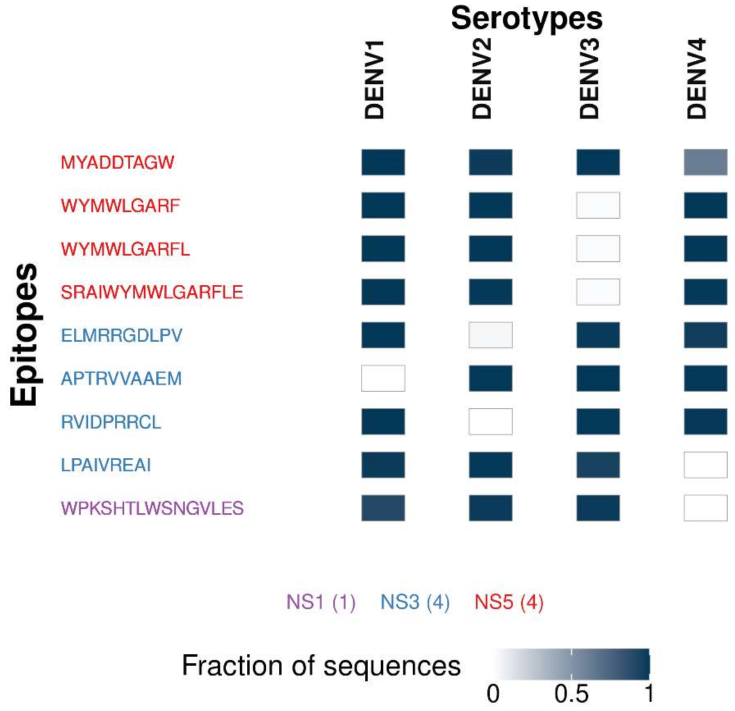

S9 Fig. Cross-serotypically-conserved DENV T cell epitopes from HLA-transgenic mice. Of the 213 experimentally-determined HLA-transgenic mice epitopes, 9 mapped exactly onto at least 90% of sequences in at least 3 of the 4 DENV serotypes. Five of these epitopes – APTRVVAAEM, ELMRRGDLPV, RVIDPRRCL, SRAIWYMWLGARFLE, and LPAIVREAI – were also reported from human hosts (Fig. 3). Cells adjacent to each epitope represents its conservation within each DENV serotype. The conservation of an epitope within a serotype was determined using the exact mapping procedure (Fig. 2B). All epitopes are shown here in descending order (top to bottom) of their mean conservation across the serotypes and colored according to the protein from which they are derived. (PDF)
