# Supplementary material for: Cross-serotypically conserved epitope recommendations for a universal T cell-based dengue vaccine
Source: PLoS Negl Trop Dis. 2020 Sep 21;14(9):e0008676. doi: 10.1371/journal.pntd.0008676 (PMC7529213; doi:10.1371/journal.pntd.0008676)
Supplement: S3 Table — (PDF) [file pntd.0008676.s012.pdf]

Identified cross-serotypically conserved epitopes (Fig. 3) reported as immunodominant in previous studies	Reference
APTRVVAAEM, DTTPFGQQR	[1]
APTRVVAAEM, ELMRRGDLPV, LPAIVREAI, RVIDPRRCL	[2]
AIVREAIKR, APTRVVAAEM, RVIDPRRCLK, YLPAIVREA, ILAPTRVVAAEMEEA, LAPTRVVAAEME, LPAIVREAI, RVIDPRRCL, IAVSMANIF, MANIFRGSY, DTTPFGQQR, KAKGSRAIW, TPFGQQRVF	[3]

References

1. de Alwis R, Bangs DJ, Angelo MA, Cerpas C, Fernando A, Sidney J, et al. Immunodominant dengue virus-specific CD8 + T cell responses are associated with a memory PD-1 + phenotype. Perlman S, editor. J Virol. 2016;90: 4771–4779.

2. Weiskopf D, Angelo MA, de Azeredo EL, Sidney J, Greenbaum JA, Fernando AN, et al. Comprehensive analysis of dengue virus-specific responses supports an HLA-linked protective role for CD8+ T cells. Proc Natl Acad Sci U S A. 2013;110: E2046-53.

3. Tian Y, Grifoni A, Sette A, Weiskopf D. Human T cell response to dengue virus infection. Front Immunol. 2019;10: 1–9.
