# Supplementary material for: Cross-serotypically conserved epitope recommendations for a universal T cell-based dengue vaccine
Source: PLoS Negl Trop Dis. 2020 Sep 21;14(9):e0008676. doi: 10.1371/journal.pntd.0008676 (PMC7529213; doi:10.1371/journal.pntd.0008676)
Supplement: S4 Table — (PDF) [file pntd.0008676.s013.pdf]

Vaccine	Identified cross-serotypically conserved epitopes (Fig. 3) against which T cell responses were reported in naïve individuals following vaccination
Monovalent LAV/ Tetravalent LAV (TV003) [1]	HTWTEQYKF, APTRVVAAEM, DPRRCLKPV, KPGTSGSPI, RVIDPRRCLK, DISEMGANF, IAVSMANIF, MANIFRGSY, KAKGSRAIW, RFLEFEALGF, KVRKDIPQW, TWSIHAHHQW, TPFGQQRVF
Tetravalent LAV (TK-003) [2]	AIVREAIKR, APTRVVAAEM, YLPAIVREA, LPAIVREAI, LRTLILAPTRVVAAE, RTLILAPTRVVAAEM, IAVSMANIF, MANIFRGSY, KTWAYHGSY

References

1. Weiskopf D, Angelo MA, Bangs DJ, Sidney J, Paul S, Peters B, et al. The human CD8+ T cell responses induced by a live attenuated tetravalent dengue vaccine are directed against highly conserved epitopes. Diamond MS, editor. J Virol. 2015;89: 120–128.

2. Waickman AT, Friberg H, Gargulak M, Kong A, Polhemus M, Endy T, et al. Assessing the diversity and stability of cellular immunity generated in response to the candidate live-attenuated dengue virus vaccine TAK-003. Front Immunol. 2019;10: 1–13.
