# Supplementary material for: Cross-serotypically conserved epitope recommendations for a universal T cell-based dengue vaccine
Source: PLoS Negl Trop Dis. 2020 Sep 21;14(9):e0008676. doi: 10.1371/journal.pntd.0008676 (PMC7529213; doi:10.1371/journal.pntd.0008676)
Supplement: S5 Table — Overlapping residues are indicated as red. (PDF) [file pntd.0008676.s014.pdf]

Conserved regions/peptides reported in previous works that overlap with cross-serotypically conserved epitopes identified in this study			Reference
LGSQEGAMHTALTGA	NFKADRVIDPRRCLK	MVTQMAMTDTTPFGQQR	[1]
TRKYLPAIVREAIKR	FVVTTDISEMGANFK	WLGARYLEFEALGFLNE	
LPAIVREAIKRRLRT	TDISEMGANFKADRV	GSCVYNMMGKREKKLGE	
AIALDFKPGTSGSPI	DENPYKTWAYHGSYEVK	YLGKREDQWCGSLIGLT	
VHTWTEQYKFQ	PASAWTLYAVATT	AKGSRAIWYMWLGAR	[2]
GEDGCWYGMEIRP	PLSRNSTHEMYW	PTSRTTWSIHA	
EIVDLMCHATFT	AMTDTTPFGQQRVFKEKVDTRT		
VVTTDISEMGANF	CVYNMMGKREKKLGEFG		

References

1. Nascimento EJM, Mailliard RB, Khan AM, Sidney J, Sette A, Guzman N, et al. Identification of conserved and HLA promiscuous DENV3 T-cell epitopes. PLoS Negl Trop Dis. 2013;7.

2. Khan AM, Miotto O, Nascimento EJM, Srinivasan KN, Heiny AT, Zhang GL, et al. Conservation and variability of dengue virus proteins: Implications for vaccine design. PLoS Negl Trop Dis. 2008;2.
