# Supplementary material for: Cross-serotypically conserved epitope recommendations for a universal T cell-based dengue vaccine
Source: PLoS Negl Trop Dis. 2020 Sep 21;14(9):e0008676. doi: 10.1371/journal.pntd.0008676 (PMC7529213; doi:10.1371/journal.pntd.0008676)
Supplement: S2 File — Immune response data includes response frequency, and qualitative and quantitative binding reported in IEDB for each identified epitope. The response frequency percentile for each epitope, that we computed using the response frequencies of all available DENV epitopes, is also included in this table. (HTML) [file pntd.0008676.s016.html]

S2 File: Cross-serotypic conservation profiles and immune response data of the top 55 conserved DENV T cell epitopes identified in this study (Fig. 3)
