# Supplementary material for: Cross-serotypically conserved epitope recommendations for a universal T cell-based dengue vaccine
Source: PLoS Negl Trop Dis. 2020 Sep 21;14(9):e0008676. doi: 10.1371/journal.pntd.0008676 (PMC7529213; doi:10.1371/journal.pntd.0008676)
Supplement: S3 File — (HTML) [file pntd.0008676.s017.html]

S3 File: Cross-serotypic conservation profiles of 213 DENV T cell epitopes experimentally-determined from HLA-transgenic mice
