# Supplementary material for: Cross-serotypically conserved epitope recommendations for a universal T cell-based dengue vaccine
Source: PLoS Negl Trop Dis. 2020 Sep 21;14(9):e0008676. doi: 10.1371/journal.pntd.0008676 (PMC7529213; doi:10.1371/journal.pntd.0008676)
Supplement: S8 Fig — The quantitative measure, IC50 values, of epitope-MHC binding was extracted from positive MHC assays corresponding to these epitopes in the IEDB database. Color of the cells represent the strength of the binding affinity. White cells represent epitope-HLA complexes for which no binding measurements were reported. (PDF) [file pntd.0008676.s008.pdf]

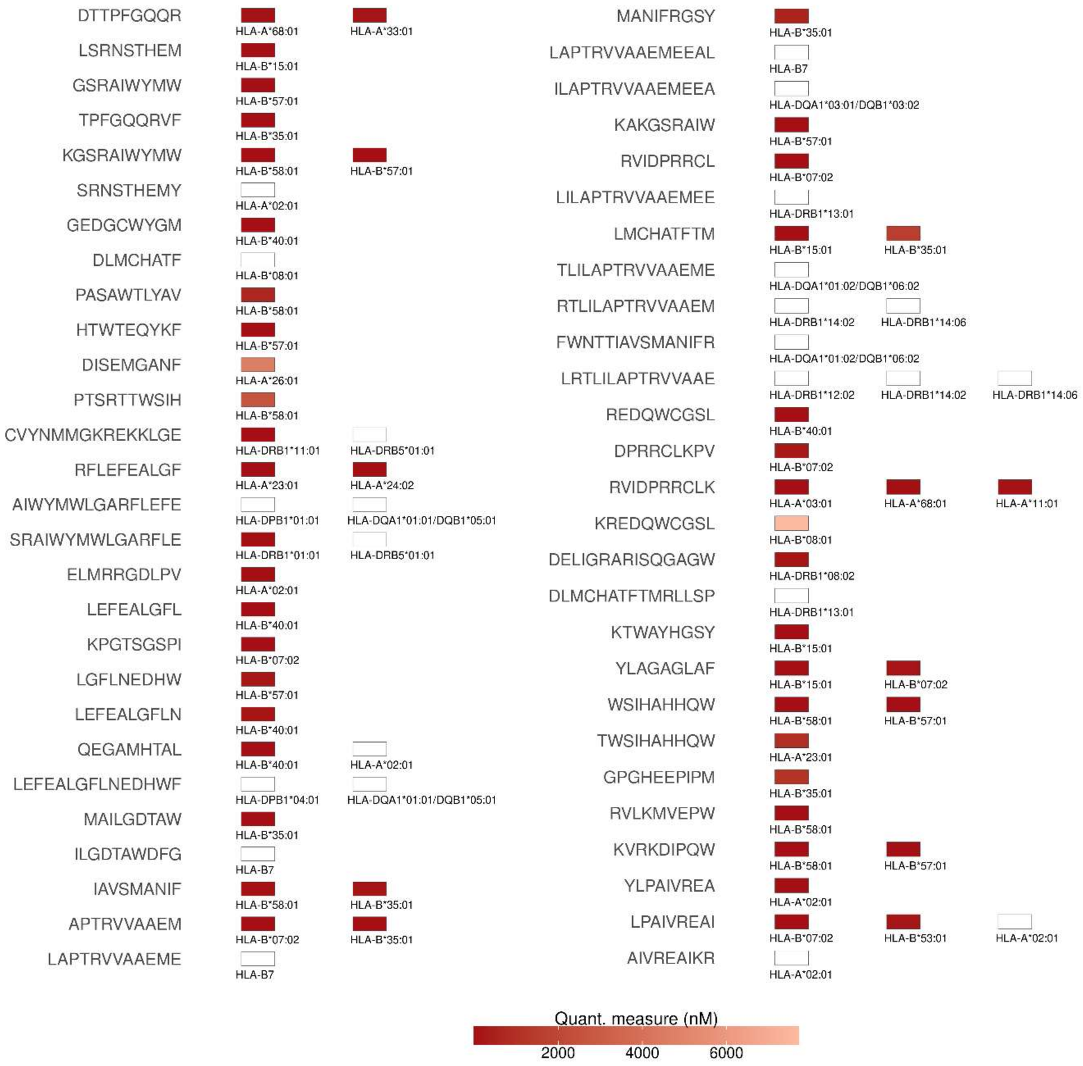

S8 Fig. Reported epitope-HLA binding affinity for the top 55 conserved epitopes (Fig. 3) in the context of cognate HLA alleles. The quantitative measure, IC50 values, of epitope-MHC binding was extracted from positive MHC assays corresponding to these epitopes in the IEDB database. Color of the cells represent the strength of the binding affinity. White cells represent epitope-HLA complexes for which no binding measurements were reported. (PDF)
