# Supplementary material for: Cross-serotypically conserved epitope recommendations for a universal T cell-based dengue vaccine
Source: PLoS Negl Trop Dis. 2020 Sep 21;14(9):e0008676. doi: 10.1371/journal.pntd.0008676 (PMC7529213; doi:10.1371/journal.pntd.0008676)
Supplement: Attachment — Submitted filename: PLOS-NTD_DENV-TcellVX_ManuscriptReview_25Mar2020.docx [file pntd.0008676.s018.docx]

The authors looked at pan-serotypical conserved epitopes on DENV while avoid serotype-specific epitopes by comparing HLA profiles from DENV patients. They identified epitopes conserved between three serotypes within nonstructural proteins. This *in silico* workup is highly relevant to designing a pan-Dengue virus vaccine to drive a T-cell response using epitope-centric, experimentally identified Dengue virus specific T cell epitopes from previous works. The work should be published; however, this reviewer would like to see the following from the workup.

Major revision:

- Added discussion on how an NS5 and NS3 T-cell vaccine could be effectively utilized. Ideally, if the paper was focusing on driving the vaccine to be created, they do little to describe how one should be. I.e., the title of the paper “Robust candidates for a universal T cell vaccine against Dengue virus” is quite misleading. The authors identify robust epitopes that T cells have recognized against dengue but do little in terms of laying out what type of vaccine platforms could elicit such a response, and how these platforms could be programmed antigenically for these suggested epitopes. The authors over-sell these identified epitopes without suggesting how best to use them in a vaccination approach. Please either address these approaches in your discussion that could be viable next steps, or tone down your title and discussion to focus more on the epitope mapping that could inform vaccination strategies.

Minor revision:

- A comparison of these leading epitopes to other flaviviruses, it would be informative to see if these epitopes are conserved across genera.
