# Supplementary material for: Cross-serotypically conserved epitope recommendations for a universal T cell-based dengue vaccine
Source: PLoS Negl Trop Dis. 2020 Sep 21;14(9):e0008676. doi: 10.1371/journal.pntd.0008676 (PMC7529213; doi:10.1371/journal.pntd.0008676)
Supplement: Attachment — Submitted filename: Comments to PNTD.docx [file pntd.0008676.s019.docx]

**Comments to PNTD**

Using a computational approach to identify conserved epitope sequences among the four DENV serotypes, and the global distribution of HLA alleles, the authors selected a limited number of DENV T cell epitopes having identical sequences across at least 3 DENV serotypes, with a high global population coverage. The main message of the article is the design of a new vaccine sequence, containing only the most conserved and potentially antigenic epitopes, as a universal T cell-based vaccine against DENV infection.

Major comments:

This approach, based on the selection of already mapped DENV T cell epitopes, allows the identification the most conserved peptides among the four DENV serotypes. However, it remains to demonstrate that the combination of the 17 T cell epitopes, selected for the highest population coverage, can induce a significant T cell response, in the context of the cognate HLA alleles. This demonstration could be performed using DNA immunization in transgenic mice expressing different HLA class I or class II alleles.

Several references highlighting the protective role of T cells during DENV infection, or identifying immunodominant T cell epitopes as targets for CD8 T cell activation are lacking and should be added (Rivino, Expert Review of Vaccines, 2016; Roth et al., Front. In Immunol., 2019). The last reference (Roth et al., 2019) is lacking in the introduction, fourth paragraph: “Thus far, only a few studies have attempted to identify protein regions…that may be targeted by T cell-based vaccines”.

Strikingly, the S1 file and S2 Table are not complete: several epitopes already identified for their capacity to induce a strong T cell response in HLA class I transgenic mice are not mentioned or included in the list of this Suppl. files. More specifically, the HLA-A*0201- and -B*0702-restricted peptides NS5 318-334 and NS3 593-607, respectively, as well as peptide 56, (Nascimento et al., 2013, Pols NTD, Table 1 and Roth et al., Table 1, respectively) are not in the S1 file nor in the S2 Table. This should be corrected.

Finally, by computing several T cell epitopes, some of them being immunodominant or subdominants in the context of a virus sequence does not necessarily means that these peptides are strongly immunogenic, in the context of a different protein sequence, such as a universal T cell vaccine sequence. Knowing that immunodominance depends on the other antigenic peptides expressed by a protein, a more complete discussion, including references on this particular topic should be addressed. Notably, it would be helpful to have a more detailed picture of the strength of T cell response against the selected and conserved DENV T cell epitopes. A range of the known strength in T cell responses against top pan-serotypically conserved DENV T cell epitopes should be added in the Fig. 3.

Minor points:

In the abstract, the sentence “Recent experimental studies suggest that an effective vaccine against DENV should elicit a strong T cell response against all serotypes, which could be achieved by directing T cell responses towards pan-serotypically conserved epitopes, while avoiding serotype-specific epitopes”, is confusing, as it contains 3 messages. The first message is: An effective vaccine should target T cells. The second one is: To target the 4 DENV serotypes, conserved epitopes should be used, whereas the third message is: Serotype-specific epitopes should be discarded. However, the paper does not address the issue of vaccination in naïve individuals, for which a primary immunization with only conserved epitopes could induce a significant T cell response. Is there any evidence of a detectable T cell response, in naïve individuals, to conserved T cell epitopes? While a secondary heterotypic response is mainly directed to epitopes conserved between the primary and secondary serotype, such as a boost immunization, it remains to demonstrate that a primary immunization targeting conserved epitopes is sufficient to induce a strong T cell response. This should be discussed or at least mentioned in the discussion.
