# Supplementary material for: Cross-serotypically conserved epitope recommendations for a universal T cell-based dengue vaccine
Source: PLoS Negl Trop Dis. 2020 Sep 21;14(9):e0008676. doi: 10.1371/journal.pntd.0008676 (PMC7529213; doi:10.1371/journal.pntd.0008676)
Supplement: Attachment — Submitted filename: Response_Letter.docx [file pntd.0008676.s020.docx]

**RESPONSE TO REVIEWERS’ COMMENTS**

**Robust candidates for a universal T cell vaccine against Dengue virus**

**Revised title:**

**Cross-serotypically conserved epitope recommendations for a universal T cell-based Dengue vaccine**

We are gratified by the positive comments and recommendations of all reviewers. We thank the reviewers for the time they devoted to provide detailed and thoughtful reviews, which have helped to improve our paper. Below, we address each of the reviewers’ comments and note the changes made to the manuscript in response in blue.

## **Reviewer 1:**

**Reviewer's Responses to Questions:**

**Methods**

*This is a computational analysis and the authors have clearly described their methodology for reproducibility purposes.*

**Results**

*Yes, the results are presented nicely and adhered to the publication standards.*

**Conclusions**

*The conclusions drawn supported by the results.*

**Summary and General Comments**

*The manuscript is an attempt to analyze the DENV data from ViPR database. The authors compared the sequences and epitopes and suggested conserved patterns that are also recognized as epitopes. The manuscript is quite interesting and performed in a very reproducible manner. Though there are certain comments need to be addressed before recommending the manuscript for publication.*

**Response**:

We thank the reviewer for the positive comments.

*1. The authors used the consensus sequences as standard, however these consensus sequences are not necessarily representing a serotype. As they considered the highest occurring residues at any positions, these residues will be coming from different variants of the same serotype and might not be a part of any individual strain.*

**Response**:

We understand the potential confusion. First, we would like to clarify that we do not use the consensus sequences for computing “epitope conservation”, which is the main focus of our analysis. Specifically, to compute the conservation of epitopes in a specific serotype, each epitope was scanned across all individual DENV sequences (and not the consensus sequence) of that serotype to determine the fraction of sequences that contained an exact match.

Consensus sequences were only used in Fig. 2c (and S1 Fig) for determining the coverage of epitopes across different DENV proteins. That is, only those epitopes that mapped exactly onto the respective consensus sequences were included in determining coverage in Fig. 2c (and S1 Fig). However, we realize that having different mappings can be confusing for readers. To avoid this confusion, we have made the mapping procedure consistent across the whole manuscript and no longer use a consensus sequence based mapping to compute epitope coverage. In the updated manuscript, for computing coverage, we now scan epitopes across all individual DENV sequences of a serotype (similar to the epitope conservation calculation) and epitopes that map onto at least one sequence are included in the coverage calculation.

We have revised the text and reordered the Materials and Methods section, and updated Fig. 2C and S1 Fig. to avoid this potential confusion.

*2. The consideration of a consensus sequence for conservation has diluted the idea of evolution. The variations among the different members of serotype are evolved in different geographical regions and therefore each variation carries an information for epitopes escaped or developed in that region. The same is reflected in Figure 1B as the sequences are retrieved from different geographical regions.*

**Response**:

As indicated in the response to the previous comment, we did not consider the consensus sequences for computing the conservation of the epitopes. We defined the conservation of an epitope as the fraction of the corresponding protein’s sequences that contain the identical epitope sequence. Using this definition, we computed the conservation of all DENV epitopes across each serotype (S1 File) and identified the top 55 conserved epitopes that were highly conserved (>0.9), while considering variants (e.g., escape or regional variants) as different epitopes, and therefore the conservation profiles were computed separately for each epitope/variant. Thus, the top conserved epitopes identified in this study (Fig. 3) are highly conserved globally and have not varied significantly.

We have revised the related text in the Materials and Methods section for clarity.

*3. The authors did a reverse of geographical regions while calculating the population coverage. They considered the world population as a whole and summed up the frequencies of individuals carrying certain alleles while reporting the population coverage. In such a case, if we are designing a vaccine candidate from the consensus sequences, which are dominated in the Asian countries, but taking the population coverage for an allele mostly reported in the European countries.*

**Response**:

As explained above, the top 55 conserved epitopes identified in this work (Fig. 3) are highly conserved across all global DENV sequences which are obtained from different geographical regions (Fig. 1B). Therefore, vaccine targets identified from within this set of epitopes will likely represent highly conserved epitopes of any target population among these geographical regions. However, since the HLA allele distribution may vary among different target populations, the set of epitopes that maximizes population coverage for different target populations may differ. For example, the sets of epitopes that maximize population coverage are different for Brazil, Thailand and Philippines (S7 Fig.), and these sets are different from the one that maximizes coverage for the combined world population (Fig. 5). Thus, for designing a population-specific vaccine (e.g., Fig. 5 and S7 Fig.), identifying the specific subset of epitopes from the identified ones (Fig. 3) that maximizes the population coverage in a target population is important. The provided software can be seamlessly used to identify such epitopes for any target population as shown in S7 Fig. for three different populations.

*4. The authors' justification of that their work is quite different from previous studies is not quite strong.*

*4.1. The larger dataset not necessarily that results are different too.*

*4.2. The stricter criteria in this manuscript might be covered in not that stricter criteria in previous studies.*

*Please provide a fair comparison with previous results.*

**Response**:

The distinguishing features of our work – larger dataset (immunological and sequence data), stricter criterion (i.e., exact mapping) and epitope-centric approach – indeed result in identification of conserved DENV epitopes that are distinct from those reported previously. A detailed comparison of our results with the related studies (mentioned in the Introduction and Discussion sections) is provided below.

We first note that we were able to obtain the exact list of identified peptides/epitopes for two studies [1,2] and we compared these with the list of top 55 conserved epitopes identified in our work. However, for [3] we used the listed 78 “conserved regions” (Table 6 in [3]), as the supplementary material that was indicated to contain a “detailed mapping of predicted T-cell epitopes” corresponding to the 1,551 pan-DENV identified conserved blocks (see footnote of Table 6 in [3]) was not available.

In these three related studies, 44 pan-DENV conserved peptides (9 to 22 residues long) were identified in [1], 78 conserved regions (9 to 139 residues long) were identified in [3], and 46 peptides (15 to 18 residues long) were identified in [2]. We found that none of these matched exactly with the 55 top conserved epitopes identified in our work. However, some of the conserved regions/peptides identified in the previous works (10 peptides from [1] and 14 peptides from [2]), partially overlap with some of the top conserved epitope sequences identified in our work.

This partial overlap is now indicated in a new supplementary table (S5 Table). We have also revised the text in the Discussion section to (i) include this comparison and (ii) to highlight the difference between our epitope-centric approach for identifying conserved DENV epitopes and those adopted by the previous studies.

## **Reviewer 2:**

**Comments**

*The authors looked at pan-serotypical conserved epitopes on DENV while avoid serotype-specific epitopes by comparing HLA profiles from DENV patients. They identified epitopes conserved between three serotypes within nonstructural proteins. This in silico workup is highly relevant to designing a pan-Dengue virus vaccine to drive a T-cell response using epitope-centric, experimentally identified Dengue virus specific T cell epitopes from previous works. The work should be published; however, this reviewer would like to see the following from the workup.*

**Response**:

We thank the reviewer for the positive comments.

**Major point:**

*Added discussion on how an NS5 and NS3 T-cell vaccine could be effectively utilized. Ideally, if the paper was focusing on driving the vaccine to be created, they do little to describe how one should be. I.e., the title of the paper “Robust candidates for a universal T cell vaccine against Dengue virus” is quite misleading. The authors identify robust epitopes that T cells have recognized against dengue but do little in terms of laying out what type of vaccine platforms could elicit such a response, and how these platforms could be programmed antigenically for these suggested epitopes. The authors over-sell these identified epitopes without suggesting how best to use them in a vaccination approach. Please either address these approaches in your discussion that could be viable next steps, or tone down your title and discussion to focus more on the epitope mapping that could inform vaccination strategies.*

**Response**:

This is a very constructive comment. Indeed, the focus of this work is on identifying robust T cell epitopes that can inform design of a T cell-based vaccine against dengue, while the choice of vaccine platform and design of a particular vaccine based on the identified epitopes are specific future directions that we believe the current analysis helps to motivate.

We have now revised the title of the manuscript to better reflect the scope of the work, as follows:

“Cross-serotypically conserved epitope recommendations for a universal T cell-based dengue vaccine”

We have revised the text at multiple places throughout the manuscript to further clarify that this work focuses on identifying robust T cell epitopes as potential targets for a T cell-based dengue vaccine. We have also included references to some vaccine platforms in the Discussion section that may be useful for designing T cell-based dengue vaccines to target the proposed set of epitopes.

**Minor point:**

*A comparison of these leading epitopes to other flaviviruses, it would be informative to see if these epitopes are conserved across genera.*

**Response**:

We agree that examining the conservation of the identified epitopes across the flavivirus genus would be a worthwhile direction to pursue, particularly since there is evidence of cross-reactive T cell epitopes across flaviviruses [4]. We found that, for some of the cross-serotypically conserved epitopes that we identify, T cell responses have also been reported in IEDB database [5] for other flaviviruses (Table R1). However, we believe that performing a systematic study to determine conservation of the identified epitopes across the flavivirus genus would be beyond the scope of this work. We plan to look further into this in a future study.

We have added text in the Discussion section related to this point, highlighting that the framework developed in this study can be extended to systematically analyze and identify epitopes that are conserved across flaviviruses.

| Flavivirus | Epitopes |
| --- | --- |
| Japanese encephalitis virus | APTRVVAAEM (NS3), TPFGQQRVF (NS5) |
| Zika virus | APTRVVAAEM (NS3), RFLEFEALGF (NS5) |
| Yellow fever virus | TPFGQQRVF (NS5), GSRAIWYMW (NS5), KAKGSRAIW (NS5) |
| West Nile virus | RFLEFEALGF (NS5) |

Table R1. Cross-serotypically conserved DENV epitopes identified in this study (Fig. 3) that are also reported in positive T cell assays of samples from human hosts for other flaviviruses. The protein from which each epitope is derived is indicated in the parentheses.

## **Reviewer 3:**

**Reviewer's Responses to Questions:**

**Methods**

*- The objectives of the study are clearly specified, although they remain hypothetical and difficult to test.*

*- The population has been clearly described*

*- The sample size is sufficient, although some eptides mentioned in the litterature are missing (see comments attached)*

*- Statistics are correct*

*- No concerns about ethical or regulatory requirements*

**Results**

*- The analysis presented match the analysis plan*

*- The results are clearly and completely presented*

*- The figures are of sufficient quality*

**Conclusions**

*- The conclusion of the identification for a set of candidate-epitopes for a pan DENV T cell-based vaccine, would require more functional studies to verify the immunogenicity against different DENV serotypes*

*- The limitation of this analysis is not enough clearly stated*

*- With this limitation, the public health relevance is addressed*

**Response**:

We thank the reviewer for the constructive review.

All the identified epitopes in this study have been determined to be immunogenic in humans in prior experimental studies. However, we agree that more experimental studies would be required to test the immune responses elicited following immunization with the proposed set of target epitopes against challenge with different DENV serotypes.

We have added a paragraph in the Discussion section to highlight this limitation of the current study.

**Editorial and Data Presentation Modifications**

*- In Fig. 3 and Fig. 5, strong and weak epitopes should be mentioned. This is an important data to keep in mind, for prospective efficient T cell vaccines.*

**Response**:

Please see our response to Major points 4 and 5 (below) where we address this in detail.

We have now added the information about the number of positive assays and response frequencies corresponding to each epitope in Fig. 3, which also covers the subset of epitopes listed in Fig. 5. We believe this addition has further improved our manuscript.

**Summary and General Comments**

*A more complete discussion, on immunodominant and subdominant epitopes in the context of different protein sequences would reinforce the conclusion of a vaccine-candidate epitope. The question of the hierarchy of epitopes in a whole viral sequence vs a short mosaic sequence is a crucial issue, and should be discussed in more detail.*

**Response**:

We address this comment in response to Major point 4 (below).

**Comments**

*Using a computational approach to identify conserved epitope sequences among the four DENV serotypes, and the global distribution of HLA alleles, the authors selected a limited number of DENV T cell epitopes having identical sequences across at least 3 DENV serotypes, with a high global population coverage. The main message of the article is the design of a new vaccine sequence, containing only the most conserved and potentially antigenic epitopes, as a universal T cell-based vaccine against DENV infection.*

**Response**:

We thank the reviewer for summarizing our work.

We emphasize that the current study proposes a set of target epitopes for a potential T cell-based dengue vaccine. However, the design of a specific vaccine sequence to target these epitopes is non-trivial and depends on multiple factors, including the specific technology employed to design such a vaccine, determining the immunodominance pattern of the identified epitopes in combination [6], etc. We believe that our study would motivate future work in this direction.

In order to better clarify the scope of this study, we have revised the title of the manuscript, as well as the text at several places throughout the manuscript.

**Major points:**

1. *This approach, based on the selection of already mapped DENV T cell epitopes, allows the identification the most conserved peptides among the four DENV serotypes. However, it remains to demonstrate that the combination of the 17 T cell epitopes, selected for the highest population coverage, can induce a significant T cell response, in the context of the cognate HLA alleles. This demonstration could be performed using DNA immunization in transgenic mice expressing different HLA class I or class II alleles.*

**Response**:

All the 17 epitopes proposed in this work as targets for a T cell-based vaccine (Fig. 5) were derived from positive T cell assays of samples from human hosts and were determined as HLA-matched epitopes, as reported in the ViPR database. These epitopes were determined in different experimental studies with most of the epitopes being targeted in multiple donors (Fig. 3). Therefore, these epitopes are known to be immunogenic in humans in the context of cognate HLA alleles, and thus are likely to induce T cell responses upon immunization. We have revised the text in the Introduction and Materials and Methods sections to clarify this important point.

We agree however that further experimental studies are needed to examine the collective T cell responses induced after immunization with the combination of 17 epitopes (e.g., using HLA transgenic mice expressing different HLA alleles). Similarly, carefully designed studies are required to analyze the pattern of epitope immunodominance, which is complex [6] and can be influenced by immunization [7,8]. However, we believe this is beyond the scope of the current study as our objective was to identify a set of cross-serotypically conserved epitopes that can potentially serve as targets for a universal T cell-based dengue vaccine. We have now clarified these points in the Discussion section of the revised manuscript.

2. *Several references highlighting the protective role of T cells during DENV infection, or identifying immunodominant T cell epitopes as targets for CD8 T cell activation are lacking and should be added (Rivino, Expert Review of Vaccines, 2016; Roth et al., Front. In Immunol., 2019). The last reference (Roth et al., 2019) is lacking in the introduction, fourth paragraph: “Thus far, only a few studies have attempted to identify protein regions…that may be targeted by T cell-based vaccines”.*

**Response**:

We have updated the text in the Introduction section and included citations to the references indicated (Refs. [17] and [18] in the updated manuscript are *Rivino, Expert Review of Vaccines, 2016* and *Roth et al., Front. In Immunol.,2019*, respectively).

3. *Strikingly, the S1 file and S2 Table are not complete: several epitopes already identified for their capacity to induce a strong T cell response in HLA class I transgenic mice are not mentioned or included in the list of this Suppl. files. More specifically, the HLA-A*0201- and -B*0702-restricted peptides NS5 318-334 and NS3 593-607, respectively, as well as peptide 56, (Nascimento et al., 2013, Pols NTD, Table 1 and Roth et al., Table 1, respectively) are not in the S1 file nor in the S2 Table. This should be corrected.*

**Response**:

In this study, we focused on the experimentally-determined T cell epitopes reported in positive T cell assays of samples from *human* hosts only. Thus, the S1 file and S2 Table did not include epitopes (including the three epitopes mentioned by the reviewer; listed below) that were experimentally-determined in HLA transgenic mice and not in humans.

(i) SMINGVVKLLTKPWDVV (IEDB ID: 190982) restricted by HLA-A*02:01,
(ii) RPRWLDARTYSDPLA (IEDB ID: 190956) restricted by HLA-B*07:02, and
(iii) SMVNGVVKL (IEDB ID: 59771) restricted by HLA-A*02:01

Nevertheless, the reviewer’s comment has motivated us to analyze all the experimentally-determined T cell epitopes from HLA transgenic mice reported in the ViPR database. We examined the conservation profile of such (213) epitopes across dengue serotypes (as outlined in Methods). We have added a new table with these results in the supplement (S3 File). Of these 213 epitopes, only 9 were found to be highly conserved (conservation greater than 0.9) in at least 3 serotypes (S9 Fig.). Interestingly, among these 9 epitopes, 5 were also reported in human hosts and thus were already present in our list of 55 cross-serotypically conserved epitopes (Fig. 3). These epitopes are as follows:

(i) APTRVVAAEM (IEDB ID: 150137) restricted by HLA-B*07:02,
(ii) ELMRRGDLPV (IEDB ID: 150184) restricted by HLA-A*02:01,
(iii) RVIDPRRCL (IEDB ID: 56309) restricted by HLA-B*07:02,
(iv) SRAIWYMWLGARFLE (IEDB ID: 150597) restricted by HLA-DRB1*01:01, and
(v) LPAIVREAI (IEDB ID: 38400) restricted by HLA-B*07:02

The 4 remaining conserved epitopes from HLA transgenic mice data, which have not been reported in human hosts, are as follows:

(i) MYADDTAGW (IEDB ID: 43033) restricted by HLA-A*24:02,
(ii) WYMWLGARFL (IEDB ID: 186540) restricted by HLA-A*24:02,
(iii) WYMWLGARF (IEDB ID: 73284) restricted by HLA-A*24:02, and
(iv) WPKSHTLWSNGVLES (IEDB ID: 191028) restricted by HLA-DR3

We have updated the Results section accordingly and have included two new supplementary items related to this analysis (S3 File and S9 Fig).

4. *Finally, by computing several T cell epitopes, some of them being immunodominant or subdominants in the context of a virus sequence does not necessarily means that these peptides are strongly immunogenic, in the context of a different protein sequence, such as a universal T cell vaccine sequence. Knowing that immunodominance depends on the other antigenic peptides expressed by a protein, a more complete discussion, including references on this particular topic should be addressed.*

**Response**:

We thank the reviewer for raising this important point. As discussed in response to the Major point 1, all the top conserved epitopes identified in this study are immunogenic, reported in positive T cell assays of samples from human hosts. However, we agree that the immunodominance (or strong immunogenicity) of the T cell epitopes following vaccination would depend upon the vaccine sequence and presence of other epitopes. For example, it has been reported that immunization can alter the immunodominance of epitopes [8,9] and targeting of individually sub-dominant (or weakly immunodominant) epitopes may even be protective [10,11].

As suggested, we have now added a detailed discussion and references related to this point in the Discussion section of the revised manuscript.

5. *Notably, it would be helpful to have a more detailed picture of the strength of T cell response against the selected and conserved DENV T cell epitopes. A range of the known strength in T cell responses against top pan-serotypically conserved DENV T cell epitopes should be added in the Fig. 3.*

**Response**:

Indeed, information about the strength of T cell responses against the identified top conserved epitopes would be helpful. These T cell responses have been studied in multiple experiments, as indicated in the IEDB database. However, we found that many such studies do not report the specific strength of the T cell responses measured against each epitope. Moreover, due to differences in experimental details (e.g., experiment design, subjects, response measurement, etc.), it would be difficult to directly and meaningfully compare the reported quantitative values across these studies. Instead, we have now updated Fig. 3 by adding details about the response frequencies and number of positive assays and donors associated with each of the top conserved epitopes. These can be used as a proxy to quantitatively compare the immunodominance of the identified epitopes across multiple hosts [12,13]. Furthermore, strong peptide-HLA binding affinity is important for a strong T cell response [14] and we note that most of the identified epitopes, for which HLA binding measurements have been reported, have very strong affinities with cognate HLA molecules (S8 Fig.).

In addressing this point, we have updated Fig. 3 and the text related to it in the Results section, and added a new supplementary figure (S8 Fig.).

**Minor points:**

1. *In the abstract, the sentence “Recent experimental studies suggest that an effective vaccine against DENV should elicit a strong T cell response against all serotypes, which could be achieved by directing T cell responses towards pan-serotypically conserved epitopes, while avoiding serotype-specific epitopes”, is confusing, as it contains 3 messages.*

*The first message is: An effective vaccine should target T cells. The second one is: To target the 4 DENV serotypes, conserved epitopes should be used, whereas the third message is: Serotype-specific epitopes should be discarded.*

*However, the paper does not address the issue of vaccination in naïve individuals, for which a primary immunization with only conserved epitopes could induce a significant T cell response.*

*Is there any evidence of a detectable T cell response, in naïve individuals, to conserved T cell epitopes?*

2. *While a secondary heterotypic response is mainly directed to epitopes conserved between the primary and secondary serotype, such as a boost immunization, it remains to demonstrate that a primary immunization targeting conserved epitopes is sufficient to induce a strong T cell response. This should be discussed or at least mentioned in the discussion.*

**Response**:

1. We agree that vaccines targeting the conserved epitopes of DENV could elicit T cell response in naïve individuals. We find that such responses have been reported following vaccination with two LAVs. Specifically, following vaccination with monovalent or tetravalent DLAV (TV003) [15] and with tetravalent TAK-003 [16], T cell responses have been reported against 9 and 11 of the 55 identified epitopes, respectively.
2. We also agree that it has yet to be demonstrated that a primary immunization targeting the specific conserved epitopes is sufficient to induce a strong T cell response.

As suggested, we have added discussion related to these two points in the Discussion section of the revised manuscript. We have also added a new supplementary table (S4 Table) which lists the specific epitopes against which T cell responses were reported in naïve individuals.

# **References**

1.	Khan AM, Miotto O, Nascimento EJM, Srinivasan KN, Heiny AT, Zhang GL, et al. Conservation and variability of dengue virus proteins: Implications for vaccine design. PLoS Negl Trop Dis. 2008;2.

2.	Nascimento EJM, Mailliard RB, Khan AM, Sidney J, Sette A, Guzman N, et al. Identification of conserved and HLA promiscuous DENV3 T-cell epitopes. PLoS Negl Trop Dis. 2013;7. doi:10.1371/journal.pntd.0002497

3.	Olsen L. Conservation analysis of dengue virus T-cell epitope-based vaccine candidates using peptide block entropy. Front Immunol. 2011;2: 1–15. doi:10.3389/fimmu.2011.00069

4.	Slon Campos JL, Mongkolsapaya J, Screaton GR. The immune response against flaviviruses. Nat Immunol. 2018;19: 1189–1198. doi:10.1038/s41590-018-0210-3

5.	Vita R, Mahajan S, Overton JA, Dhanda SK, Martini S, Cantrell JR, et al. The immune epitope database (IEDB): 2018 update. Nucleic Acids Res. 2019;47: D339–D343. doi:10.1093/nar/gky1006

6.	Weiskopf D, Angelo MA, Sidney J, Peters B, Shresta S, Sette A. Immunodominance changes as a function of the infecting dengue virus serotype and primary versus secondary infection. J Virol. 2014;88: 11383–11394. doi:10.1128/JVI.01108-14

7.	Welsh RM, Che JW, Brehm MA, Selin LK. Heterologous immunity between viruses. Immunol Rev. 2010;235: 244–266. doi:10.1111/j.0105-2896.2010.00897.x

8.	Im E-J, Hong JP, Roshorm Y, Bridgeman A, Létourneau S, Liljeström P, et al. Protective efficacy of serially up-ranked subdominant CD8+ T cell epitopes against virus challenges. Douek DC, editor. PLoS Pathog. 2011;7: e1002041. doi:10.1371/journal.ppat.1002041

9.	Remakus S, Rubio D, Ma X, Sette A, Sigal LJ. Memory CD8 + T cells specific for a single immunodominant or subdominant determinant induced by peptide-dendritic cell immunization protect from an acute lethal viral disease. J Virol. 2012;86: 9748–9759. doi:10.1128/JVI.00981-12

10.	Ruckwardt TJ, Luongo C, Malloy AMW, Liu J, Chen M, Collins PL, et al. Responses against a subdominant CD8 + T cell epitope protect against immunopathology caused by a dominant epitope. J Immunol. 2010;185: 4673–4680. doi:10.4049/jimmunol.1001606

11.	Frahm N, Kiepiela P, Adams S, Linde CH, Hewitt HS, Sango K, et al. Control of human immunodeficiency virus replication by cytotoxic T lymphocytes targeting subdominant epitopes. Nat Immunol. 2006;7: 173–178. doi:10.1038/ni1281

12.	Gianfrani C, Oseroff C, Sidney J, Chesnut RW, Sette A. Human memory CTL response specific for influenza A virus is broad and multispecific. Hum Immunol. 2000;61: 438–452. doi:10.1016/S0198-8859(00)00105-1

13.	Tian Y, Grifoni A, Sette A, Weiskopf D. Human T cell response to dengue virus infection. Front Immunol. 2019;10: 1–9. doi:10.3389/fimmu.2019.02125

14.	Feltkamp MCW, Vierboom MPM, Kast WM, Melief CJM. Efficient MHC class I-peptide binding is required but does not ensure MHC class I-restricted immunogenicity. Mol Immunol. 1994;31: 1391–1401. doi:10.1016/0161-5890(94)90155-4

15.	Weiskopf D, Angelo MA, Bangs DJ, Sidney J, Paul S, Peters B, et al. The human CD8 + T cell responses induced by a live attenuated tetravalent dengue vaccine are directed against highly conserved epitopes. Diamond MS, editor. J Virol. 2015;89: 120–128. doi:10.1128/JVI.02129-14

16.	Waickman AT, Friberg H, Gargulak M, Kong A, Polhemus M, Endy T, et al. Assessing the diversity and stability of cellular immunity generated in response to the candidate live-attenuated dengue virus vaccine TAK-003. Front Immunol. 2019;10: 1–13. doi:10.3389/fimmu.2019.01778
